# Palladium‐Catalyzed Direct Stereoselective Synthesis of Deoxyglycosides from Glycals

**DOI:** 10.1002/anie.201612071

**Published:** 2017-02-17

**Authors:** Abhijit Sau, Ryan Williams, Carlos Palo‐Nieto, Antonio Franconetti, Sandra Medina, M. Carmen Galan

**Affiliations:** ^1^ School of ChemistryUniversity of BristolCantock's CloseBristolBS8 1TSUK

**Keywords:** acetals, asymmetric catalysis, deoxyglycosides, glycosylation, palladium

## Abstract

Palladium(II) in combination with a monodentate phosphine ligand enables the unprecedented direct and α‐stereoselective catalytic synthesis of deoxyglycosides from glycals. Initial mechanistic studies suggest that in the presence of *N*‐phenyl‐2‐(di‐tert‐butylphosphino)pyrrole as the ligand, the reaction proceeds via an alkoxy palladium intermediate that increases the proton acidity and oxygen nucleophilicity of the alcohol. The method is demonstrated with a wide range of glycal donors and acceptors, including substrates bearing alkene functionalities.

The ability to perform O‐glycosylation reactions in a catalytic and stereoselective manner is one of the main remaining challenges in carbohydrate chemistry. Biologically relevant chiral acetals such as deoxyhexoses are prominent components of natural products,^1^ and present a significant synthetic challenge because of the lack of substituents at C‐2 to direct the nucleophile approach (Scheme 1). Thus, efforts by our group^2^ and others^3^ have been devoted to achieving the stereoselective synthesis of these compounds. Recent years have seen a steady increase in the application of transition‐metal catalysis to oligosaccharide synthesis,^4^ since the careful choice of ligand/transition‐metal combination can offer significant improvements over traditional methods in terms of atom economy, high yields, and control of anomeric selectivity. The palladium‐catalyzed direct activation of 1,2‐unsaturated glycals to yield the corresponding 2,3‐unsaturated Ferrier products with good to excellent selectivity is well established and it is believed to proceed via π‐allyl intermediates.4b,4c, 5

**Scheme 1:**
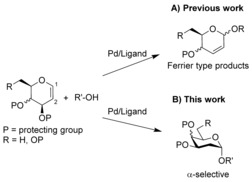
A) Pd‐catalyzed synthesis of 2,3‐unsaturated glycosides. B) Pd‐catalyzed synthesis of deoxyglycosides.

Herein, we describe the unprecedented Pd‐catalyzed stereoselective synthesis of deoxyglycosides directly from glycals. Products resulting from addition of the proton and alkoxide nucleophile across the carbon–carbon double bond are formed when monodentate *N*‐phenyl‐2‐(di‐*tert*‐butylphosphino)pyrrole is employed as the ligand. This outcome is likely derived from an increase in affinity of palladium towards the OH nucleophile, which allows the reaction to proceed through an alkoxypalladation‐type mechanism to yield the glycoside with high α‐stereocontrol.

The ligand in a transition‐metal‐catalyzed reaction plays a key role in stabilizing and activating the central metal atom and fine‐tuning the selectivity of the transformation. Initial experiments began with the screening of a series of commercial mono‐ and bidentate phosphine ligands (**L1–L8**, 30 mol %) for their ability to promote the stereoselective glycosylation of perbenzylated galactal **1 a** with glucoside acceptor **2 a** 6 in the presence of 10 mol % of Pd(MeCN)_2_Cl_2_ in CH_2_Cl_2_ at 50 °C. As summarized in Table 1, only monodentate ligands **L1**, **L2**, and **L3** with Pd^II^ were able to activate the glycal, and **3 a** was obtained in low to moderate yield (37–75 %), with **L2** giving the best α‐selectivity (>30:1; Table 1, entries 2–4). Interestingly, no 2,3‐unsaturated Ferrier product was observed in any of the reactions when the phosphine ligand was present, while reactions in the absence of ligand yielded an inseparable mixture of Ferrier and glycoside products. Next, we decided to explore solvent effects, reaction temperature, and catalyst loading. The use of acetonitrile or toluene was detrimental to the yield (entries 10 and 11), and the reaction rate significantly diminished at room temperature in CH_2_Cl_2_ (entry 13). Finally, increasing the Pd^II^ loading to 25 mol % gave optimal yields and α‐stereocontrol (90 % and >30:1 α*/*β) within 17 hours [entry 14 vs. entry 3 (10 mol %) and entry 12 (20 mol %)]. To further investigate the effect of the catalyst, a series of different Pd^II^ catalysts were also screened in the glycosylation reaction in the presence of **L2** (Table 1, entries 15–19). It was found that removing or replacing the Cl counterion with either a *p*‐toluenesulfonate, tetrafluoroborate, or trifluoromethanesulfonate was detrimental to the yield, while replacement of acetonitrile with benzonitrile (entry 15) did not affect the yield or stereocontrol. It is important to note that reactions with **L2** in the absence of Pd did not work.

**Table 1:** Initial catalyst screen for the glycosylation of galactal **2 a**. 
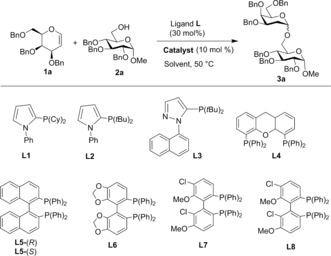

Entry	Ligand	Catalyst	Time [h]	Solvent	Yield [%]^[b]^	α/β^[b]^
1	–	Pd(MeCN)_2_Cl_2_	24	CH_2_Cl_2_	–^[a]^	N/A
2	**L1**	Pd(MeCN)_2_Cl_2_	24	CH_2_Cl_2_	37	14:1
3	**L2**	Pd(MeCN)_2_Cl_2_	24	CH_2_Cl_2_	75	>30:1
4	**L3**	Pd(MeCN)_2_Cl_2_	24	CH_2_Cl_2_	45	10:1
5	**L4**	Pd(MeCN)_2_Cl_2_	24	CH_2_Cl_2_	0	N/A
6	**L5**	Pd(MeCN)_2_Cl_2_	24	CH_2_Cl_2_	0	N/A
7	**L6**	Pd(MeCN)_2_Cl_2_	24	CH_2_Cl_2_	0	N/A
8	**L7**	Pd(MeCN)_2_Cl_2_	24	CH_2_Cl_2_	0	N/A
9	**L8**	Pd(MeCN)_2_Cl_2_	22	CH_2_Cl_2_	0	N/A
10	**L2**	Pd(MeCN)_2_Cl_2_	24	MeCN	54	25:1
11	**L2**	Pd(MeCN)_2_Cl_2_	24	toluene	39	>30:1
12	**L2**	Pd(MeCN)_2_Cl_2_	17	CH_2_Cl_2_	82^[c]^	>30:1
13	**L2**	Pd(MeCN)_2_Cl_2_	17	CH_2_Cl_2_	27^[c,d]^	>30:1
14	**L2**	Pd(MeCN)_2_Cl_2_	17	CH_2_Cl_2_	90^[e]^	>30:1
15	**L2**	Pd(PhCN)_2_Cl_2_	17	CH_2_Cl_2_	86	>30:1
16	**L2**	Pd(CH_3_CN)_2_(OTs)_2_	17	CH_2_Cl_2_	58	>30:1
17	**L2**	Pd(CH_3_CN)_4_(OTf)_2_	17	CH_2_Cl_2_	N/A^[f]^	N/A
18	**L2**	Pd(CH_3_CN)_4_(BF_4_)_2_	17	CH_2_Cl_2_	32	>20:1
19	**L2**	Pd(OAc)_2_	17	CH_2_Cl_2_	0	N/A
20	**L2**	–	17	CH_2_Cl_2_	0	N/A

[a] Reactions in the absence of ligand yielded a complex mixture on products. [b] Determined by crude ^1^H NMR. [c] Reaction with 20 mol % Pd in CH_2_Cl_2_ (yield of isolated product shown) [d] Reaction at RT. [e] Reaction with 25 mol % Pd in CH_2_Cl_2_ (yield of isolated product shown). [f] Inseparable complex mixture of products. N/A=not applicable.

Having established the optimum reaction conditions, our attention then turned to exploring the substrate scope of the coupling reaction between **1 a** and a range of OH nucleophiles (**2 b**–**2 i**; Table 2). In all cases, the reactions proceeded smoothly and in good to excellent yields and α‐selectivity, thus demonstrating that the catalytic system tolerates the presence of common alcohol and amine protecting groups such as acetals, ethers, esters, and carbamates. Glycosylations with primary alcohols **2 b**–**2 d**, thioglycoside **2 e**, and Boc‐protected serine **2 h** afforded the corresponding glycoside products in 69–96 % yield within 17 h and with α/β ratios ranging from more than 30:1 to α only (Table 2, entries 1–4 and 7). Similarly, reactions with secondary alcohols such as glycosides **2 f** and **2 g** or *N*‐hydroxysuccinimide **2 i** also afforded the desired products in good yields (73–85 %) and with high α‐selectivity (α/β ratio ranging from >30:1 to α only; entries 5, 6, and 8).

**Table 2:** Acceptor scope of glycosylation reactions with galactal **2 a**. 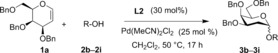

Entry	ROH		Yield [%]^[a]^	α/β^[b]^
1	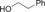	**2 b**	69	α only
2	BnOH	**2 c**	96	>30:1
3	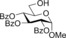	**2 d**	82	>30:1
4	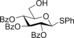	**2 e**	84	>30:1
5	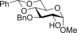	**2 f**	73	α only
6	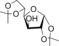	**2 g**	74	>30:1
7	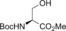	**2 h**	88	α only
8	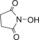	**2 i**	85	α only

[a] Yield of isolated product. [b] Determined by crude ^1^H NMR.

To investigate the scope of the glycal donor, a series of differentially protected galactals (**1 b**–**1 f**), glucals (**4 a** and **4 b**) and l‐rhamnal (**5**) bearing methyl, acetate, benzyl, silyl ether, or siloxane protecting groups were prepared and subjected to the reaction conditions with **2 a** (bearing a primary OH) or **2 f** (bearing a secondary OH) as nucleophile acceptors (Table 3). Pleasingly, high yields (68–86 %) and excellent selectivity for α‐linked glycosides (α/β ratio of >10:1 to >30:1 ) were obtained in all examples, with the exception of peracetylated galactal **1 e** (entry 4). Although we show that ester groups are tolerated elsewhere in the glycal donor (Table 3, entry 1), the presence of a deactivating ester group at C‐3 in close proximity to the reacting double bond is known to significantly decrease the reactivity of the donor.2a, 7 Encouragingly, the reaction was also applicable to glycosylations with glucal substrates, and reactions of 3,4‐*O*‐siloxane‐protected **4 a** 2c and **4 b** 2c with primary and secondary OH nucleophiles **2 a** or **2 f** afforded the corresponding glycosides **7 a**, **7 b**, and **7 f** with high α‐stereocontrol (α/β from >30:1 to α only) and good yields (68–86 %, entries 6–8). 2,6‐Dideoxyglycosides are also an important class of compounds and their stereoselective synthesis is further complicated by a lack of oxygen substituents at both C‐2 and C‐6.^8^ Excitingly, activation of 3,4‐*O*‐siloxane‐protected l‐rhamnal **5** afforded **8** in 75 % yield within 17 h and with an α/β ratio 10:1 (entry 9). These results further demonstrate that the catalytic system works well across a range of reactivity profiles in both the glycal moiety and nucleophile acceptor.

**Table 3:** Reaction of glycals **1 b**–**1 f**, **4 a**, **4 b**, and **5** with model glycosyide acceptors **2 a** or **2 b**. 
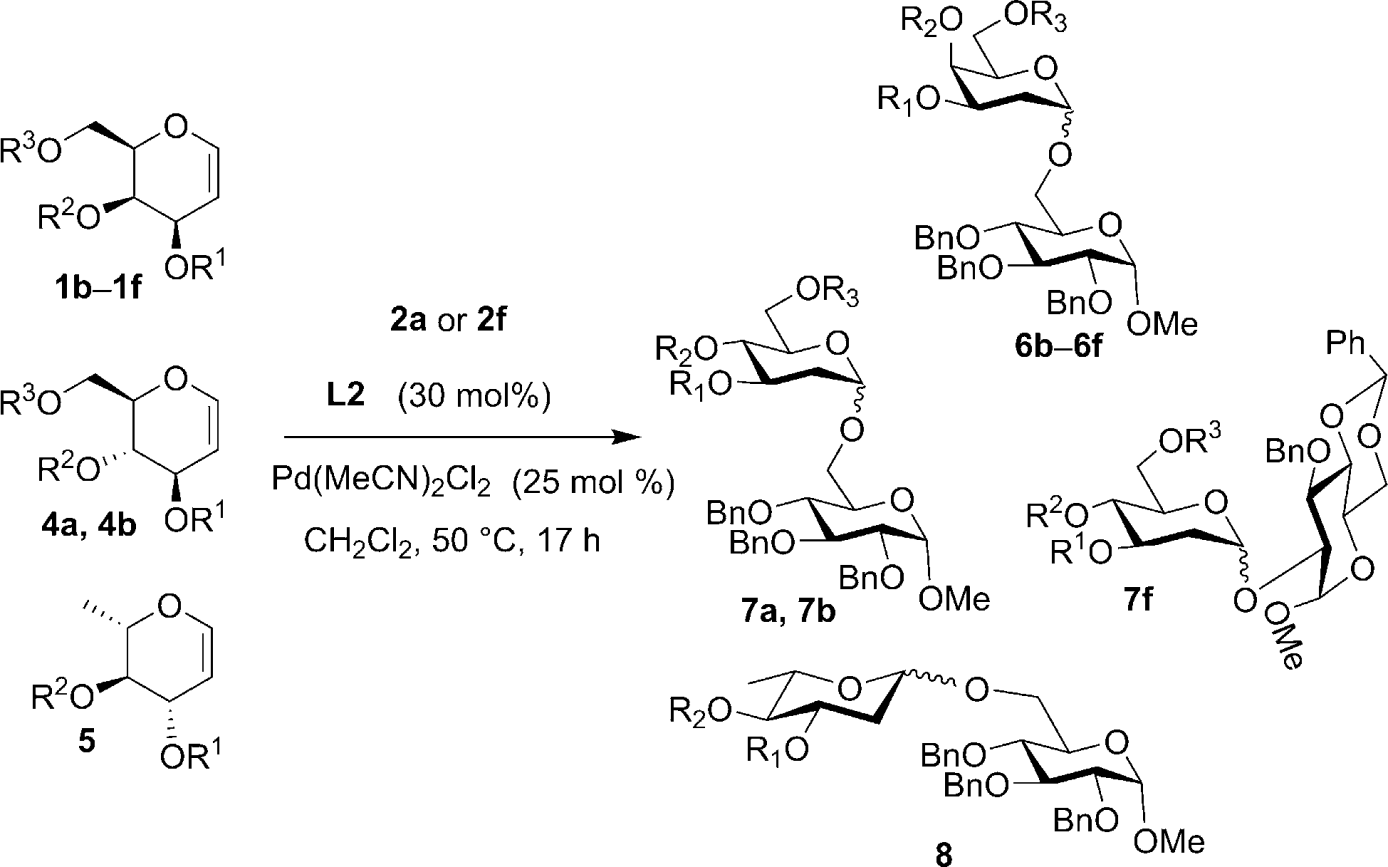

Entry		R^1^	R^2^	R^3^	Product	Yield [%]^[a]^	α/β^[b]^
1	**1 b**	Bn	Bn	Ac	**6 b**	82	>21:1
2	**1 c**	TBS	TBS	TBS	**6 c**	82	>30:1
3	**1 d**	Me	Me	Me	**6 d**	78	>30:1
4	**1 e**	Ac	Ac	Ac	**6 e**	0	N/A
5	**1 f**	MOM	MOM	MOM	**6 f**	85	>30:1
6	**4 a**	O[Si(*i*Pr)_2_]_2_		Bn	**7 a**	86	>30:1
7	**4 b**	O[Si(*i*Pr)_2_]_2_		TIPS	**7 b**	75	>30:1
8	**4 b**	O[Si(*i*Pr)_2_]_2_		TIPS	**7 f**	68^[c]^	>30:1
9	**5**	O[Si(*i*Pr)_2_]_2_		–	**8**	75	10:1

[a] Yield of isolated product. [b] Determined by ^1^H NMR. [c] Reaction was carried out for 27 h.

To probe the mechanism of our reaction, a 4:1 α/β‐anomeric mixture of **3 a** was subjected to the reaction conditions in the presence of acceptor **2 a** and gave no change in the anomeric ratio, thus indicating that the high α‐selectivity is not the result of anomerization (Figure S3 in the Supporting Information). Reaction with deuterated galactal **9** yielded disaccharide **10** (90 % yield) with the newly formed bonds *cis* to each other (Scheme 2 A and Figure S1 in the Supporting Information). Moreover, glycosylation between galactal **1 a** and CD_3_OD yielded α‐linked D_3_‐methyl 2‐D‐glycoside **11**, in which deuterium from the nucleophile is incorporated equatorially at C‐2, (Scheme 2 B and Figure S2). These results confirm the OH nucleophile as the H source and that both the C−H/D and the C−O bond formation steps are preferentially *syn*‐diastereoselective. Moreover, addition of 1‐phenylpyrrole or K_2_CO_3_ (0.3 equiv) as exogenous bases yielded only starting material, thus suggesting that sequestering acid generated during the reaction is detrimental to product formation.

**Scheme 2:**
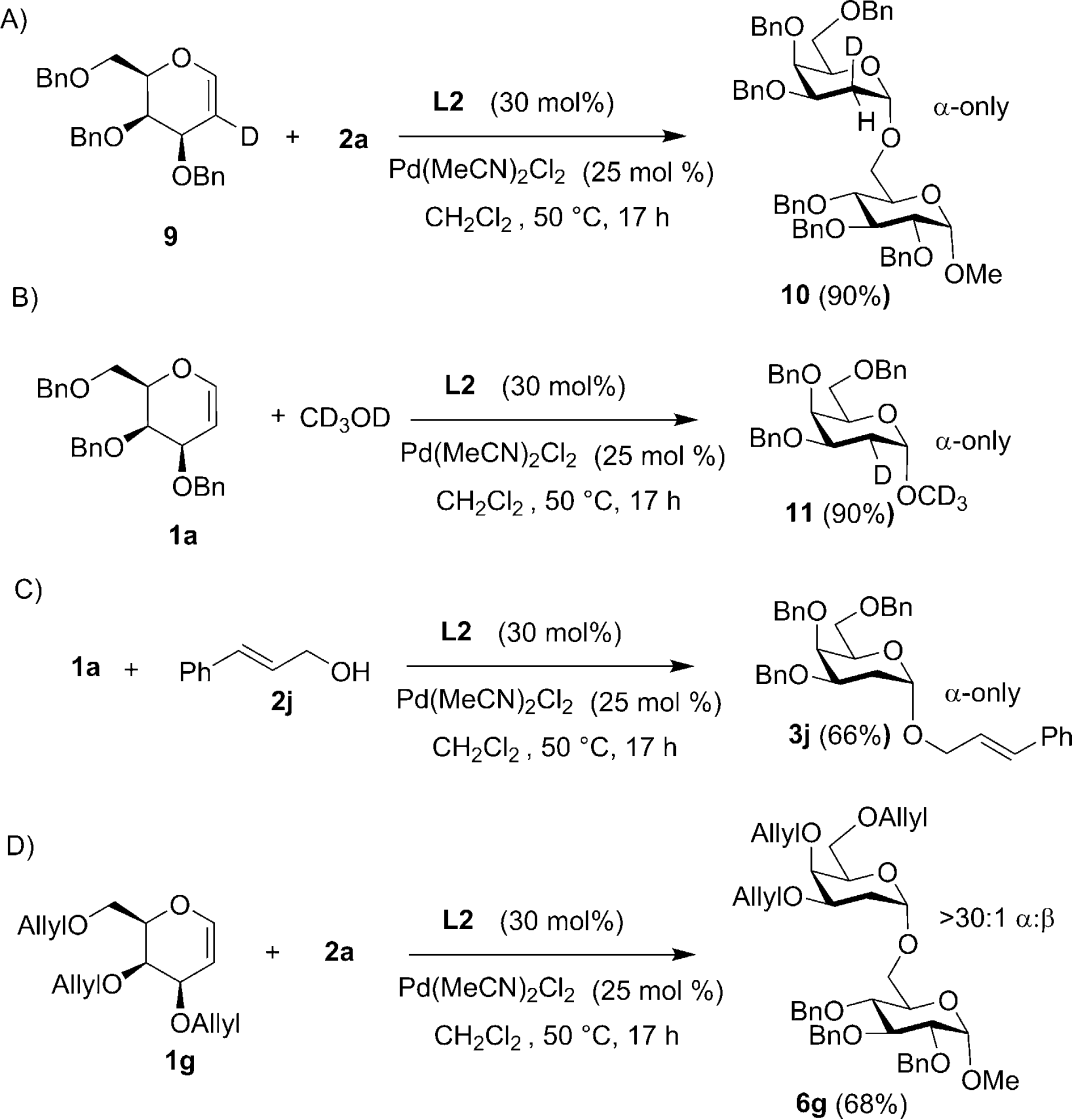
Mechanistic studies with glycal donors **9**, **1 a**, and **1 g**.

^1^H NMR spectroscopy studies in CD_2_Cl_2_ of mixtures of Pd(MeCN)_2_Cl_2_, ligand **L2**, and glycoside donor **1 a** did not show any changes in the spectra, while mixtures of Pd(MeCN)_2_Cl_2_ and **1 a** in the absence of **L2** clearly showed downfield shifts associated with alkene protons in **1 a** (from *δ*=6.37 ppm to 6.20 and 6.03 ppm), thus suggesting that the presence of phosphine **L2** prevents the interaction between Pd and the glycal enol ether. Furthermore, NMR mixtures of Pd(MeCN)_2_Cl_2_, ligand **L2**, and glycoside acceptor **2 a** showed downfield shifts for the OH signal in **2 a** from *δ*=1.86 ppm to *δ*=2.00 ppm, while no spectral changes were observed in NMR mixtures of **L2** and **2 a** in the absence of Pd^II^ (See the Supporting Information for details). Furthermore, glycosylation reactions between **1 a** and cinnamyl alcohol **2 j**, which bears a double bond, or allyl‐protected galactal **1 g** and **2 a**, proceeded smoothly to the corresponding α‐glycosides **3 j** (66 %) and **6 g** (68 %) with excellent stereocontrol and an α/β ratio of more than 30:1 (Scheme 2 C,D). These results further demonstrate that phosphine ligand **L2** is able to fine‐tune the palladium reactivity towards alkoxypalladation, rather than palladium‐mediated activation of the alkene. NMR spectroscopy was then used to try to identify reaction intermediates from the glycosylation between **1 a** and **2 c** at 50 °C. Aliquots were taken from the reaction at different time points and the samples quenched by cooling to 0 °C prior to analysis.^9^ Only anomeric signals (H and C) corresponding to starting material and product were observed (Figures S6, S7), thus suggesting that the reaction proceeds via short‐lived intermediates.

While a detailed mechanism awaits further investigation, our findings suggest that in the presence *N*‐phenyl‐2‐(di‐*tert*‐butylphosphino)‐pyrrole (**L2**), palladium‐catalyzed coupling of glycals with alcohol nucleophiles involves the initial insertion of Pd into the RO−H bond, rather than the traditional pathway of palladium‐mediated alkene activation,^3^ to produce the alkoxypalladium species (**A**) with concomitant H^+^ release from the OH nucleophile (Scheme 3).^10^ Proton‐catalyzed glycal activation can now take place from the less hindered face, which leads to the formation of a transient oxocarbenium ion (**B**).^11^ Although two diastereomeric half‐chair conformers are possible, the depicted conformer (**B**) is favored,^12^ which quickly reacts with the activated oxygen nucleophile in (**A**) in a stereoselective manner to give the corresponding α‐glycoside. This pathway is preferred due to steric effects, the anomeric effect, and a chair‐like transition state, thus a low barrier is expected compared to competing pathways that would lead to the β product.^12^, ^13^

**Scheme 3:**
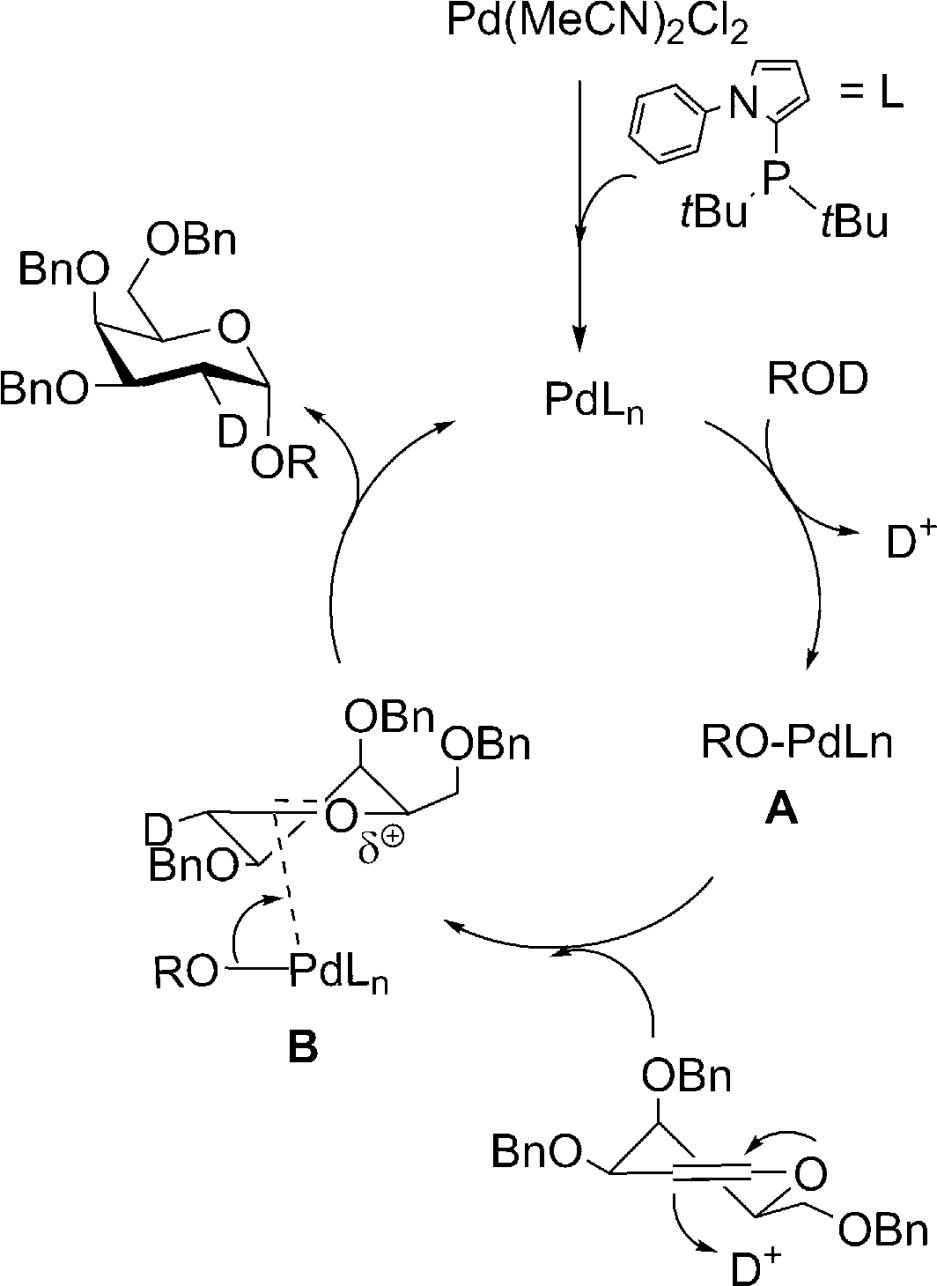
Proposed mechanism.

In conclusion, we have described the first example of a non‐π‐allyl‐mediated Pd‐catalyzed direct and stereoselective glycosylation of glycal enol ethers. This mechanistically interesting reaction is mild and widely applicable to a range of glycal donors and nucleophile acceptors, including some bearing alkene functionalities. The reaction proceeds with excellent yields and high selectivity for the α anomer and is tolerant of most common protecting groups. We have demonstrated the generality and versatility of the approach through the stereoselective synthesis of a series of disaccharides, glycosyl amino acids, and other glycoconjugates. Given the abundance of chiral acetals in natural products in which alkene functionalities are also featured, this method might find applications in and beyond the field of carbohydrates.

## Experimental Section

The glycal donor **1**, **4**, **5**, or **9** (ca. 50 mg, 1.0 equiv), nucleophile acceptor **2** (0.75 equiv), Pd(CH_3_CN)_2_Cl_2_ (0.25 equiv), and ligand **L** (0.3 equiv) were weighed into an oven‐dried microwave vial, sealed, and placed under vacuum for 1 h. The vial was then filled with N_2_ and approximately 1.0 mL anhydrous solvent (dichloromethane) was added. The mixtures were stirred and heated at 50 °C in the sealed vial until the reaction was determined to be complete by either TLC or NMR analysis of the crude material (See Tables 1 and 3 for specific details). The reaction mixture was quenched by filtering through a Celite bed and washed with additional solvent, then concentrated under reduced pressure and purified by column chromatography.

## Conflict of interest

The authors declare no conflict of interest.

## Supporting information

As a service to our authors and readers, this journal provides supporting information supplied by the authors. Such materials are peer reviewed and may be re‐organized for online delivery, but are not copy‐edited or typeset. Technical support issues arising from supporting information (other than missing files) should be addressed to the authors.

Supplementary
