# Supplementary material for: Palladium‐Catalyzed Direct Stereoselective Synthesis of Deoxyglycosides from Glycals
Source: Angew Chem Int Ed Engl. 2017 Feb 17;56(13):3640–4. doi: 10.1002/anie.201612071 (PMC5484376; doi:10.1002/anie.201612071)

Angewandte Chemie
Eine Zeitschrift der Gesellschaft Deutscher Chemiker

Supporting Information

Palladium-Catalyzed Direct Stereoselective Synthesis of Deoxyglycosides from Glycals

*Abhijit Sau, Ryan Williams, Carlos Palo-Nieto, Antonio Franconetti, Sandra Medina, and M. Carmen Galan**

anie_201612071_sm_miscellaneous_information.pdf

Table of Contents:

**General Experimental Procedures**	S3
**List of glycal donors used in the study**	S3
**List of nucleophile acceptors used in the study**	S4
**General Procedures for Glycosylation Reactions**	S4
**Acceptor Scope Results**	S4-S8
**Methyl 2,3,4-tri-*O*-benzyl-6-*O*-(2-deoxy-3,4,6-tri-*O*-benzyl-α-D-lyxo-hexapyranosyl)-α-D-glucopyranoside (3a)**	S4
**Phenylethyl 2-deoxy-3,4,6-tri-*O*-benzyl-α-D-lyxo-hexapyranoside (3b)**	S5
**Benzyl 2-deoxy-3,4,6-tri-*O*-benzyl-α-D-lyxo-hexapyranoside (3c)**	S5
**Methyl 2,3,4-tri-*O*-benzoyl-6-*O*-(2-deoxy-3,4,6-tri-*O*-benzyl-α-D-lyxo-hexapyranosyl)-α-D-glucopyranoside (3d)**	S6
**Phenyl 2,3,4-tri-*O*-benzoyl-6-*O*-(2-deoxy-3,4,6-tri-*O*-benzyl-α-D-lyxo-hexapyranosyl)-β-D-thioglucopyranoside (3e)**	S6
**Methyl 3-*O*-benzyl-2-*O*-(2-deoxy-3,4,6-tri-*O*-benzyl-α-D-lyxo-hexapyranosyl)-4,6-*O*-benzylidene-α-D-glucopyranoside (3f)**	S6
**3-*O*-(2-deoxy-3,4,6-Tri-*O*-benzyl-α-D-lyxo-hexapyranoside)-1,2:5,6-di-*O*-isopropylidene-α-D-glucofuranoside (3g)**	S7
**2-Deoxy-3,4,6-tri-*O*-benzyl-α-D-lyxo-hexopyranosyl-(1→*O*)-*N*-tert-butoxycarbonyl-L-serine methyl ester (3h)**	S7
***O*-Succinimido 2-deoxy-3,4,6-tri-*O*-benzyl-α-D-lyxo-hexapyranoside (3i)**	S8
**Cinnamyl 2-deoxy-3,4,6-tri-O-benzyl-α-D-lyxo-hexapyranoside (3j)**	S8
**Glycal Donor Scope Results**	S9-S14

**Methyl 2,3,4-tri-*O*-benzyl-6-*O*-(6-*O*-acetyl-2-deoxy-3,4-di-*O*-benzyl-α-D-lyxo-hexapyranosyl)-*α*-D-glucopyranoside (6b)**	S9
**Methyl 2,3,4-tri-*O*-benzyl-6-*O*-(2-deoxy-3,4,6-Tri-*O*-*tert*-butyldimethylsilyl-α-D-lyxo-hexapyranosyl)-α-D-glucopyranoside (6c)**	S9
**Methyl 2,3,4-tri-*O*-benzyl-6-*O*-(2-deoxy-3,4,6-tri-*O*-methyl-α-D-lyxo-hexapyranosyl)-α-D-glucopyranoside (6d)**	S10
**Methyl 2,3,4-tri-*O*-benzyl-6-*O*-(2-deoxy-3,4,6-tri-*O*-methoxymethylether-α-D-lyxo-hexapyranosyl)-α-D-glucopyranoside (6f)**	S11
**Methyl 2,3,4-tri-*O*-benzyl-6-*O*-(2-deoxy-3,4,6-tri-*O*-allyl-α-D-lyxo-hexapyranosyl)-α-D-glucopyranoside (6g)**	S11
**Methyl 2,3,4-tri-*O*-benzyl-6-*O*-(2-deoxy-3,4-*O*-(1,1,3,3-tetraisopropyldisiloxane-1,3-diyl)-6-*O*- benzyl -α-D-*erythro*-hexapyranosyl)-α-D-glucopyranoside (7a)**	S12
**Methyl 2,3,4-tri-*O*-benzyl-6-*O*-(2-deoxy-3,4-*O*-(1,1,3,3-tetraisopropyldisiloxane-1,3-diyl)-6-*O*-triisopropylsilyl-α-D-*erythro*-hexapyranosyl)-α-D-glucopyranoside (7b)**	S13
**Methyl 3-*O*-benzyl-2-*O*-(2-deoxy-3,4-*O*-(1,1,3,3-tetraisopropyldisiloxane-1,3-diyl)-6-*O*-triisopropylsilyl-α-D-erythro-hexapyranosyl)-4,6-*O*-benzylidene-α-D-glucopyranoside (7f)**	S13
**Methyl 2,3,4-tri-*O*-benzyl-6-*O*-(2,6-deoxy-3,4-*O*-(1,1,3,3-tetraisopropyldisiloxane-1,3-diyl)-α/β-L-*erythro*-hexapyranosyl)-α-D-glucopyranoside (8)**	S13
**Methyl 2-deoxy-3,4,6-tri-*O*-benzyl-α-D-lyxo-hexapyranoside (12)**	S14
**Glycosylation reactions in the presence of base as an acid scavenger:**	S14
**Experiments with Deuterated Substrates:**	S15-S16
**Methyl 2,3,4-tri-*O*-benzyl-6-*O*-(2-deoxy-3,4,6-tri-*O*-benzyl-2-^{2}H -α-D-lyxo-hexapyranosyl)-α-D-glucopyranoside (10)**	S14
**d_3-Methyl 2-deoxy-3,4,6-tri-*O*-benzyl-2-^{2}H-α-D-lyxo-hexapyranoside (11)**	S15
**In Situ Anomerization Experiment**	S17
**^{1}H-NMR experiments**	S19-S23
**References**	S24
**NMR Spectra**	S24-S90

General Experimental Procedures:

General. Chemicals were purchased and used without further purification. Dry solvents were obtained by distillation using standard procedures or by passage through a column of anhydrous alumina using equipment from Anhydrous Engineering (University of Bristol) based on the Grubbs' design. Reactions requiring anhydrous conditions were performed under nitrogen; glassware and needles were either flame dried immediately prior to use or placed in an oven (150 °C) for at least 2 hours and allowed to cool either in a desiccators or under reduced pressure; liquid reagents, solutions or solvents were added *via* syringe through rubber septa; solid reagents were added *via* Schlenk type adapters. Teflon rings were used between the joints of the condensers and round bottom flasks. Reactions were monitored by TLC on Kieselgel 60 F254 (Merck). Detection was by examination under UV light (254 nm) and by charring with 10% sulfuric acid in ethanol. Flash column chromatography was performed using silica gel [Merck, 230−400 mesh (40−63 μm)]. Extracts were concentrated *in vacuo* using both a Büchi rotary evaporator (bath temperatures up to 40 °C) at a pressure of either 15 mmHg (diaphragm pump) or 0.1 mmHg (oil pump), as appropriate, and a high vacuum line at room temperature. ^{1}H NMR and ^{13}C NMR spectra were measured in the solvent stated at 400 or 500 MHz. Chemical shifts are quoted in parts per million from residual solvent peak ($CDCl_3$: ^{1}H - 7.26 ppm and ^{13}C - 77.16 ppm) and coupling constants (*J*) given in Hertz. Multiplicities are abbreviated as: b (broad), s (singlet), d (doublet), t (triplet), q (quartet), m (multiplet) or combinations thereof. The units of the specific rotation, (deg·mL)/(g·dm), are implicit and are not included with the reported value. Concentration *c* is given in g/100 mL. Positive ion Matrix Assisted Laser Desorption Ionization Time-Of-Flight (MALDI-TOF) mass spectra were recorded using an HP-MALDI instrument using gentisic acid matrix. Electrospray ionisation (ESI) mass spectra were recorded on a Micromass LCT mass spectrometer or a VG Quattro mass spectrometer.

List of glycal donors used in the study:

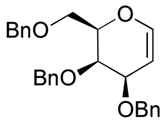

1a

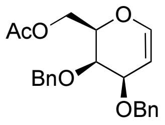

1b

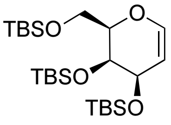

1c

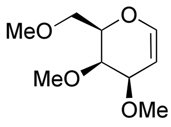

1d

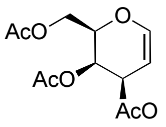

1e

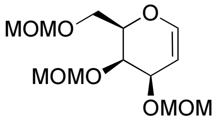

1f

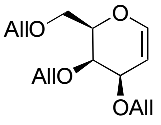

1g

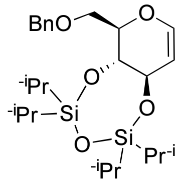

4a

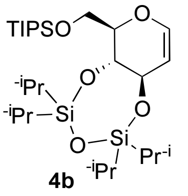

4b

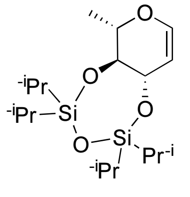

5

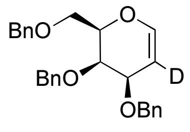

9

List of nucleophile acceptors used in the study:

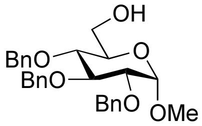

2a

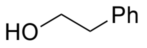

2b

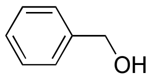

2c

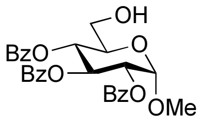

2d

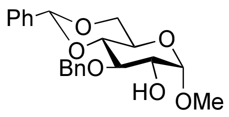

2e

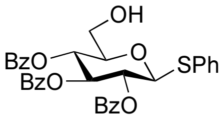

2f

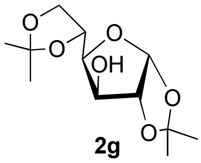

2g

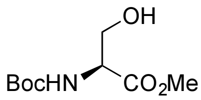

2h

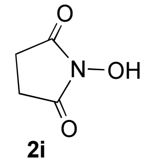

2i

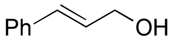

2j

General Glycosylation Procedure:

The glycal donor **1**, **4**, **5** or **9** (1.0 eq.), nucleophile acceptor **2** (0.75 eq.), $Pd(CH_3CN)_2Cl_2$ (0.25 eq.) and ligand **L** (0.3 eq.) were weighed into an oven dried microwave vial, sealed and placed under vacuum for 1 h. The vial was then filled with N_2 and ~ 1.0 ml anhydrous solvent (dichloromethane) was added. The mixtures were stirred and heated at 50 °C in the sealed vial until the reaction was determined to be complete by either TLC or NMR analysis of the crude material (Table 1 and 3 in manuscript for specific details). The reaction mixture was quenched by filtering through a Celite bed and washed with additional solvent then concentrated *in vacuo* and purified by column chromatography.

Acceptor Scope:

Methyl 2,3,4-tri-*O*-benzyl-6-*O*-(2-deoxy-3,4,6-tri-*O*-benzyl-α-D-lyxo-hexapyranosyl)-α-D-glucopyranoside (3a)

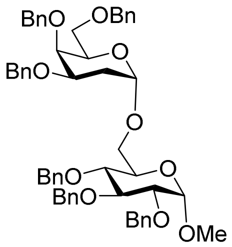

Following the *General Glycosylation Procedure*: galactal **2a** (50 mg, 0.120 mmol), acceptor **5a** (42 mg, 0.090 mmol), $Pd(CH_3CN)_2Cl_2$ (8 mg, 0.030 mmol) and ligand 2-(di-tert-butylphosphanyl)-1-phenyl-1H-pyrrole (10 mg, 0.036 mmol). Following purification by column chromatography (10:1 to 5:1, Hexane:EtOAc) product **3a** was obtained as an oil (71 mg, 90 %). The spectroscopic data was in agreement with previously reported data[1].

Phenylethyl 2-deoxy-3,4,6-tri-*O*-benzyl-α-D-lyxo-hexapyranoside (3b)

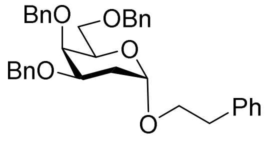

Following the *General Glycosylation Procedure*: glycosyl donor **1a** (100 mg, 0.240 mmol), phenethyl alcohol **2b** (22 mg, 0.180 mmol), $Pd(CH_3CN)_2Cl_2$ (16 mg, 0.060 mmol) and ligand 2-(di-tert-butylphosphanyl)-1-phenyl-1H-pyrrole (21 mg, 0.072 mmol) were used. Following purification by column chromatography (6:1, Hexane:EtOAc), the title compound was obtained as a pale yellow oil (67 mg, 69 %); ^{1}H NMR (500 MHz, $CDCl_3$) δ 7.40 – 7.14 (20H, m, Ph), 4.98 (1H, d, *J* = 3.5 Hz, H-1), 4.92 (1H, d, *J* = 11.6 Hz, OC*H*HPh), 4.61 (1H, d, *J* = 11.2 Hz, OCH*H*Ph), 4.60 (2H, s, OC*H*$_2$Ph), 4.47 (1H, d, *J* = 11.8 Hz, OC*H*HPh), 4.39 (1H, d, *J* = 11.8 Hz, OCH*H*Ph), 3.89 (1H, ddd, *J* = 12.0, 4.6, 2.5 Hz, H-3), 3.86 (1H, app s, H-4), 3.85 – 3.79 (1H, m, PhCH$_2$C*H*HO), 3.70 (1H, t, *J* = 6.5 Hz, H-5), 3.61 (1H, dt, *J* = 9.8, 6.7 Hz, PhCH$_2$CH*H*O), 3.56 (1H, dd, *J* = 9.4, 6.7 Hz, H-6a), 3.51 (1H, dd, *J* = 9.3 Hz, 6.1, H-6b), 2.86 (2H, t, *J* = 7.0, PhC*H*$_2$CH$_2$O), 2.21 (1H, td, *J* = 12.2, 3.7, H-2a), 1.98 (1H, app dd, *J* = 12.5, 4.4, H-2b); ^{13}C NMR (126 MHz, $CDCl_3$) δ 139.2 (4° C (phenethyl)), 139.1 (4° C), 138.7 (4° C), 138.3 (4° C), 129.1 (CH), 128.54 (CH), 128.48 (CH), 128.4 (CH), 128.33 (CH), 128.32 (CH), 127.8 (CH), 127.74 (CH), 127.66 (CH), 127.6 (CH), 127.5 (CH), 126.3 (CH), 97.8 (C-1), 74.8 (C-3), 74.4 (O*C*H$_2$Ph), 73.5 (O*C*H$_2$Ph), 73.2 (C-4), 70.5 (O*C*H$_2$Ph), 70.0 (C-5), 69.7 (C-6), 68.1 (PhCH$_2$*C*H$_2$O), 36.3 (Ph*C*H$_2$CH$_2$O), 31.3 (C-2); (ESI-HRMS) for $C_{35}H_{38}O_5Na^+$ (MNa^+) calculated: 561.2611; found: 561.2596; $[\alpha]_D^{23}$ + 63 [*c* 0.65, CH_2Cl_2].

Benzyl 2-deoxy-3,4,6-tri-*O*-benzyl-α-D-lyxo-hexapyranoside (3c)

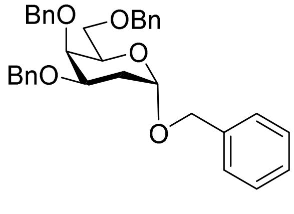

Following the *General Glycosylation Procedure*: glycosyl donor **1a** (100 mg, 0.240 mmol), benzyl alcohol **2c** (20 mg, 0.180 mmol), $Pd(CH_3CN)_2Cl_2$ (16 mg, 0.060 mmol) and ligand 2-(di-tert-butylphosphanyl)-1-phenyl-1H-pyrrole (21 mg, 0.072 mmol) were used. The reaction mixture was stirred for 17 h before being quenched. Following purification by column chromatography (9:1, Hexane:EtOAc) the title compound was obtained as a colourless oil (91 mg, 96 %). The spectroscopic data was in agreement with previously reported data[3].

Methyl 2,3,4-tri-*O*-benzoyl-6-*O*-(2-deoxy-3,4,6-tri-*O*-benzyl-α-D-lyxo-hexapyranosyl)-α-D-glucopyranoside (3d)

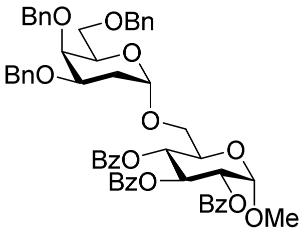

Following the general procedure, glycosyl donor **1a** (100 mg, 0.240 mmol), glycosyl acceptor **2d** (91 mg, 0.180 mmol), $Pd(CH_3CN)_2Cl_2$ (16 mg, 0.060 mmol) and ligand 2-(di-tert-butylphosphanyl)-1-phenyl-1H-pyrrole (21 mg, 0.072 mmol) were used. Following purification by column chromatography (6:1 to 4:1, Hexane:EtOAc) the title compound was obtained as a pale yellow oil (136 mg, 82 %). The spectroscopic data was in agreement with previously reported data[1].

Phenyl 2,3,4-tri-*O*-benzoyl-6-*O*-(2-deoxy-3,4,6-tri-*O*-benzyl-α-D-lyxo-hexapyranosyl)-β-D-thioglucopyranoside (3e)

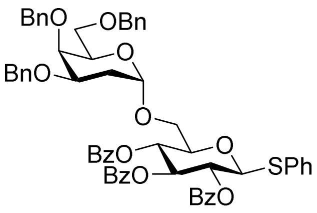

Following the *General Glycosylation Procedure*: glycosyl donor **1a** (50 mg, 0.120 mmol), glycosyl acceptor **2e** (53 mg, 0.090 mmol), $Pd(CH_3CN)_2Cl_2$ (8 mg, 0.030 mmol) and ligand 2-(di-tert-butylphosphanyl)-1-phenyl-1H-pyrrole (10 mg, 0.036 mmol) were used. Following purification by column chromatography (7:3, Hexane:EtOAc) the title compound was obtained as an oil (76 mg, 84 %). The spectroscopic data was in agreement with previously reported data[1].

Methyl 3-*O*-benzyl-2-*O*-(2-deoxy-3,4,6-tri-*O*-benzyl-α-D-lyxo-hexapyranosyl)-4,6-*O*-benzylidene-α-D-glucopyranoside (3f)

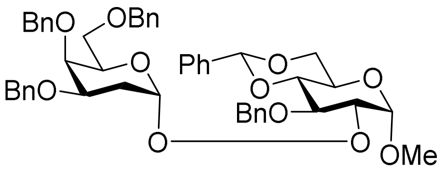

Following the *General Glycosylation Procedure*: galactal **1a** (50 mg, 0.120 mmol), acceptor **2f** (34 mg, 0.090 mmol), $Pd(CH_3CN)_2Cl_2$ (8 mg, 0.030 mmol) and ligand 2-(di-tert-butylphosphanyl)-1-phenyl-1H-pyrrole (10 mg, 0.036 mmol). Following purification by column chromatography (5:1 to 3:1, Hexane:EtOAc) product **3f** was obtained as an oil (53 mg, 73%). The spectroscopic data was in agreement with previously reported data[1].

3-*O*-(2-deoxy-3,4,6-Tri-*O*-benzyl-α-D-lyxo-hexapyranoside)-1,2:5,6-di-*O*-isopropylidene-α-D-glucofuranoside (3g)

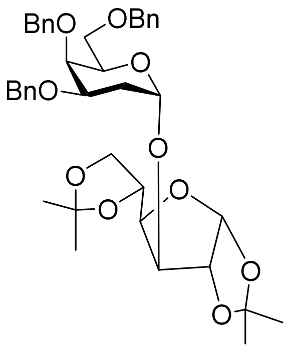

Following the *General Glycosylation Procedure*: galactal **1a** (50 mg, 0.120 mmol), acceptor **2g** (23 mg, 0.090 mmol), $Pd(CH_3CN)_2Cl_2$ (8 mg, 0.030 mmol) and ligand 2-(di-tert-butylphosphanyl)-1-phenyl-1H-pyrrole (10 mg, 0.036 mmol). Following purification by column chromatography (5:1 to 2:1, Hexane:EtOAc) product **3g** was obtained as an oil (45 mg, 74%). The spectroscopic data was in agreement with previously reported data[3].

2-Deoxy-3,4,6-tri-O-benzyl-α-D-lyxo-hexopyranosyl-(1→*O*)-*N*-tert-butoxycarbonyl-L-serine methyl ester (3h)

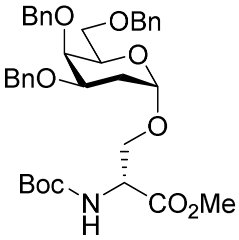

Following the *General Glycosylation Procedure*: glycosyl donor **1a** (100 mg, 0.240 mmol), Boc-L-serine methyl ester **2h** (39 mg, 0.180 mmol), $Pd(CH_3CN)_2Cl_2$ (16 mg, 0.060 mmol) and ligand 2-(di-tert-butylphosphanyl)-1-phenyl-1H-pyrrole (21 mg, 0.072 mmol) were used. Following purification by column chromatography (7:3, Hexane:EtOAc) the title compound was obtained as a colourless oil (101 mg, 88 %). The spectroscopic data was in agreement with previously reported data[4].

***O*-Succinimido 2-deoxy-3,4,6-tri-*O*-benzyl-α-D-lyxo-hexapyranoside (3i)**

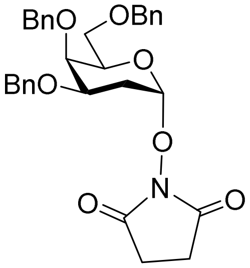

Following the *General Glycosylation Procedure*: galactal **1a** (50 mg, 0.120 mmol), N-hydroxy Succinimide (10 mg, 0.09 mmol) and Pd(CH_3CN)$_2$Cl$_2$ (8 mg, 0.030 mmol) and ligand 2-(di-tert-butylphosphanyl)-1-phenyl-1H-pyrrole **2i** (10 mg, 0.036 mmol). Following purification by column chromatography (5:1 to 1:1, Hexane:EtOAc) product **3i** was obtained as an oil (41 mg, 85 %): ^{1}H NMR (400 MHz; CDCl$_3$) ^{1}H NMR) δ 7.45 – 7.12 (15H, m, Ph), 5.58 (1H, t, *J* = 2.5 Hz, H-1), 4.93 (1H, d, *J* = 11.5 Hz, OC*H*HPh), 4.70 – 4.57 (4H, m, H-3, 3 OC*H*HPh), 4.57 – 4.38 (2H, m, OC*H*HPh), 4.12 – 3.97 (2H, m, H-4, H-5), 3.66 – 3.44 (2H, m, H-6a, H-6b), 2.63 (4H, q, *J* = 2.0 Hz, 2COCH$_2$), 2.43 – 2.23 (2H, m, H-2a, H-2b). ^{13}C NMR (101 MHz, CDCl$_3$) δ 171.2 (2C, *C*OCH$_3$), 138.7 (4° C), 138.3 (4° C), 138.2 (4° C), 128.4 (CH), 128.3 (CH), 128.2 (CH), 128.1 (CH), 127.7 (CH), 127.6 (CH), 127.6 (CH), 127.3 (CH), 102.6 (C-1), 74.4(C-5), 73.6 (*C*H$_2$Ph), 73.2 (*C*H$_2$Ph), 72.6 (C-4), 70.9 (*C*H$_2$Ph), 70.5.8 (C-3), 69.0 (C-5), 28.6 (C-2), 25.4 (2C, 2CO*C*H$_2$). ESI-HRMS for $C_{31}H_{33}NNaO_7^+$ (MNa^+) calculated: 554.2149; found: 554.2130. $[\alpha]_D^{21}$ + 98 [*c* 1.0, CHCl$_3$].

Cinnamyl 2-deoxy-3,4,6-tri-O-benzyl-α-D-lyxo-hexapyranoside (3j)

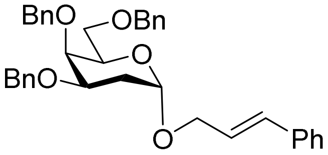

Following the general procedure, glycosyl donor **1a** (100 mg, 0.240 mmol), cinnamyl alcohol **2j** (24 mg, 0.180 mmol), bis(acetonitrile)dichloropalladium (II) (16 mg, 0.060 mmol) and ligand 2-(di-tert-butylphosphanyl)-1-phenyl-1H-pyrrole (0.021 g, 0.072 mmol) were used. Following purification by column chromatography (Hexane:EtOAc 11:1) the title compound was obtained as a solid (65 mg, 66 %). The spectroscopic data was in agreement with previously reported data[5].

Glycal Donor Scope:

Methyl 2,3,4-tri-*O*-benzyl-6-*O*-(6-*O*-acetyl-2-deoxy-3,4-di-*O*-benzyl-α-D-lyxo-hexapyranosyl)-*α*-D-glucopyranoside (6b)

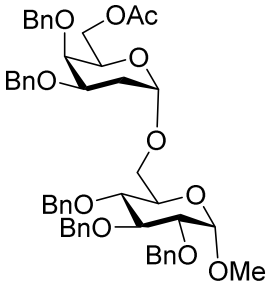

Following the *General Glycosylation Procedure*: galactal **1b** (50 mg, 0.136 mmol), acceptor **2a** (47 mg, 0.102 mmol), $Pd(CH_3CN)_2Cl_2$ (9 mg, 0.034 mmol) and ligand 2-(di-tert-butylphosphanyl)-1-phenyl-1H-pyrrole (12 mg, 0.407 mmol). Following purification by column chromatography (10:1 to 4:1, Hexane:EtOAc) product **6b** was obtained as an oil (68 mg, 81%): ^{1}H NMR (400 MHz; $CDCl_3$) δ 7.41 – 7.19 (25H, m, Ph), 5.03 (1H, d, *J* = 3.3 Hz, H-1'), 4.99 (1H, d, *J* = 10.7 Hz, OC*H*HPh), 4.94 (1H, d, *J* = 11.7 Hz, OC*H*HPh), 4.87 (1H, d, *J* = 11.0 Hz, OC*H*HPh), 4.83 – 4.77 (2H, m, OC*H*HPh), 4.72 – 4.63 (2H, m, OC*H*HPh), 4.63 – 4.55 (3H, m, H-1, 2H, OC*H*HPh), 4.48 (1H, d, *J* = 11.1 Hz, OC*H*HPh), 4.11 – 3.95 (3H, m, H-3, H-6a',H-6b'), 3.84 (1H, ddd, *J* = 11.9, 4.5, 2.3 Hz, H-3'), 3.74 (4H, tt, *J* = 9.7, 5.1 Hz, H-4', H-6a, H-2, H-5), 3.60 (1H, d, *J* = 10.5 Hz, H-6b), 3.51 (1H, dd, *J* = 9.6, 3.6 Hz, H-2), 3.43 (1H, t, *J* = 9.3 Hz, H-4), 3.31 (3H, s, OCH_3), 2.20 (1H, td, *J* = 12.3, 3.6 Hz, H-2a'), 2.03 (1H, dd, *J* = 12.5, 4.4 Hz, H-2b'), 1.85 (3H, s, CH_3CO). ^{13}C NMR (101 MHz, $CDCl_3$) δ 170.5 (CH_3*C*O) 138.6 (4° C), 138.4 (4° C), 138.2 (4° C), 128.5 (CH), 128.4 (CH), 128.4 (CH), 128.4 (CH), 128.3 (CH), 128.1 (CH), 127.9 (CH), 127.7 (CH), 127.7 (CH), 127.5 (CH), 127.4 (CH), 98.0 (C-1'), 97.8 (C-1), 82.1 (C-3), 79.9 (C-2), 77.9 (C-4), 75.8 (*C*H_2Ph), 74.9 (*C*H_2Ph), 74.2 (C-3') , 74.0 (*C*H_2Ph), 73.2 (*C*H_2Ph), 72.5 (C-5'), 70.3 (*C*H_2Ph), 69.7 (H-4'), 69.1(H-5), 65.9 (C-6), 64.0 (C-6'), 55.0 (OCH_3), 30.7 (C-2'), 20.8 (CO*C*H_3). ESI-HRMS for $C_{50}H_{56}NaO11^+$ (MNa^+) calculated: 855.3720; found: 855.3711. $[\alpha]_D^{25}$ + 8 [*c* 1.0, $CHCl_3$].

Methyl 2,3,4-tri-*O*-benzyl-6-*O*-(2-deoxy-3,4,6-Tri-*O*-*tert*-butyldimethylsilyl-α-D-lyxo-hexapyranosyl)-α-D-glucopyranoside (6c)

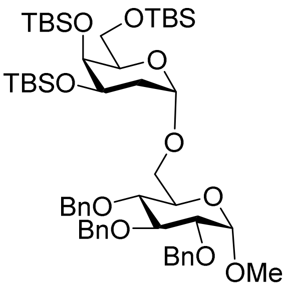

Following the *General Glycosylation Procedure*: galactal **1c** (50 mg, 0.102 mmol), acceptor **2a** (36 mg, 0.077 mmol), $Pd(CH_3CN)_2Cl_2$ (7 mg, 0.026 mmol) and ligand 2-(di-tert-butylphosphanyl)-1-phenyl-1H-pyrrole (9 mg,

0.030). Following purification by column chromatography (20:1 to 8:1, Hexane:EtOAc) product **6c** was obtained as an oil (60 mg, 82%): ^{1}H NMR (400 MHz, $CDCl_3$) δ: 7.38-7.24 (15H, m, Ph), 4.99-4.95 (2H, m, H-1', OC*H*HPh), 4.87 (1H, d, *J* = 10.8 Hz, OC*H*HPh), 4.83 (1H, d, *J* = 10.7 Hz, OCH*H*Ph), 4.80 (1H, d, *J* = 12.2 Hz, OC*H*HPh), 4.67 (1H, d, *J* = 12.2 Hz, OCH*H*Ph), 4.61 (1H, d, *J* = 10.8 Hz, OCH*H*Ph), 4.57 (1H, d, *J* = 3.6 Hz, H-1), 4.05 (1H, m, H-3'), 3.99 (1H, t, *J* = 9.2 Hz, H-2), 3.80 (1H, bs, H-4'), 3.77-3.73 (2H, m, H-3, H-6'a), 3.70-3.62 (4H, m, H-5', H-6a, H-6b, H-6'b), 3.49 (1H, dd, *J* = 9.6, 3.6 Hz, H-4), 3.42 (1H, t, *J* = 9.6, H-5), 3.36 (3H, s, OCH_3), 2.08 (1H, td, *J* = 12.1, 3.5, H-2a'), 1.65 (1H, dd, *J* = 12.7, 4.5, H-2b'), 0.94-0.86 (27H, m, 3 x $SiC(CH_3)_3$), 0.12-0.00 (18H, m, 6 x $SiCH_3$); ^{13}C NMR (126 MHz; $CDCl_3$) 138.7 (4° C), 138.2 (4° C), 138.1 (4° C), 128.4-127.7 (Ph), 97.9 (C-1'), 97.6 (C-1), 82.1 (C-2), 79.9 (C-4), 78.5 (C-5), 75.8 (O*C*H_2Ph), 75.1 (O*C*H_2Ph), 73.3 (O*C*H_2Ph), 72.7 (C-5'), 70.3 (C-4'), 68.3 (C-3'), 65.2 (C-6'), 62.6 (C-6), 54.7 (OCH_3), 33.5 (C-2'), 26.2 ($SiC(CH_3)_3$), 26.1 ($SiC(CH_3)_3$), 25.8 ($SiC(CH_3)_3$), 18.6 ($SiC(CH_3)_3$), 18.5 ($SiC(CH_3)_3$), 18.1 ($SiC(CH_3)_3$), -3.9 ($SiCH_3$), -4.4 ($SiCH_3$), -4.8 ($SiCH_3$), -5.0 ($SiCH_3$), -5.3 ($SiCH_3$), -5.4 ($SiCH_3$). ESI-HRMS for $C_{52}H_{84}NaSi_3O_{10}^+$ (MNa^+) calculated: 975.5270; found: 975.5277. $[\alpha]_D^{25}$ + 18 [*c* 1.0, $CHCl_3$].

Methyl 2,3,4-tri-*O*-benzyl-6-*O*-(2-deoxy-3,4,6-tri-*O*-methyl-α-D-lyxo-hexapyranosyl)-α-D-glucopyranoside (6d)

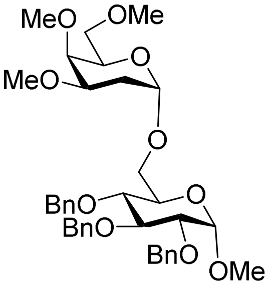

Following the *General Glycosylation Procedure*: galactal **1d** (50 mg, 0.266 mmol), acceptor **2a** (93 mg, 0.199mmol), $Pd(CH_3CN)_2Cl_2$ (17 mg, 0.067 mmol.) and ligand 2-(di-tert-butylphosphanyl)-1-phenyl-1H-pyrrole (23 mg, 0.0797 mmol). Following purification by column chromatography (10:1 to 5:1, Hexane:EtOAc) product **6d** was obtained as an oil (100 mg, 78%): ^{1}H NMR (400 MHz; $CDCl_3$) δ 7.41 – 7.22 (15H, m, Ph), 5.02 – 4.96 (2H, m, H-1', OC*H*HPh), 4.92 (1H, d, *J* = 10.9 Hz, OC*H*HPh), 4.83 – 4.77 (2H, m, 2 OC*H*HPh), 4.68 (1H, d, *J* = 12.2 Hz, OC*H*HPh), 4.62 (1H, d, *J* = 4.6 Hz, OC*H*HPh), 4.60 (1H, d, *J* = 2.9 Hz, H-1), 4.01 (1H, t, *J* = 9.2 Hz, H-3), 3.88 – 3.79 (2H, m, H-4, 6a'), 3.74 (1H, ddd, *J* = 9.9, 4.4, 1.8 Hz, H-5), 3.63 (1H, dd, *J* = 11.4, 2.0 Hz, H-6b'), 3.61 – 3.56 (2H, m, H-3',H-5'), 3.56 – 3.51 (5H, m, H-4', OCH_3, H-2), 3.51 – 3.40 (2H, m, H-6a, H-6b), 3.38 (6H, d, *J* = 8.2 Hz, 2 OCH_3), 3.30 (3H, s, OCH_3), 2.04 – 1.90 (2H, m, H-2a',H-2b'). ^{13}C NMR (101 MHz, $CDCl_3$) δ 138.6 (4° C), 138.2 (4° C), 138.1 (4° C), 128.5 (CH), 128.4 (CH), 128.4 (CH), 128.1 (CH), 128.1 (CH), 127.9 (CH), 127.7 (CH), 98.2 (C-1'), 97.9 (C-1), 82.1 (C-3), 80.0 (C-2), 77.8 (C-1), 75.9, 75.8, 74.9 (C-3', C-4', C-5'), 74.4 (*C*H_2Ph), 73.3 (*C*H_2Ph), 71.5 (*C*H_2Ph), 69.7 (C-6), 69.7 (C-5), 66.1 (C-4), 60.9 (OCH_3), 59.1 (OCH_3), 56.1 (OCH_3), 55.1 (OCH_3), 30.7 (C-2'). ESI-HRMS for $C_{37}H_{48}NaO_{10}$ (MNa+) calculated: 675.3145; found: 675.3144. $[\alpha]_D^{25}$ + 51 [*c* 1.0, $CHCl_3$].

Methyl 2,3,4-tri-*O*-benzyl-6-*O*-(2-deoxy-3,4,6-tri-*O*-methoxymethylether-α-D-lyxo-hexapyranosyl)-α-D-glucopyranoside (6f)

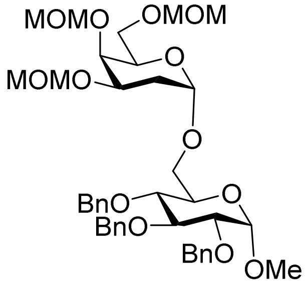

Following the *General Glycosylation Procedure*: galactal **1f** (50 mg, 0.179 mmol), acceptor **2a** (63 mg, 0.135 mmol) and $Pd(CH_3CN)_2Cl_2$ (12 mg, 0.045 mmol), ligand 2-(di-tert-butylphosphanyl)-1-phenyl-1H-pyrrole (15 mg, 0.0539 mmol). Following purification by column chromatography (7:1 to 2:1, Hexane:EtOAc) product **6f** was obtained as an oil (85 mg, 85%): ^{1}H NMR (500 MHz; $CDCl_3$) δ 7.38 – 7.24 (15H, m, Ar-H), 5.04 (1H, d, *J* = 3.1 Hz, H-1'), 4.98 (1H, d, *J* = 10.7 Hz, OC*H*HPh,), 4.92 (1H, d, *J* = 1.3 Hz, OC*H*HO), 4.90 (1H, d, *J* = 5.4 Hz, OC*H*HPh), 4.81 (1H, d, *J* = 2.8 Hz, OC*H*HPh), 4.79 (1H, d, *J* = 4.1 Hz, OC*H*HPh), 4.70 (1H, d, *J* = 6.8 Hz, OC*H*HO), 4.68 (1H, d, *J* = 1.1 Hz, OC*H*HO), 4.66 (1H, d, *J* = 4.2 Hz, OC*H*HPh), 4.63 (1H, d, *J* = 6.2 Hz, OC*H*HPh), 4.61 (1H, d, *J* = 2.0 Hz, OC*H*HO), 4.60 (1H, d, *J* = 3.6 Hz, H-1), 4.54 (2H, s, OCH_2O), 4.05 – 4.00 (1H, m, H-3'), 4.00 – 3.97 (1H, m, H-3), 3.91 – 3.87 (2H, m, H-4', H-5'), 3.84 (1H, dd, *J* = 11.4, 4.7 Hz, H-6a), 3.75 (1H, ddd, *J* = 10.1, 4.7, 1.9 Hz, H-5), 3.68 – 3.62 (3H, m, H-6b, H-6a', H-6b'), 3.55 – 3.49 (2H, m, H-4, H-2), 3.41 (3H, s, OCH_3), 3.37 (3H, s, OCH_3), 3.35 (3H, s, OCH_3), 3.27 (3H, s, OCH_3), 2.12 – 2.04 (1H, m, H-2a'), 1.93 (1H, ddt, *J* = 12.8, 4.8, 1.2 Hz, H-2b'). ^{13}C NMR (101 MHz, $CDCl_3$) δ 138.7 (4° C), 138.4 (4° C), 138.2 (4° C), 128.5 (CH), 128.4 (CH), 128.4 (CH), 128.1 (CH), 128.0 (CH), 127.8 (CH), 127.7 (CH), 127.6 (CH), 98.0 (C-1'), 97.9 (C-1), 97.37 (OCH_2), 96.7 (OCH_2), 94.3 (OCH_2), 82.2 (C-3), 80.0 (C-4), 77.9 (C-2), 75.8 (CH_2Ph), 74.9 (CH_2Ph), 73.4 (CH_2Ph), 71.8 (C-4'), 70.9 (C-3'), 69.9b (C-5'), 69.8 (C-5), 67.4(C-6'), 66.1 (C-6), 56.1 (OCH_3), 55.5 (OCH_3), 55.3 (OCH_3), 55.0 (OCH_3), 31.3 (C-2'). ESI-HRMS for $C_{40}H_{54}NaO_{13}^+$ (MNa^+) calculated: 765.3462; found: 765.3453. $[\alpha]_D^{25}$ + 28 [*c* 1.0, $CHCl_3$].

Methyl 2,3,4-tri-*O*-benzyl-6-*O*-(2-deoxy-3,4,6-tri-*O*-allyl-α-D-lyxo-hexapyranosyl)-α-D-glucopyranoside (6g)

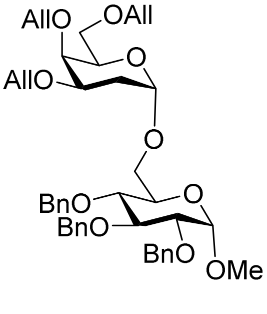

Following the *General Glycosylation Procedure*: galactal **1g** (50 mg, 0.188 mmol), acceptor **2a** (65 mg, 0.141 mmol), $Pd(CH_3CN)_2Cl_2$ (12 mg, 0.047 mmol) and ligand 2-(di-tert-butylphosphanyl)-1-phenyl-1H-pyrrole (16 mg, 0.056 mmol). Following purification by column chromatography (7:1 to 3:1, Hexane:EtOAc) product **6g** was

obtained as an oil (69 mg, 68%): ^{1}H NMR (500 MHz; $CDCl_3$) δ 7.41 – 7.22 (m, 15H, Ar-H), 6.00 – 5.79 (m, 3H, C*H*=CH₂), 5.30 (dt, *J* = 3.4, 1.6 Hz, 1H, CH=C*H*₂), 5.26 (q, *J* = 1.8 Hz, 1H, CH=C*H*₂), 5.23 (tt, *J* = 2.3, 1.1 Hz, 1H, CH=C*H*₂), 5.17 (dt, *J* = 3.4, 1.6 Hz, 1H, CH=C*H*₂), 5.15 – 5.12 (m, 2H, CH=C*H*₂), 5.01 (d, *J* = 2.6 Hz, 1H, H-1'), 4.99 (d, *J* = 10.8 Hz, 1H, OC*H*HPh), 4.91 (d, *J* = 10.8 Hz, 1H, OC*H*HPh), 4.82 (d, *J* = 5.3 Hz, 1H, OC*H*HPh), 4.80 (d, *J* = 6.6 Hz, 1H, OC*H*HPh), 4.69 (d, *J* = 12.2 Hz, 1H, OC*H*HPh), 4.62 (d, *J* = 2.5 Hz, 1H, H-1), 4.61 (d, *J* = 4.8 Hz, 1H, OC*H*HPh), 4.35 (ddt, *J* = 12.7, 5.6, 1.4 Hz, 1H, OC*H*HCH=CH₂), 4.13 – 4.06 (m, 1H, OC*H*HCH=CH₂), 4.04 (ddt, *J* = 8.5, 5.4, 1.6 Hz, 3H, 2H-OC*H*HCH=CH₂, H-3), 4.01 – 3.97 (m, 1H, OC*H*HCH=CH₂), 3.94 (ddt, *J* = 9.8, 5.6, 1.4 Hz, 1H, OC*H*HCH=CH₂), 3.91 (dt, *J* = 5.6, 1.4 Hz, 1H, OC*H*HCH=CH₂), 3.88 – 3.81 (m, 2H, H-4, H-6a'), 3.78 – 3.73 (m, 3H, H-4', H-3', H-5), 3.64 (dd, *J* = 11.4, 2.0 Hz, 1H, H-6b'), 3.58 (dd, *J* = 9.3, 7.3 Hz, 1H, H-5'), 3.56 – 3.52 (m, 1H, H-2), 3.51 – 3.46 (m, 2H, H-6a, H-6b), 3.37 (s, 3H, OCH_3), 2.14 – 2.07 (m, 1H, H-2a'), 1.95 – 1.90 (m, 1H, H-2b').^{13}C NMR (101 MHz, $CDCl_3$) δ 138.7 (4° C), 138.2 (4° C), 138.1 (4° C), 135.6 (*C*H=CH₂), 134.8 (*C*H=CH₂), 134.6 (*C*H=CH₂), 128.5 (CH), 128.4 (CH), 128.04 (CH), 127.9 (CH), 127.7 (CH), 127.7 (CH), 116.9 (CH=*C*H₂), 116.8 (CH=*C*H₂), 116.6 (CH=*C*H₂), 98.2 (C-1'), 97.9 (C-1), 82.1 (C-3), 80.0 (C-5'), 77.9 (C-2), 75.8 (*C*H₂Ph), 74.9 (*C*H₂Ph), 73.9 (C-4'), 73.5 (O*C*H₂CH), 73.3 (*C*H₂Ph), 72.4 (H-5), 72.2 (O*C*H₂CH) , 69.8 (2C, C-4, C-3'), 69.2 (O*C*H₂CH), 69.1 (C-6), 66.0 (C-6'), 55.0 (OCH_3), 31.1 (C-2'). ESI-HRMS for $C_{43}H_{54}NaO_{10}^{+}$ (MNa^{+}) calculated: 753.3615; found: 753.3610. . $[\alpha]_D^{25}$ + 20 [*c* 1.0, $CHCl_3$].

Methyl 2,3,4-tri-*O*-benzyl-6-*O*-(2-deoxy-3,4-*O*-(1,1,3,3-tetraisopropyldisiloxane-1,3-diyl)-6-*O*- benzyl -α-D-*erythro*-hexapyranosyl)-α-D-glucopyranoside (7a)

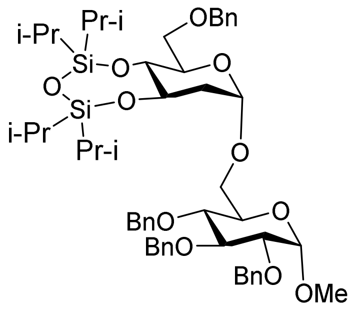

Following the *General Glycosylation Procedure*: glucal **4a** (50 mg, 0.104 mmol), acceptor **2a** (36 mg, 0.078 mmol) and $Pd(CH_3CN)_2Cl_2$ (7 mg, 0.026mmol) and ligand 2-(di-tert-butylphosphanyl)-1-phenyl-1H-pyrrole (9 mg, 0.031 mmol). Following purification by column chromatography (Hexane:EtOAc, 9:1 to 8:2) afforded the product **7a** as a syrup (63 mg, 86%). The spectroscopic data was in agreement with previously reported data[2].

Methyl 2,3,4-tri-*O*-benzyl-6-*O*-(2-deoxy-3,4-*O*-(1,1,3,3-tetraisopropyldisiloxane-1,3-diyl)-6-*O*-triisopropylsilyl-α-D-*erythro*-hexapyranosyl)-α-D-glucopyranoside (7b)

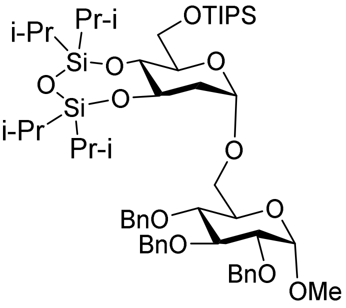

Following the *General Glycosylation Procedure*: galactal **4b** (50 mg, 0.092 mmol), acceptor **2a** (32 mg, 0.069 mmol), $Pd(CH_3CN)_2Cl_2$ (7 mg, 0.023 mmol) and ligand 2-(di-tert-butylphosphanyl)-1-phenyl-1H-pyrrole (8 mg, 0.028 mmol). Following purification by column chromatography (Hexane:EtOAc, 25:1 to 12:1) afforded the product **7c** as a syrup (52 mg, 75%). The spectroscopic data was in agreement with previously reported data[2].

Methyl 3-*O*-benzyl-2-*O*-(2-deoxy-3,4-*O*-(1,1,3,3-tetraisopropyldisiloxane-1,3-diyl)-6-*O*-triisopropylsilyl-α-D-erythro-hexapyranosyl)-4,6-*O*-benzylidene-α-D-glucopyranoside (7f)

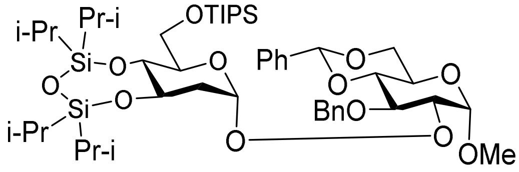

Following the *General Glycosylation Procedure*: galactal **4b** (50 mg, 0.92 mmol), acceptor **2f** (26 mg, 0.069 mmol), $Pd(CH_3CN)_2Cl_2$ (6 mg, 0.023 mmol) and ligand 2-(di-tert-butylphosphanyl)-1-phenyl-1H-pyrrole (8 mg, 0.028 mmol). Following purification by column chromatography (Hexane:EtOAc, 25:1 to 12:1) afforded the product **7g** as a syrup (43 mg, 68%). The spectroscopic data was in agreement with previously reported data[2].

Methyl 2,3,4-tri-*O*-benzyl-6-*O*-(2,6-deoxy-3,4-*O*-(1,1,3,3-tetraisopropyldisiloxane-1,3-diyl)-α/β-L-*erythro*-hexapyranosyl)-α-D-glucopyranoside (8)

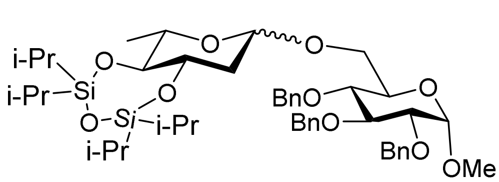

Following the *General Glycosylation Procedure*: rhamnal **5** (50 mg, 0.134 mmol), acceptor **2a** (47 mg, 0.100 mmol), $Pd(CH_3CN)_2Cl_2$ (9 mg, 0.034 mmol) and ligand 2-(di-tert-butylphosphanyl)-1-phenyl-1H-pyrrole (12 mg,

0.040 mmol). Following purification by column chromatography (Hexane:EtOAc, 25:1 to 10:1) afforded the product **8** as a syrup (62 mg, 75%, β:α = 1:10). The spectroscopic data was in agreement with previously reported data[2].

Methyl 2-deoxy-3,4,6-tri-*O*-benzyl-α-D-lyxo-hexapyranoside (12)

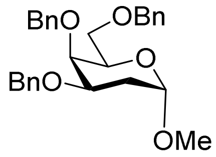

Following the *General Glycosylation Procedure*: galactal **1a** (50 mg, 0.120 mmol), CH_3OH (6 mg, 0.19 mmol) and $Pd(CH_3CN)_2Cl_2$ (8 mg, 0.030 mmol) and ligand 2-(di-tert-butylphosphanyl)-1-phenyl-1H-pyrrole (10 mg, 0.036 mmol). Following purification by column chromatography (10:1 to 5:1, Hexane:EtOAc) product **12** was obtained as an oil (48 mg, 88 %): ^{1}H NMR (400 MHz; $CDCl_3$) ^{1}H NMR δ 7.48 – 7.09 (m, 15H, Ph), 4.94 (d, J = 11.6 Hz, 1H, OC*H*HPh), 4.88 (d, J = 2.9 Hz, 1H, H-1), 4.63 (d, J = 11.7 Hz, 1H, OC*H*HPh), 4.60 (s, 2H, OC*H*HPh), 4.52 (d, J = 11.8 Hz, 1H, OC*H*HPh), 4.44 (d, J = 11.8 Hz, 1H, OC*H*HPh), 3.95 – 3.86 (m, 3H, H-3, H-4, H-5), 3.60 (dd, J = 6.4, 1.8 Hz, 2H, H-6a, H-6b), 3.33 (s, 3H, OCH_3), 2.22 (dd, J = 12.4, 3.7 Hz, 1H, H-2ax) 2.00 (ddt, J = 12.7, 4.5, 1.4 Hz, 1H, H-2eq).^{13}C NMR (101 MHz, $CDCl_3$) δ 138.9 (4° C), 138.6 (4° C), 138.12 (4° C), 128.4 (CH), 128.4 (CH), 128.2 (CH), 128.2 (CH), 127.8 (CH), 127.7 (CH), 127.5 (CH), 127.3 (CH), 98.9 (C-1), 74.7(C-5), 74.3 (CH_2Ph), 73.5 (CH_2Ph), 73.0 (C-4), 70.4 (CH_2Ph), 69.8 (C-3), 69.7 (C-5), 54.8 (OCH_3), 31.1 (C-2). The spectroscopic data was in agreement with previously reported data[3].

Glycosylation reactions in the presence of base as an acid scavenger:

Following the *General Glycosylation Procedure*: galactal **2a** (50 mg, 0.120 mmol), acceptor **5a** (42 mg, 0.090 mmol), $Pd(CH_3CN)_2Cl_2$ (8 mg, 0.030 mmol), ligand 2-(di-tert-butylphosphanyl)-1-phenyl-1H-pyrrole (10 mg, 0.036 mmol) and either K_2CO_3 (4.5 mg, 0.030 mmol), or 1-phenylpyrrole (4.3 mg, 0.030 mmol. No reaction was observed and only starting materials were recovered.

Experiments with Deuterated Substrates:

Glycosylation with deuterated galactal donor:

Methyl 2,3,4-tri-*O*-benzyl-6-*O*-(2-deoxy-3,4,6-tri-*O*-benzyl-2-^{2}H -α-D-lyxo-hexapyranosyl)-α-D-glucopyranoside (10)

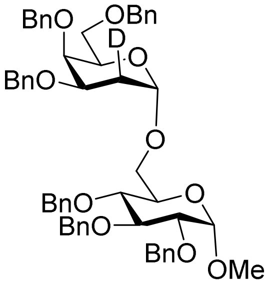

Following the *General Glycosylation Procedure*: galactal **9** (50 mg, 0.120 mmol), acceptor **2a** (42 mg, 0.090 mmol) and Pd(CH_3CN)$_2$$Cl_2$ (8 mg, 0.030 mmol) and ligand 2-(di-tert-butylphosphanyl)-1-phenyl-1H-pyrrole (10 mg, 0.036 mmol). Following purification by column chromatography (10:1 to 5:1, Hexane:EtOAc) product **10** was obtained as an oil (71 mg, 90 %): ^{1}H NMR (500 MHz; $CDCl_3$) δ 7.40 – 7.21 (30H, m, Ar-H), 5.04 (1H, d, *J* = 1.3 Hz, H-1'), 5.00 (1H, d, *J* = 10.8 Hz, OC*H*HPh), 4.93 (1H, d, *J* = 11.6 Hz, OC*H*HPh), 4.86 (1H, d, *J* = 10.9 Hz, OC*H*HPh), 4.83 – 4.78 (2H, m, OC*H*HPh), 4.69 (1H, d, *J* = 12.2 Hz, OC*H*HPh), 4.62 (1H, d, *J* = 2.7 Hz, H-1), 4.61 (1H, d, *J* = 5.4 Hz, OC*H*HPh), 4.58 (2H, s, OC*H*HPh), 4.54 (1H, d, *J* = 10.8 Hz, OC*H*HPh), 4.42 (1H, d, *J* = 11.8 Hz, OC*H*HPh), 4.35 (1H, d, *J* = 11.8 Hz, OC*H*HPh), 4.04 – 3.96 (1H, t, *J* = 9.3 Hz H-3), 3.89 (3H, t, *J* = 3.1 Hz, H-3', H-4', H-5'), 3.83 (1H, dd, *J* = 11.4, 4.7 Hz, H-6a), 3.74 (1H, ddd, *J* = 10.0, 4.6, 1.8 Hz, H-5), 3.63 (1H, dd, *J* = 11.4, 1.9 Hz, H-6b), 3.58 – 3.54 (2H, m, H-6a, H-2), 3.54 – 3.51 (1H, m, H-6b), 3.48 (1H, dd, *J* = 10.1, 8.9 Hz, H-4), 3.33 (3H, s, OCH_3), 2.03 – 1.99 (1H, m, H-2'). ^{13}C NMR (101 MHz, $CDCl_3$) δ 138.9 (4° C), 138.7 (4° C), 138.4 (4° C), 138.3 (4° C), 138.16 (4° C), 138.1 (4° C), 128.41 (CH), 128.2 (CH), 127.7 (CH), 98.3 (C-1'), 97.9 (C-1), 82.1 (C-3), 80.0 (C-2), 77.9 (C-4), 75.8 (*C*H_2Ph), 74.9 (*C*H_2Ph), 74.3 (*C*H_2Ph), 74.16 (C-3'), 73.3 (*C*H_2Ph), 73.3 (*C*H_2Ph), 72.9 (C-4'), 70.2 (*C*H_2Ph), 70.1 (*C*H_2Ph), (C-5'), 69.8 (C-5), 69.4 (C-6'), 66.0 (C-6), 55.0 (OCH_3), 29.7 (C-2'). ESI-HRMS for $C_{55}H_{59}DNaO_{10}^+$ (MNa^+) calculated: 904.4147; found: 904.4142. $[\alpha]_D^{25}$ + 25 [*c* 1.0, $CHCl_3$].

Glycosylation with CD_3OD as the nucleophile acceptor:

d$_3$-Methyl 2-deoxy-3,4,6-tri-*O*-benzyl-2-^{2}H-α-D-lyxo-hexapyranoside (11)

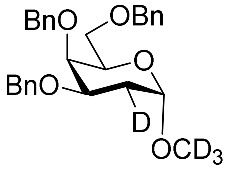

Following the *General Glycosylation Procedure*: galactal **1a** (50 mg, 0.120 mmol), CD_3OD (6 mg, 0.17 mmol) and Pd(CH_3CN)$_2$$Cl_2$ (8 mg, 0.030 mmol) and ligand 2-(di-tert-butylphosphanyl)-1-phenyl-1H-pyrrole (10 mg, 0.036 mmol). Following purification by column chromatography (10:1 to 5:1, Hexane:EtOAc) product **11** was

obtained as an oil (49 mg, 90 %): ^{1}H NMR (400 MHz; $CDCl_3$) ^{1}H NMR δ 7.39 – 7.22 (m, 15H, Ph), 4.94 (1H, d, J = 11.6 Hz, OC*H*HPh), 4.88 (1H, d, J = 3.7 Hz, H-1), 4.63 (1H, d, J = 11.7 Hz, OC*H*HPh), 4.60 (2H, s, OC*H*HPh), 4.53 (1H, d, J = 11.8 Hz, OC*H*HPh), 4.44 (1H, d, J = 11.8 Hz, OC*H*HPh), 3.95 – 3.86 (3H, m, H-3, H-4, H-5), 3.60 (2H, dd, J = 6.4, 1.8 Hz, H-6a, H-6b), 2.22 (1H, dd, J = 12.4, 3.7 Hz, H-2ax).^{13}C NMR (101 MHz, $CDCl_3$) δ 138.9 (4° C), 138.6 (4° C), 138.1 (4° C), 128.4 (CH), 128.4 (CH), 128.2 (CH), 128.2 (CH), 127.8 (CH), 127.7 (CH), 127.5 (CH), 127.3 (CH), 98.9 (C-1), 74.7(C-5), 74.3 (CH_2Ph), 73.5 (CH_2Ph), 72.9 (C-4), 70.4 (CH_2Ph), 69.8 (C-3), 69.7 (C-5, 29.7 (C-2). ESI-HRMS for $C_{28}H_{28}D4NaO_5^+$ (MNa^+) calculated: 475.2399; found: 475.2389. $[\alpha]_D^{25}$ + 39 [c 1.0, $CHCl_3$].

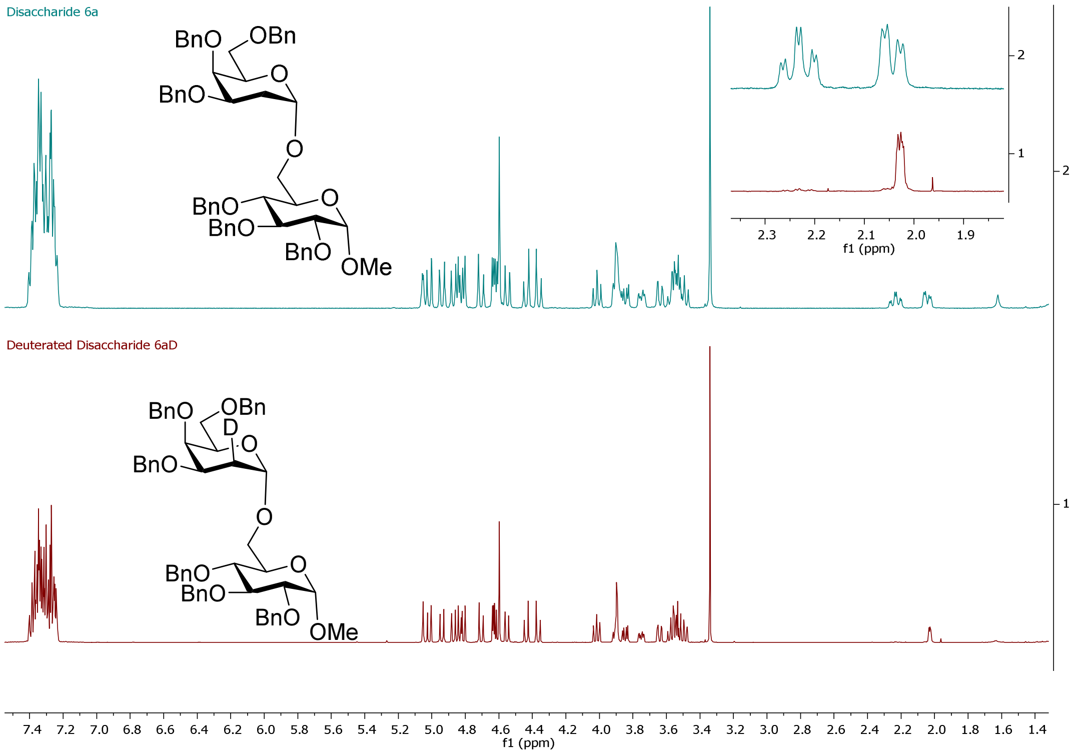

Figure S1. ^{1}H-NMR spectra for disaccharide **6a** and deuterated disaccharide **10** for comparison.

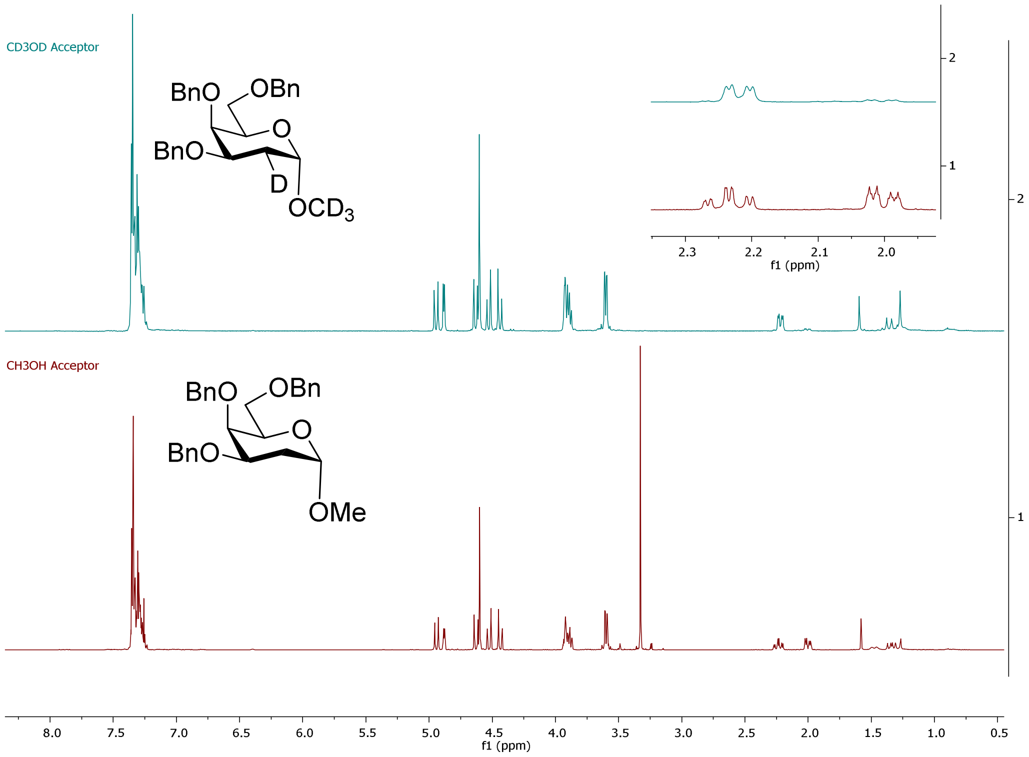

Figure S2. ^{1}H-NMR spectra for deuterated glycoside **11** and non-deuterated counterpart for comparison.

In Situ Anomerization Experiments:

Synthesis of Methyl 2,3,4-tri-*O*-benzyl-6-*O*-(2-deoxy-3,4,6-tri-*O*-benzyl-α/β-D-lyxo-hexapyranosyl)-α-D-glucopyranoside (3a)

The glycosyl donor **1a** (1 eq.) and acceptor **2a** (0.83 eq.) were weighed into a microwave vial and placed under vacuum for 1 h, after which time the microwave vial was filled with N_2. A solution mixture containing (*R*)-3,3′-Bis[3,5-bis(trifluoromethyl)phenyl]-1,1′-binaphthyl-2,2′-diyl hydrogenphosphate (0.1 eq.) and thiourea (0.1 eq.) in anhydrous CH_3CN (1 mL) was stirred for 30 mins., before adding it to the microwave vial containing **1a** and **2a**. The reaction mixture was stirred at RT for 4 h and then was purified by silica gel column chromatography (Hexane:EtOAc, 7:1 to 4:1) affording disaccharide **3a** as a colourless oil (62 mg 70%, 4:1 α:β). The spectroscopic data was in agreement with previously reported data.[1]

In Situ Anomerization test of 3a in the presence of 2a.

Disaccharide **3a** (4:1 α:β, 1 eq.), acceptor monosaccharide **2a** (1 eq.), Pd catalyst, and **L2** were weighed into a microwave vial and placed under vacuum for 1 h, after which time the microwave vial was filled with N_2. Anhydrous CH_2Cl_2 (1 mL) was added to the microwave vial and the reaction mixture was left stirring at 50 °C for 21 h without observing any change in the anomeric ratio (4:1 α:β) as monitored by NMR of the crude mixture. (Figure S3)

A)

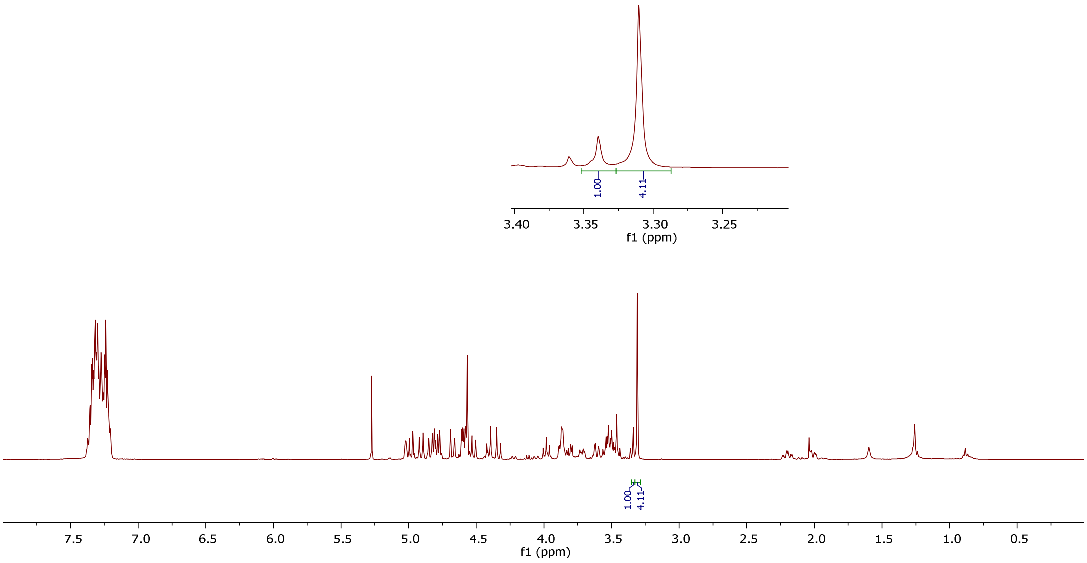

B)

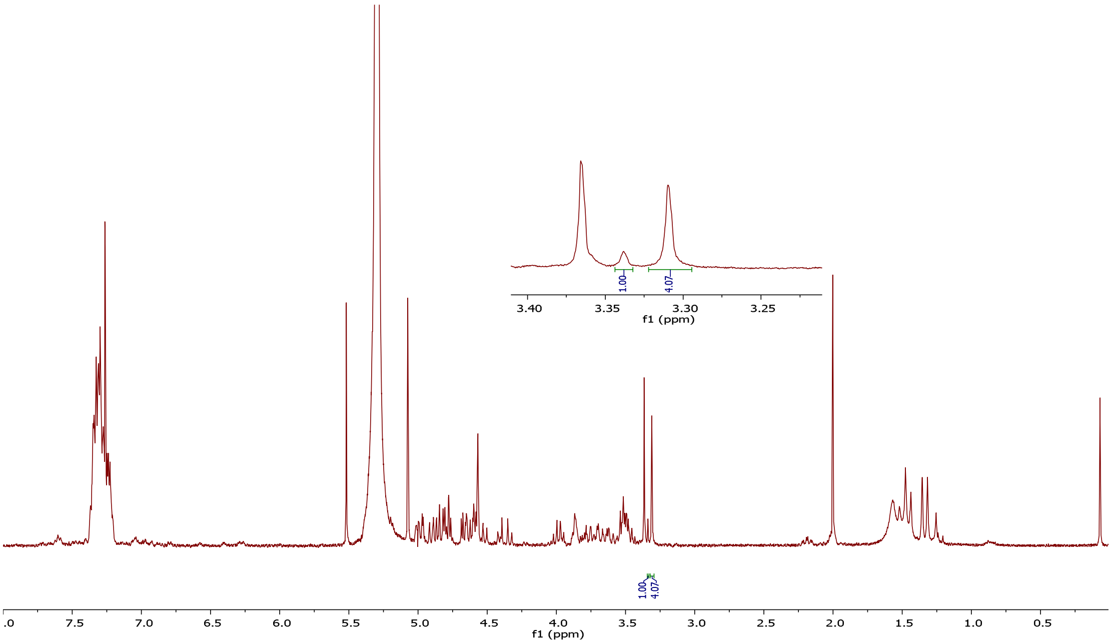

Figure S3. ^{1}H-NMR spectra in CD_2Cl_2 of : A) a 4:1 α:β mixture of **3a** and B) reaction mixture containing **3a** (4:1 α:β) after being subjected to the reaction conditions in the presence of Pd (II), **L2** and glycoside acceptor **2a**. This result suggests that the observed α-selectivity is not the result of in situ anomerization.

^{1}H-NMR experiments

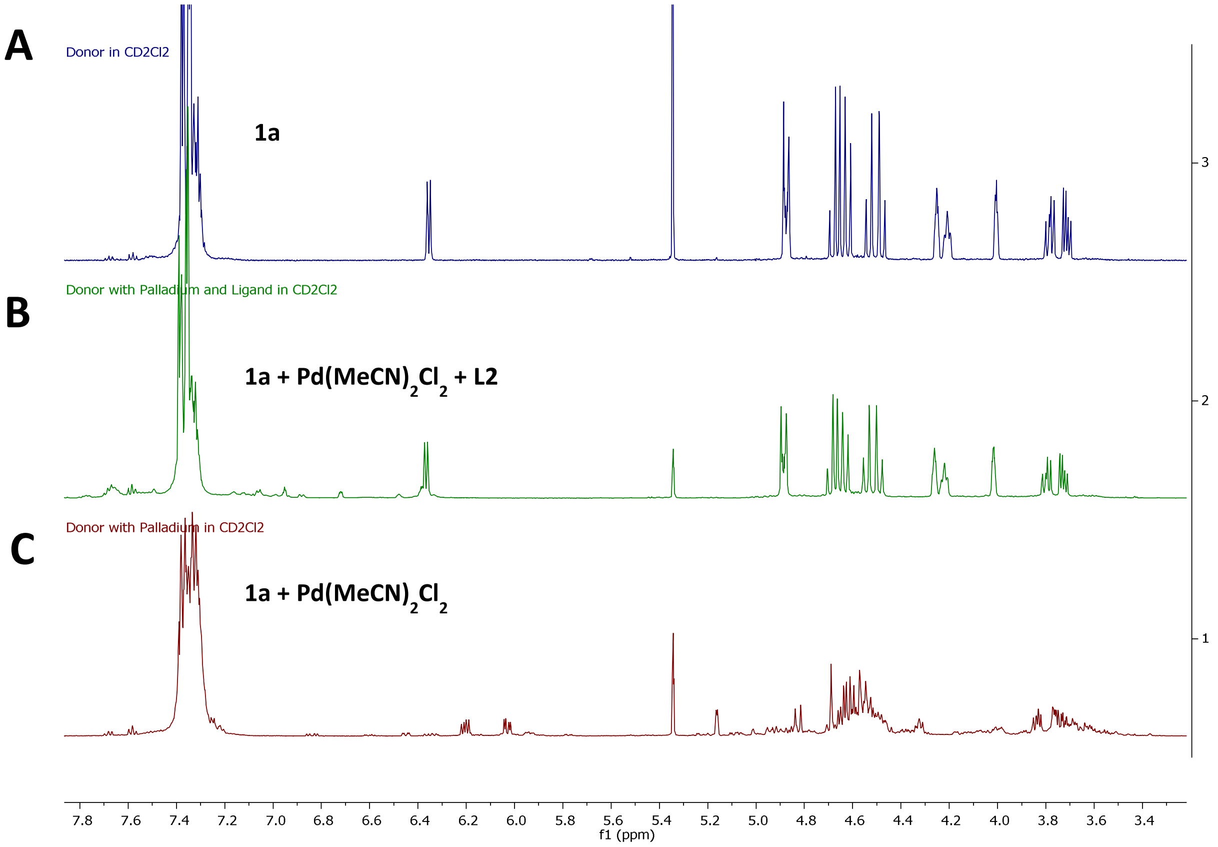

Figure S4. ^{1}H-NMR spectra in CD_2Cl_2 of A) Glycal donor **1a**; B) Glycal donor **1a**, Pd (II) and **L2** and C) 1/1 mixture of **1a** and Pd(II).

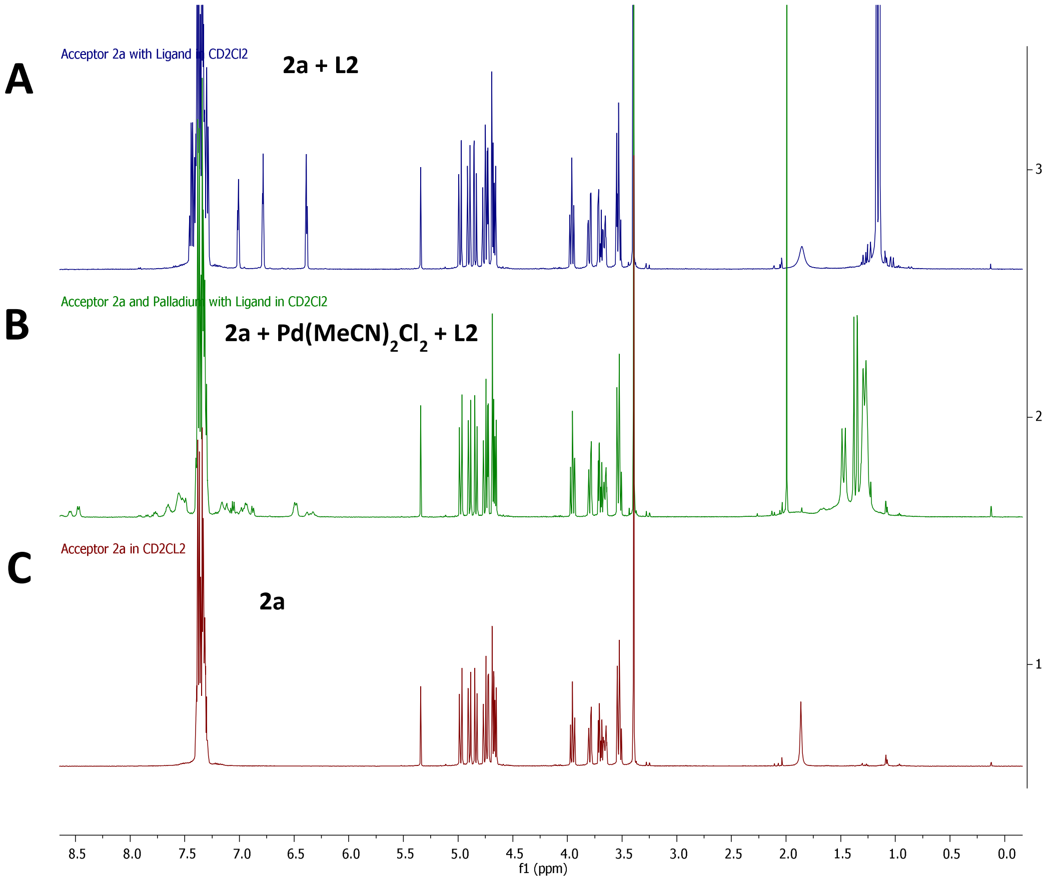

Figure S5. ^{1}H-NMR spectra in CD_2Cl_2 of A) 1/1 mixture of glycoside acceptor **2a** and **L2**; B) **2a**, Pd (II) and **L2** and C) glycoside acceptor **2a** .

NMR monitoring of the reaction

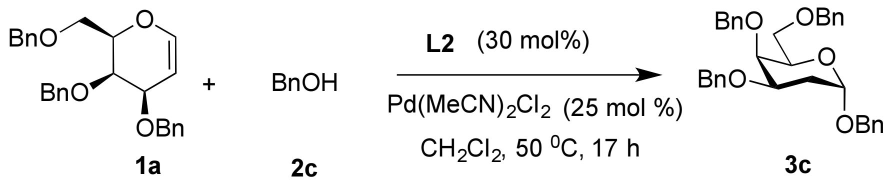

Following the *General Glycosylation Procedure*: glycosyl donor **1a** (100 mg, 0.240 mmol), benzyl alcohol **2c** (20 mg, 0.180 mmol), $Pd(CH_3CN)_2Cl_2$ (16 mg, 0.060 mmol) and ligand 2-(di-tert-butylphosphanyl)-1-phenyl-1H-pyrrole (21 mg, 0.072 mmol) were used. 0.5 mL aliquots were taken from the reaction and cool to 0 °C to stop the reaction and samples were immediately taken for NMR analysis, which was performed at room temperature. Note that the reaction does not proceed at room temperature.

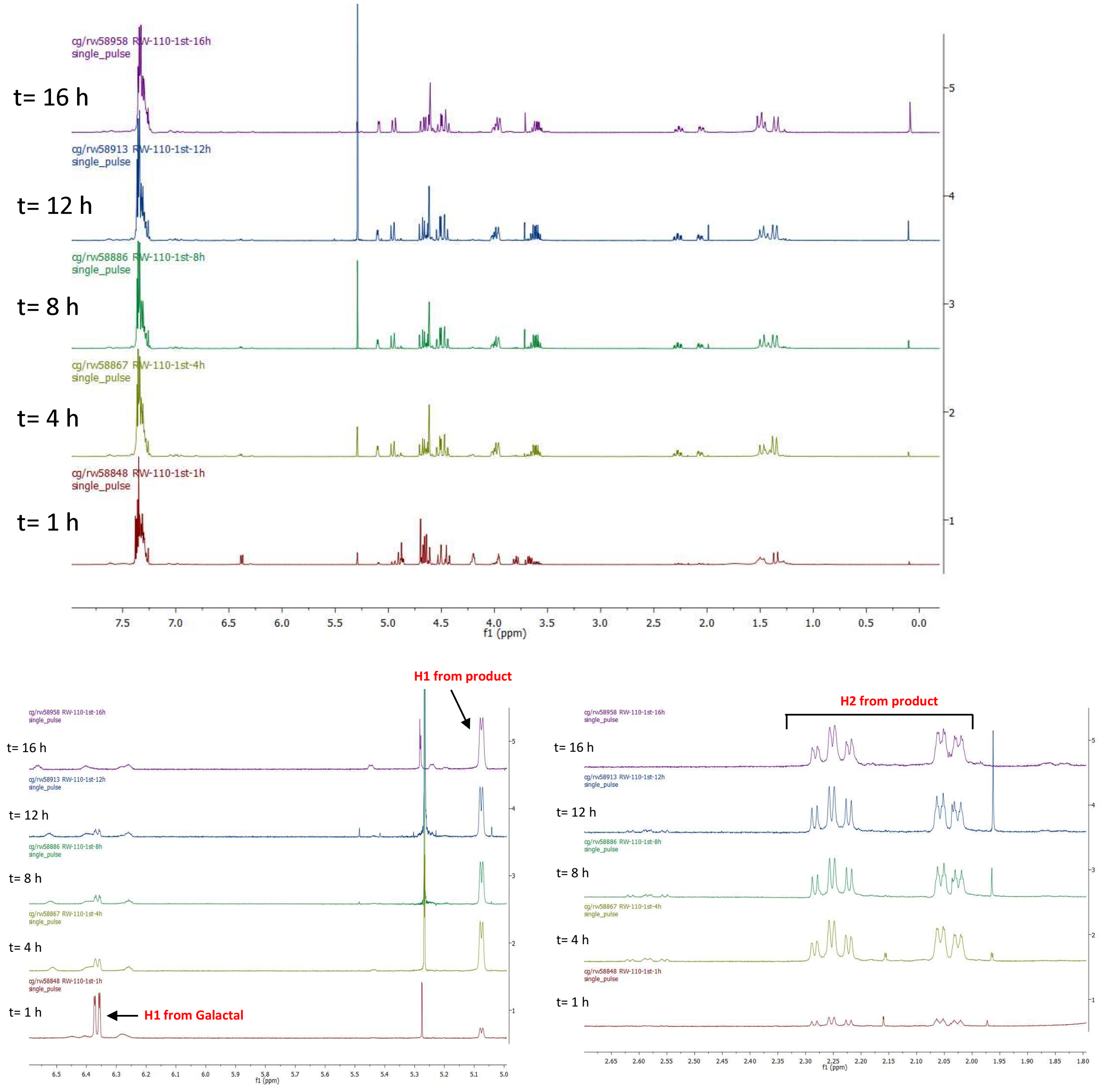

Figure S6. ^{1}H-NMR spectra of aliquots from reaction between **1a** and **2c** in CD_2Cl_2 at t = 1, 4, 8, 12 and 16h. Full spectra and expansions shown. Only signals from galactal donor and product are seen in the H-NMR.

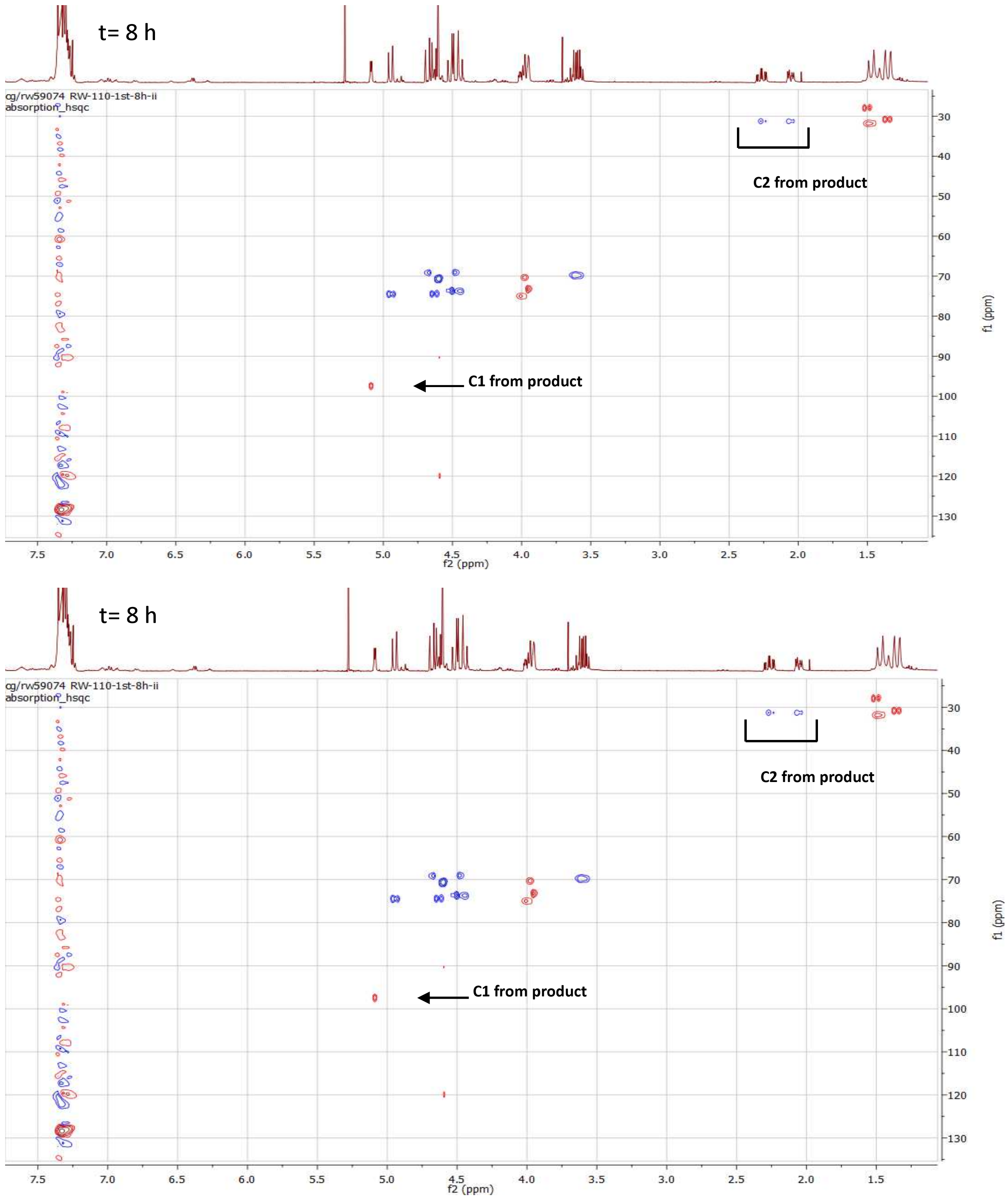

Figure S7A. HSQC spectra of aliquots from reaction between **1a** and **2c** in CD_2Cl_2 at t = 4 and 8 h showing anomeric region and C2 region of glycoside product.

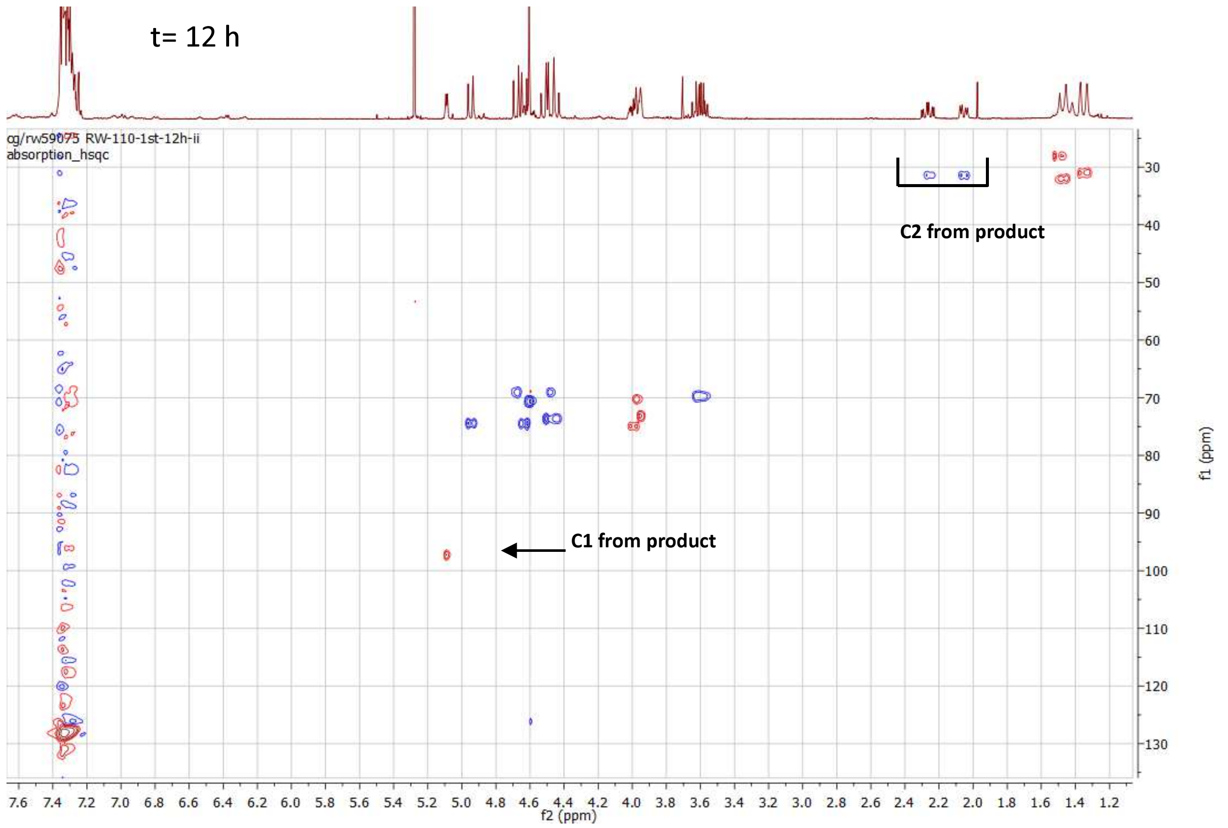

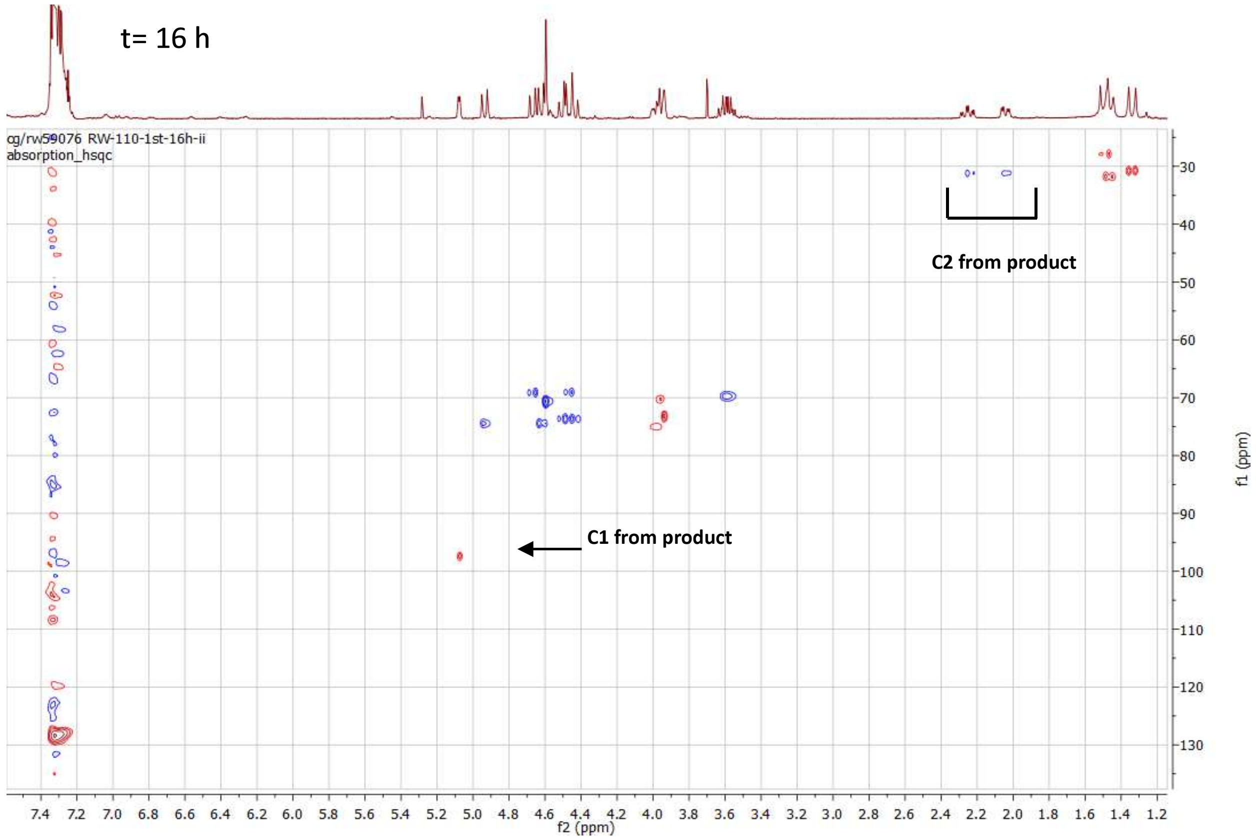

Figure S7B. HSQC spectra of aliquots from reaction between **1a** and **2c** in CD_2Cl_2 at t = 12 and 16h showing anomeric region and C2 region of glycoside product

References:

1. E. I. Balmond, D. M. Coe, M. C. Galan, E. M. McGarrigle, *Angew Chem Int Edit*. **2012**, 51, 9152-9155.
2. E. I. Balmond, D. Benito-Alifonso, D. M. Coe, R. W. Alder, E. M. McGarrigle, M. C. Galan, *Angew Chem Int Edit*. **2014**, 53, 8190-8194.
3. S. Das, D. Pekel, J. M. Neudorfl, A. Berkessel, *Angew Chem Int Edit*. **2015**, 54, 12479-12483.
4. H. C. Lin, J. F. Pan, Y. B. Chen, Z. P. Lin, C. H. Lin, *Tetrahedron*. *2011*, 67, 6362-6368.
5. B. G. Reddy, Y. D. Vankar, *Arkivoc*. **2004**, 12-19.

Compound (3a) Proton NMR (400 MHz, Chloroform-d)

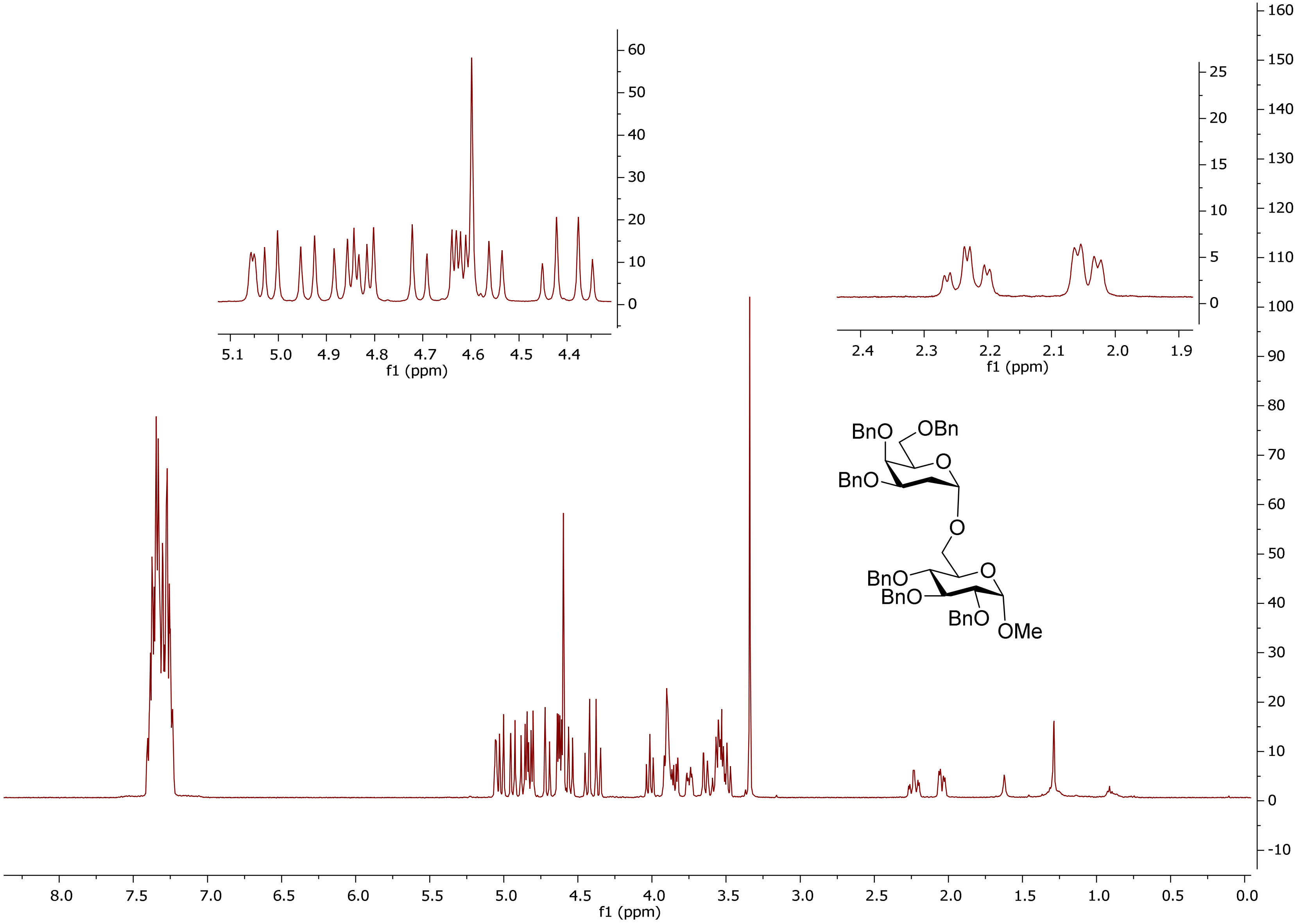

Compound (3a). Carbon (101 MHz, Chloroform-d)

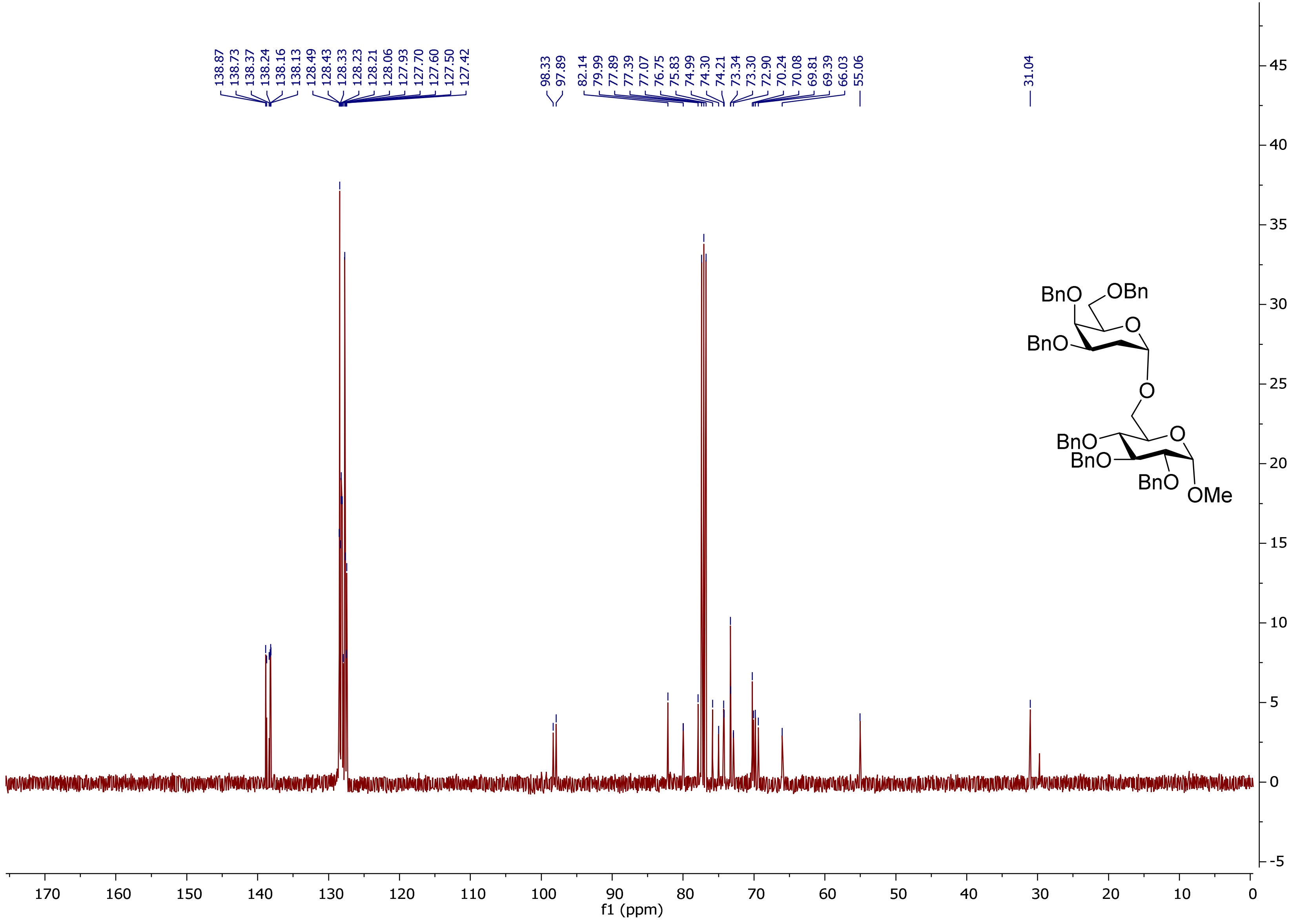

Compound (3a) HSQC Spectra

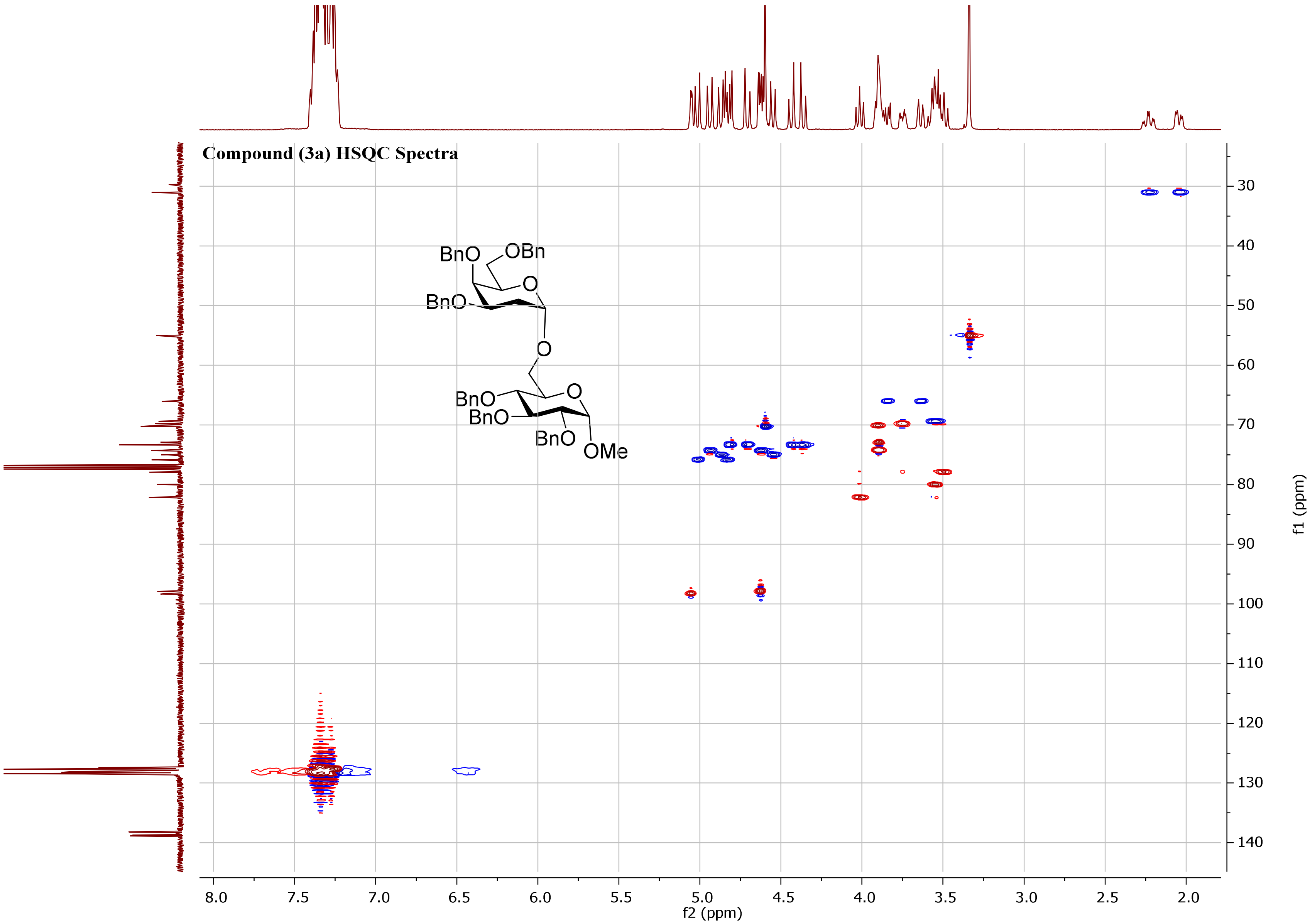

Compound (3b): Proton NMR (500 MHz, Chloroform-d)

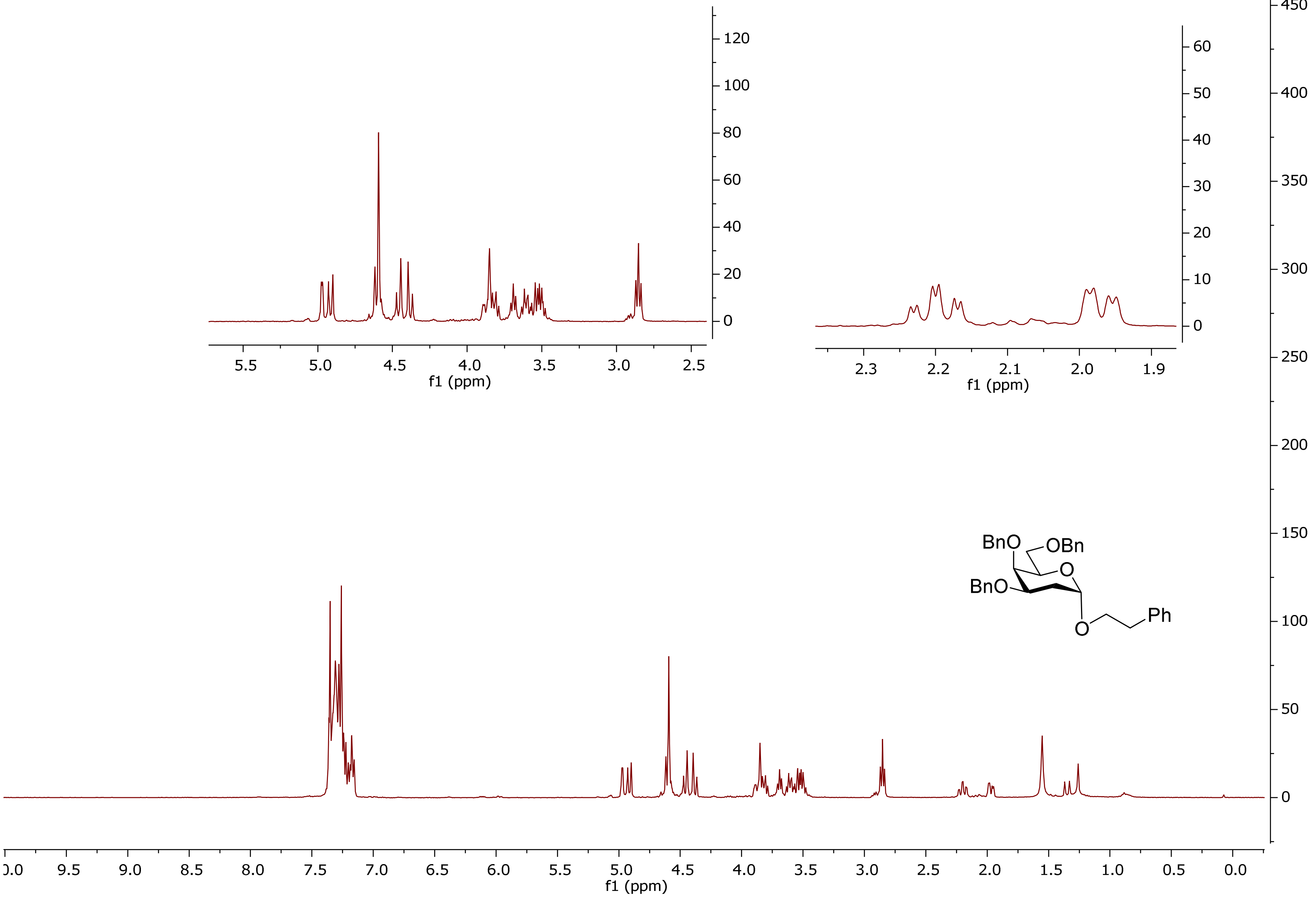

Compound (3b): Carbon-13 NMR (126 MHz, Chloroform-d)

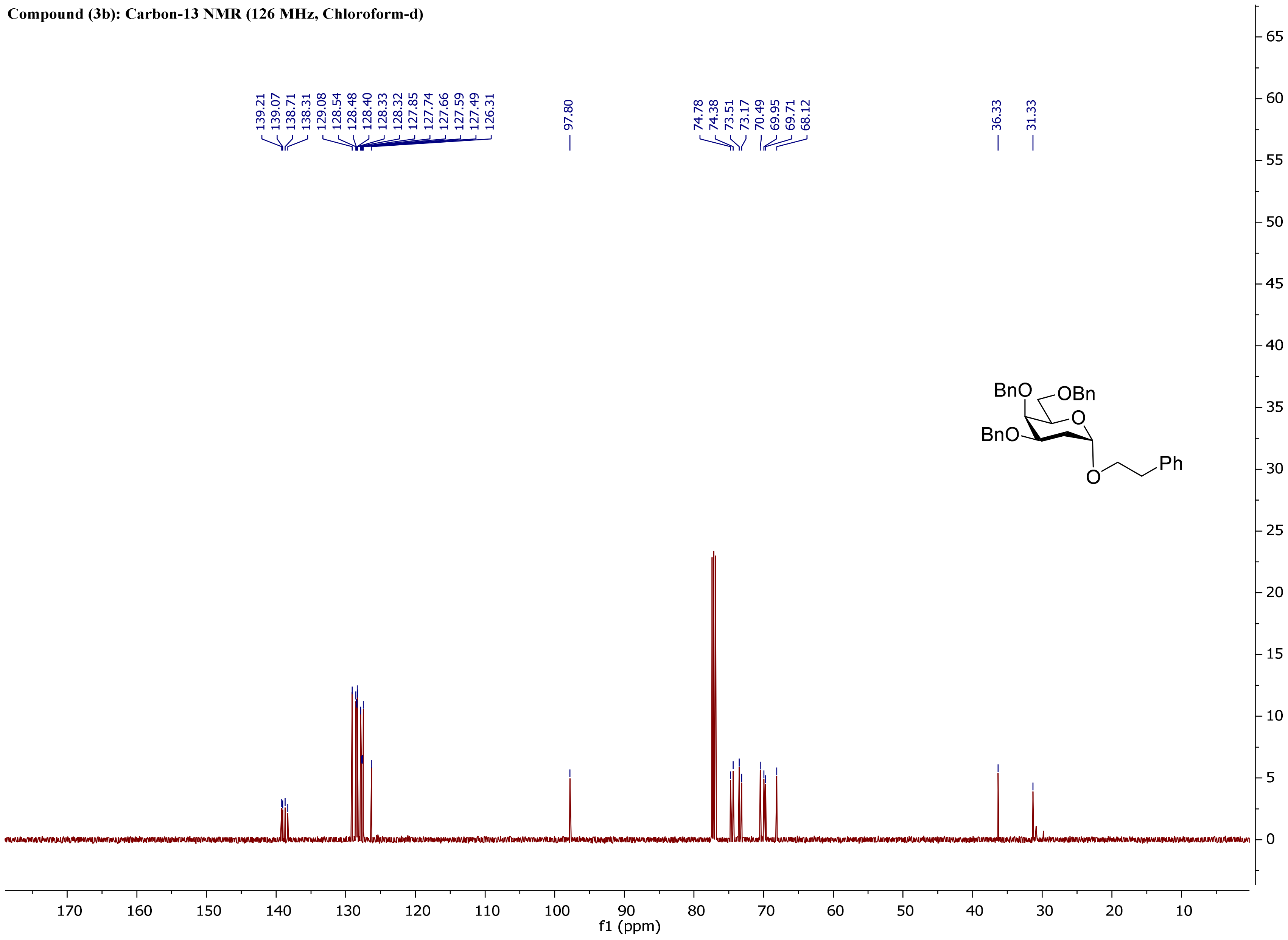

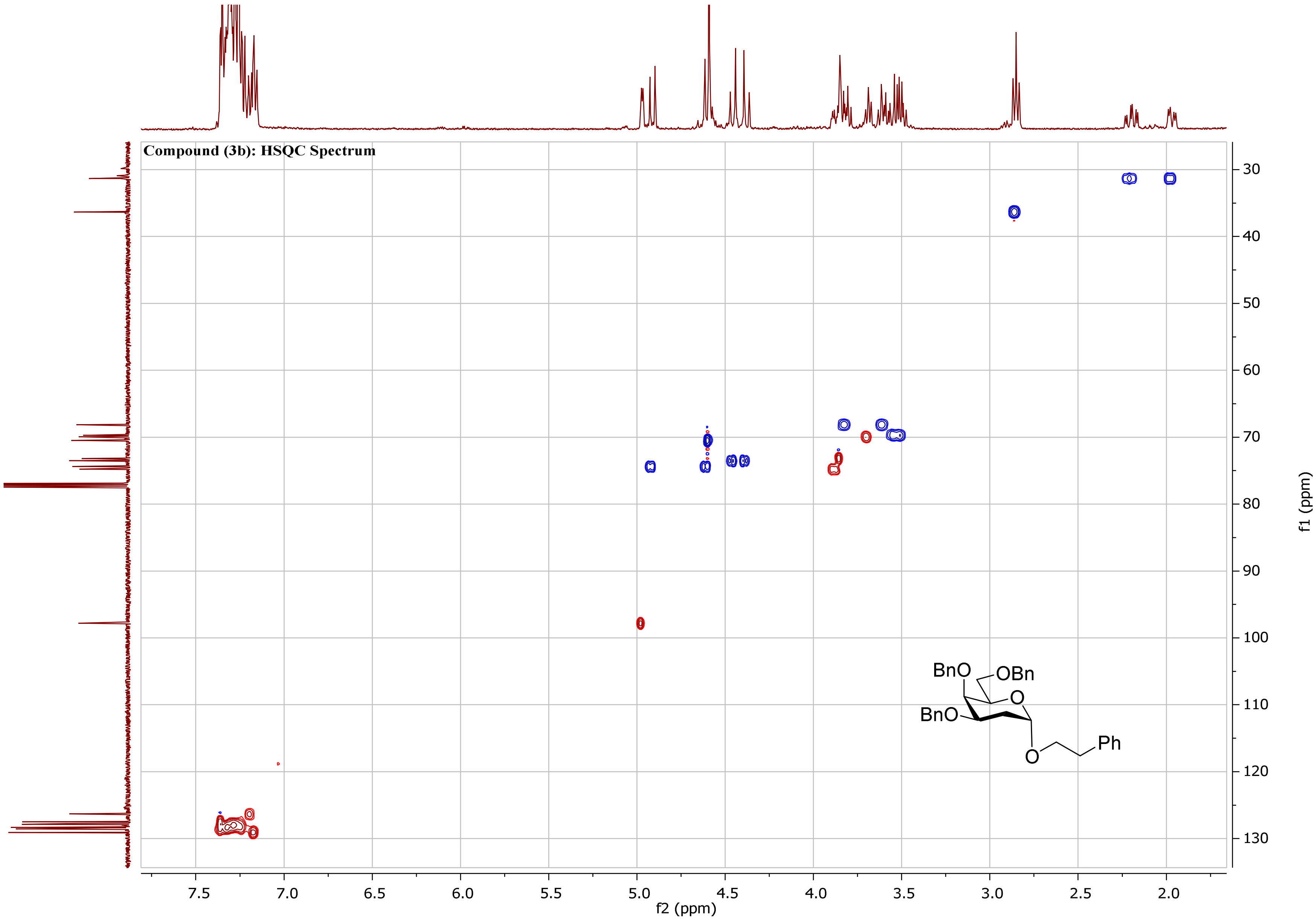
Compound (3b): HSQC Spectrum
BnO
OBn
O
BnO
O
Ph
f1 (ppm)
f2 (ppm)
30
40
50
60
70
80
90
100
110
120
130
7.5
7.0
6.5
6.0
5.5
5.0
4.5
4.0
3.5
3.0
2.5
2.0

Compound (3c): Proton NMR (500 MHz, Chloroform-d)

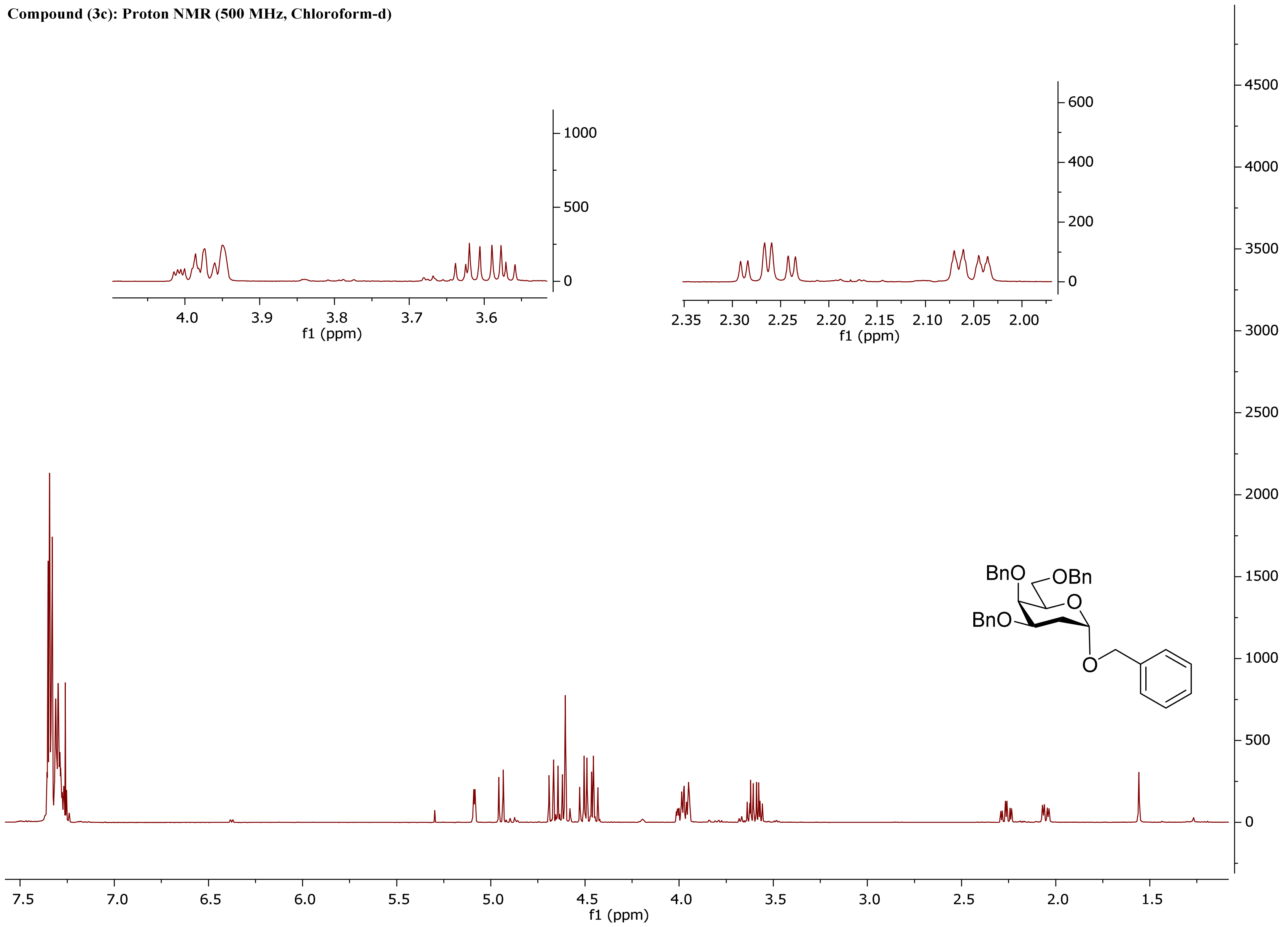

Compound (3c): Carbon-13 NMR (126 MHz, Chloroform-d)

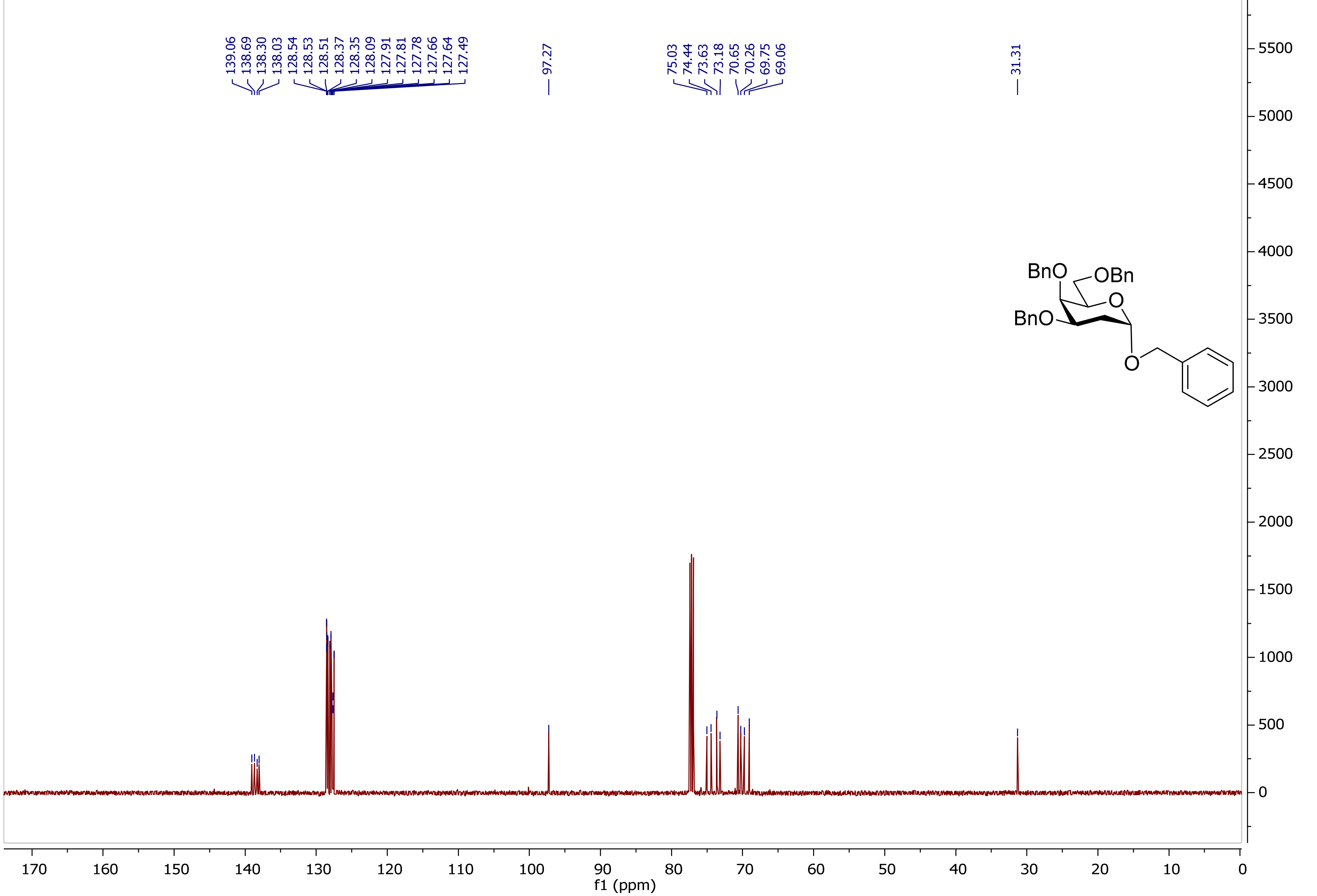

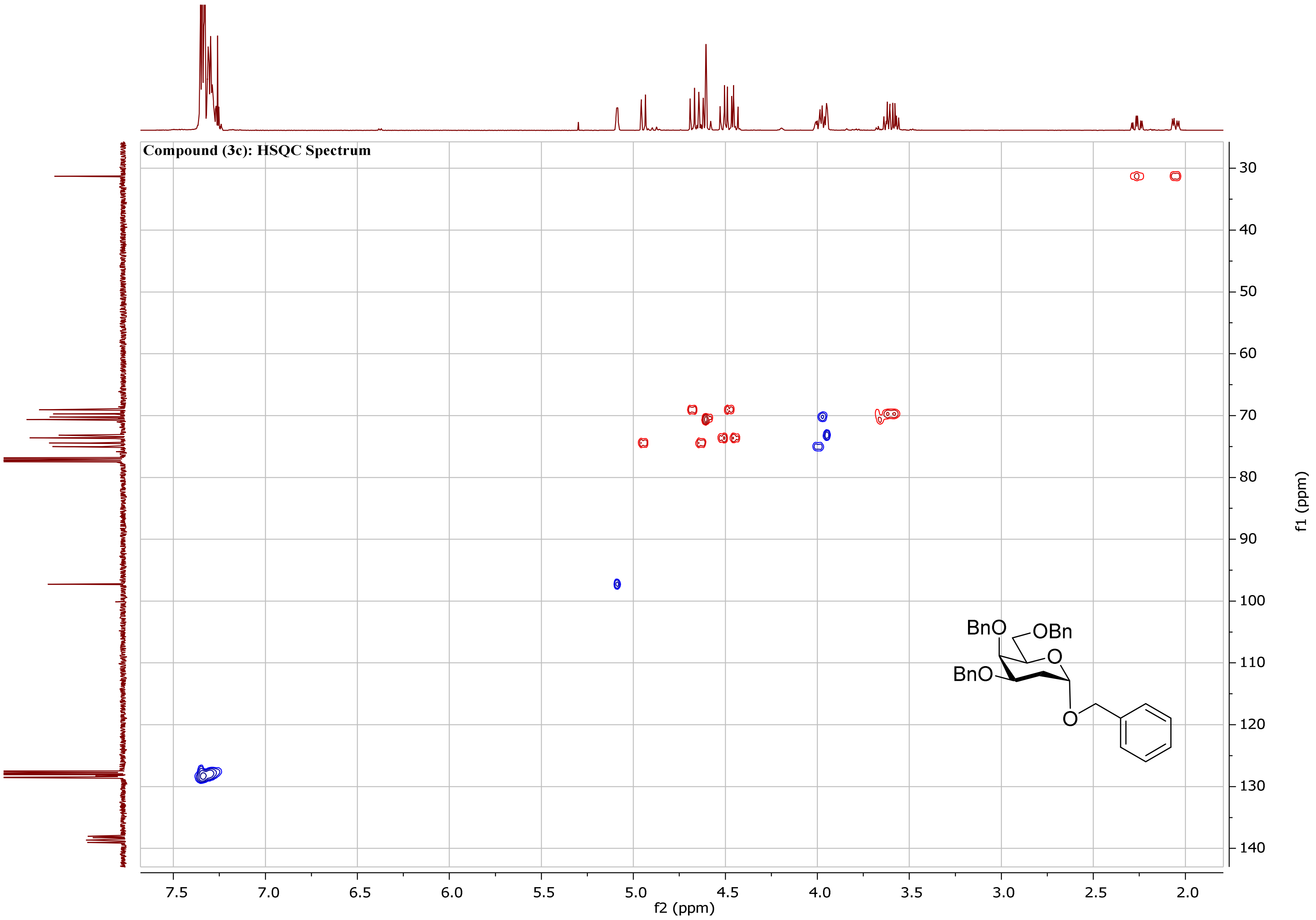
Compound (3c): HSQC Spectrum
BnO
OBn
O
BnO
O
f1 (ppm)
f2 (ppm)
30
40
50
60
70
80
90
100
110
120
130
140
7.5
7.0
6.5
6.0
5.5
5.0
4.5
4.0
3.5
3.0
2.5
2.0

Compound (3d): Proton NMR (500 MHz, Chloroform-d)

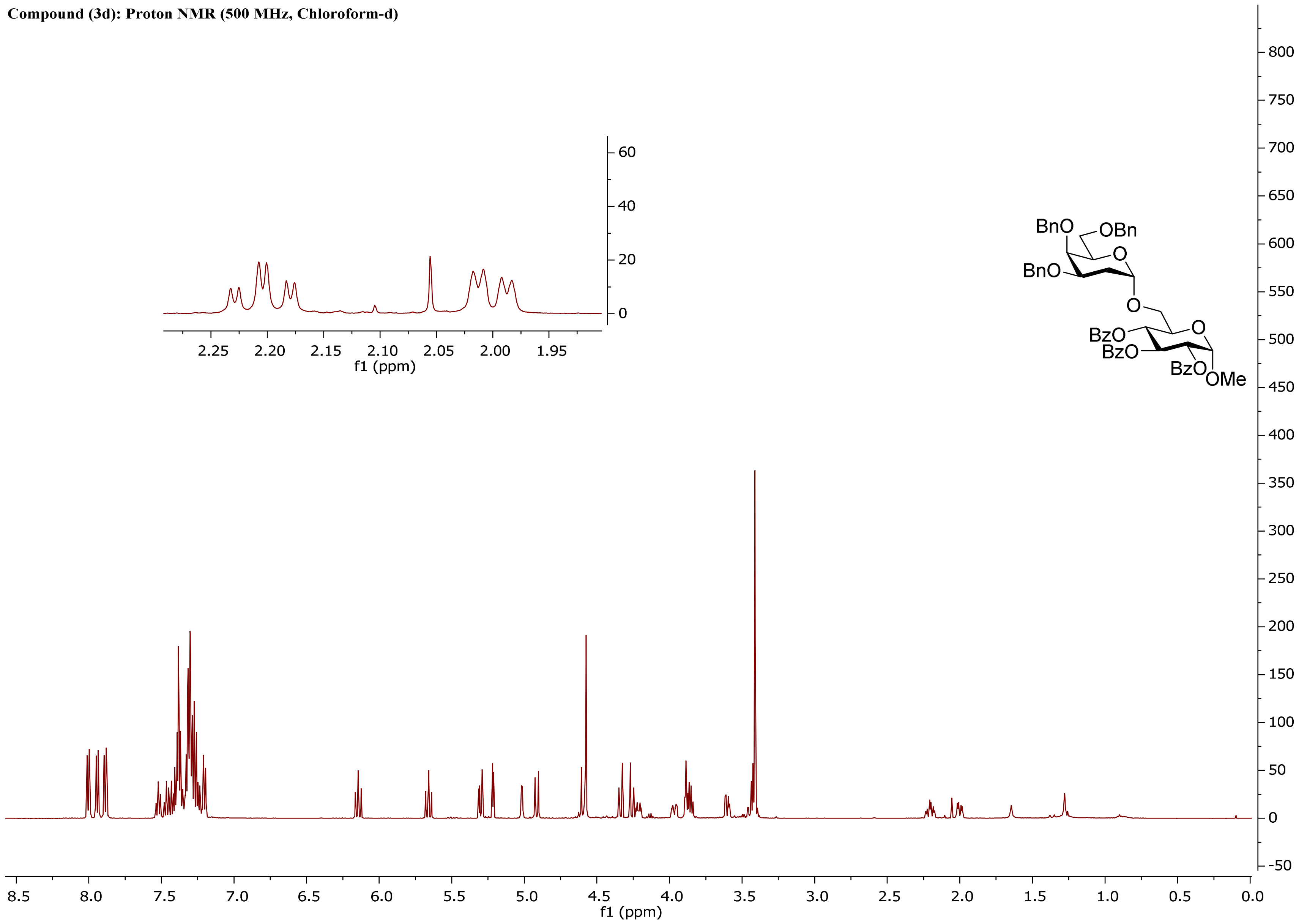

Compound (3d): Carbon-13 NMR (126 MHz, Chloroform-d)

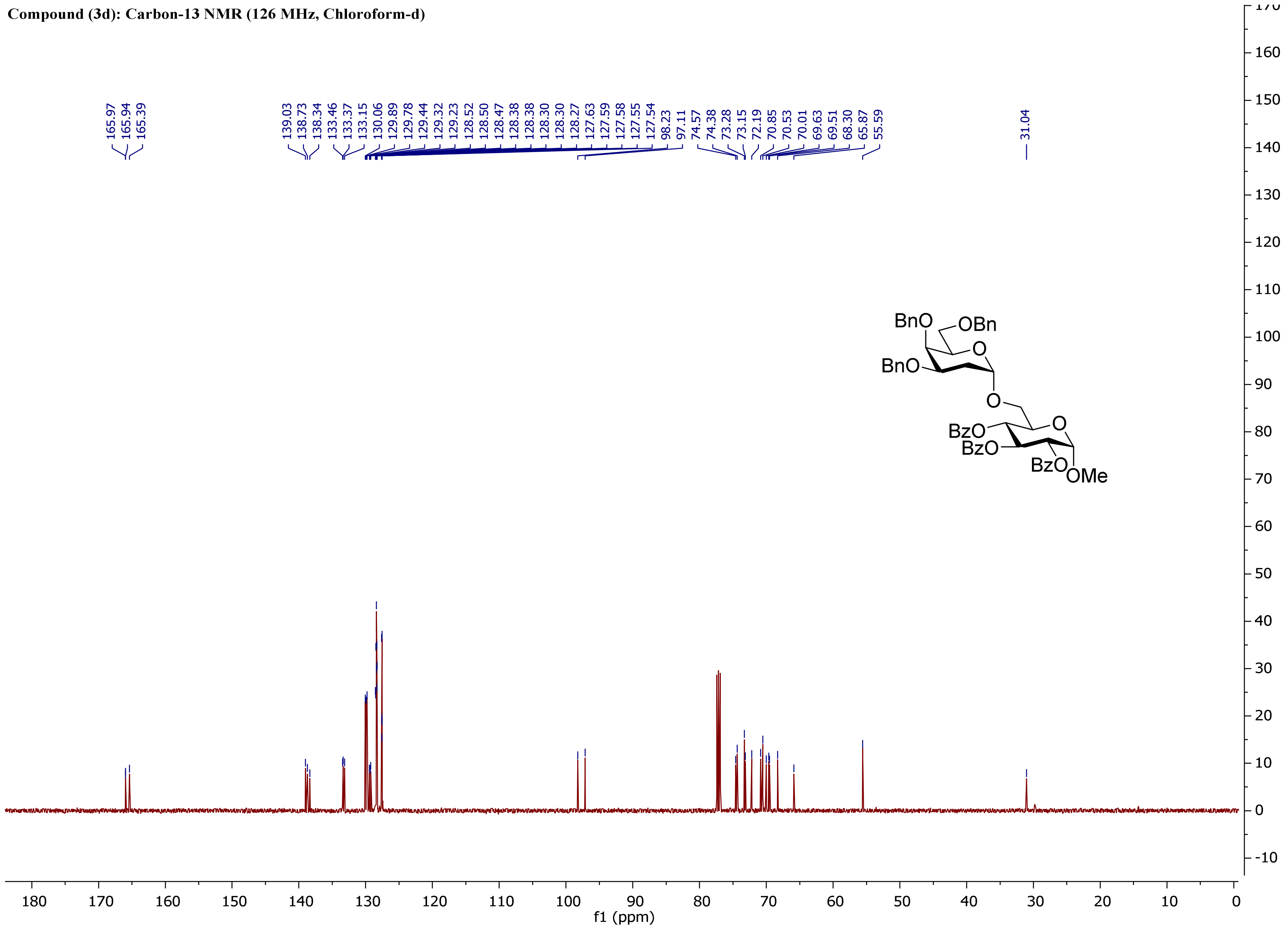

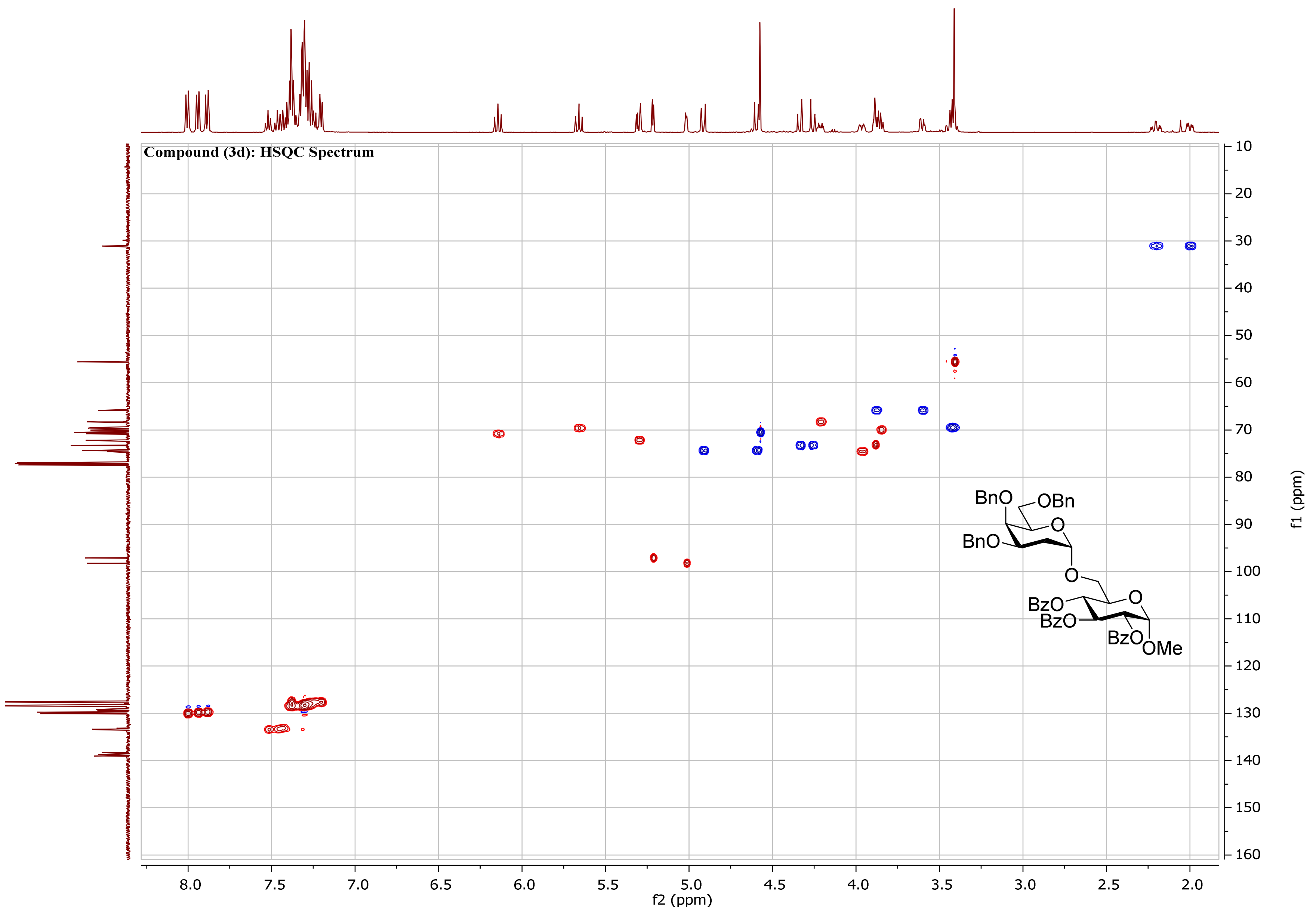
Compound (3d): HSQC Spectrum
BnO
OBn
O
BnO
O
BzO
BzO
O
BzO
OMe
f1 (ppm)
f2 (ppm)
10
20
30
40
50
60
70
80
90
100
110
120
130
140
150
160
8.0
7.5
7.0
6.5
6.0
5.5
5.0
4.5
4.0
3.5
3.0
2.5
2.0

Compound (3e): Proton NMR (400 MHz, Chloroform-d)

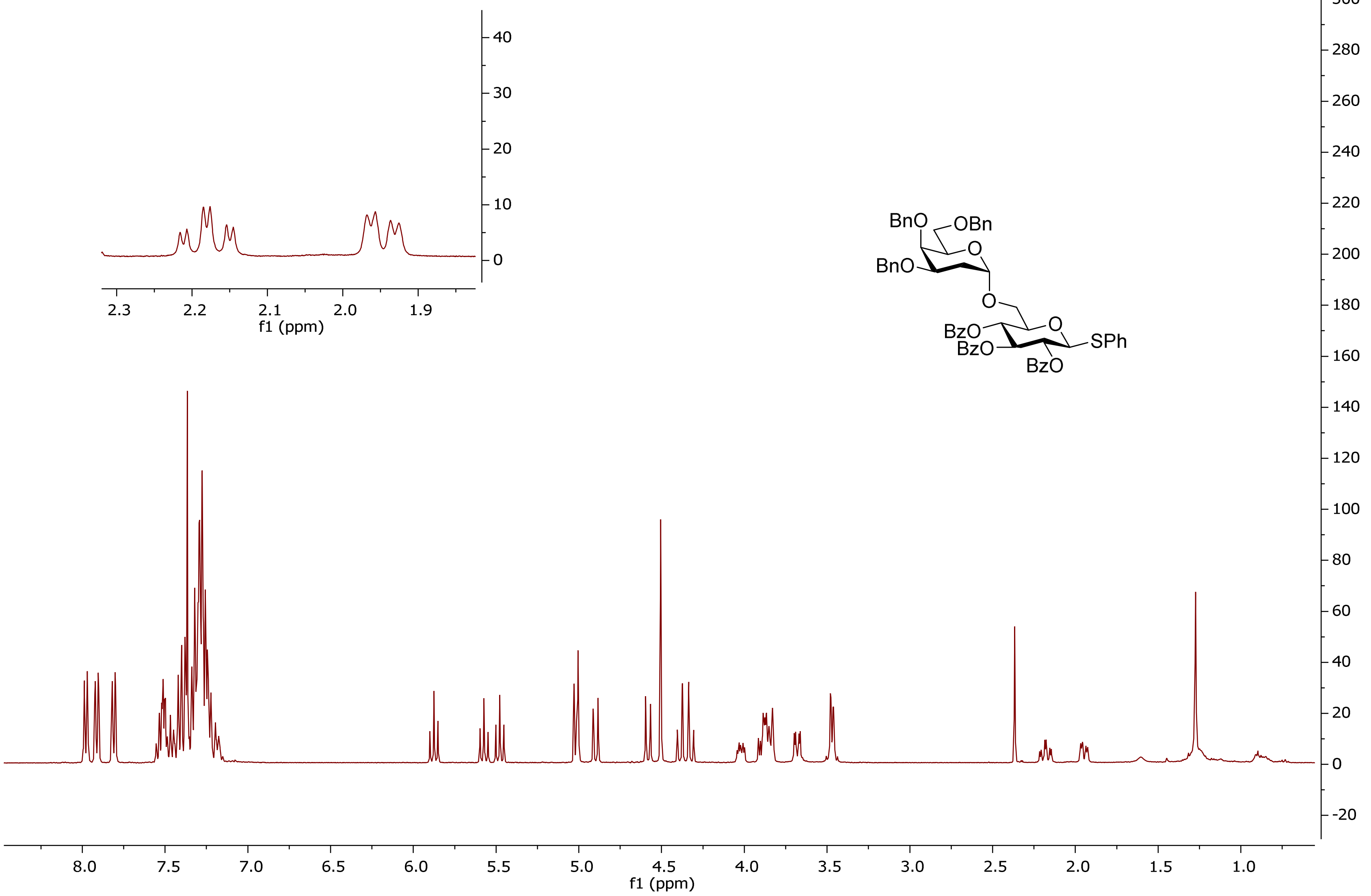

Compound (3e): Carbon-13 (101 MHz, Chloroform-d)

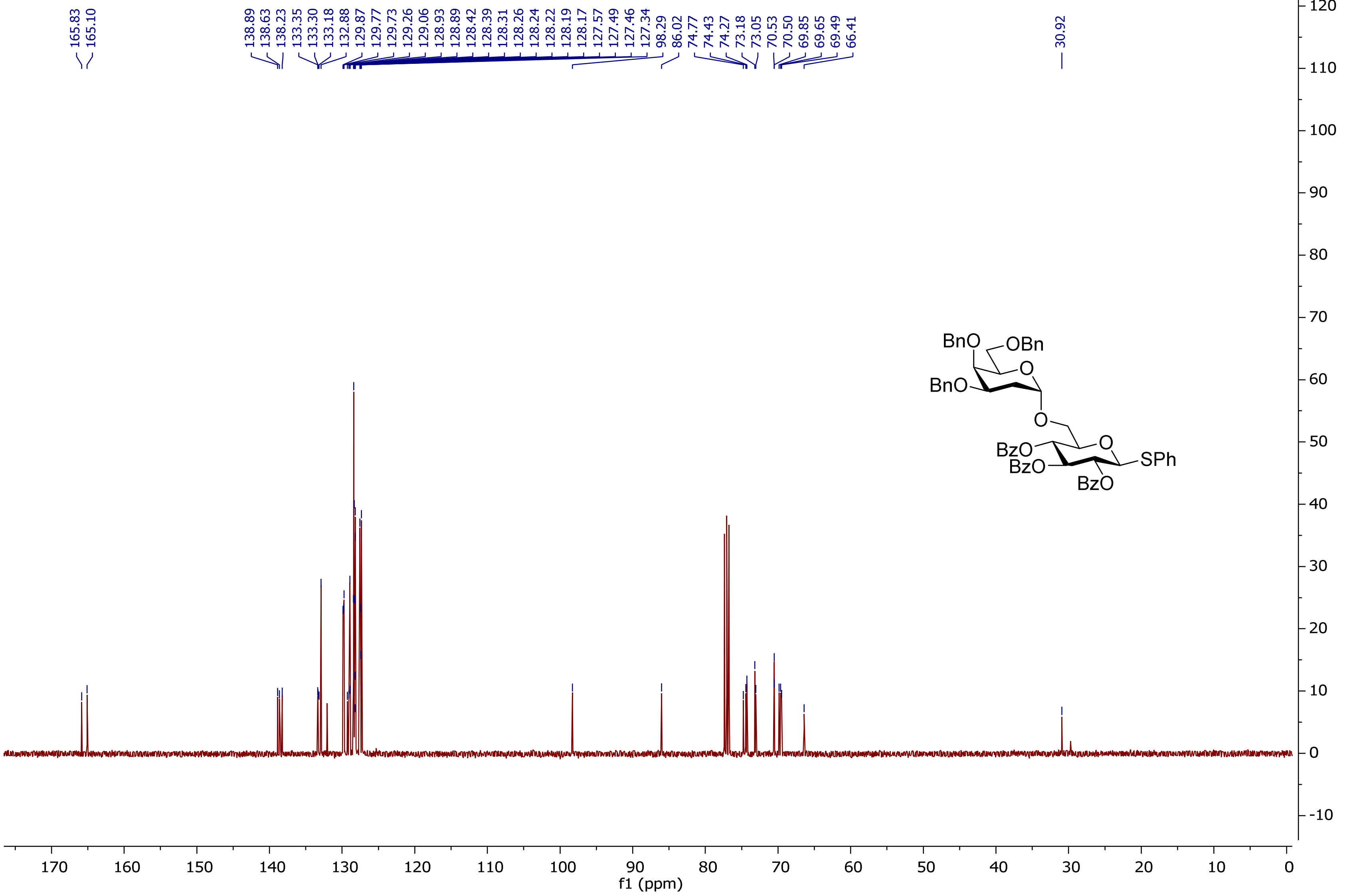

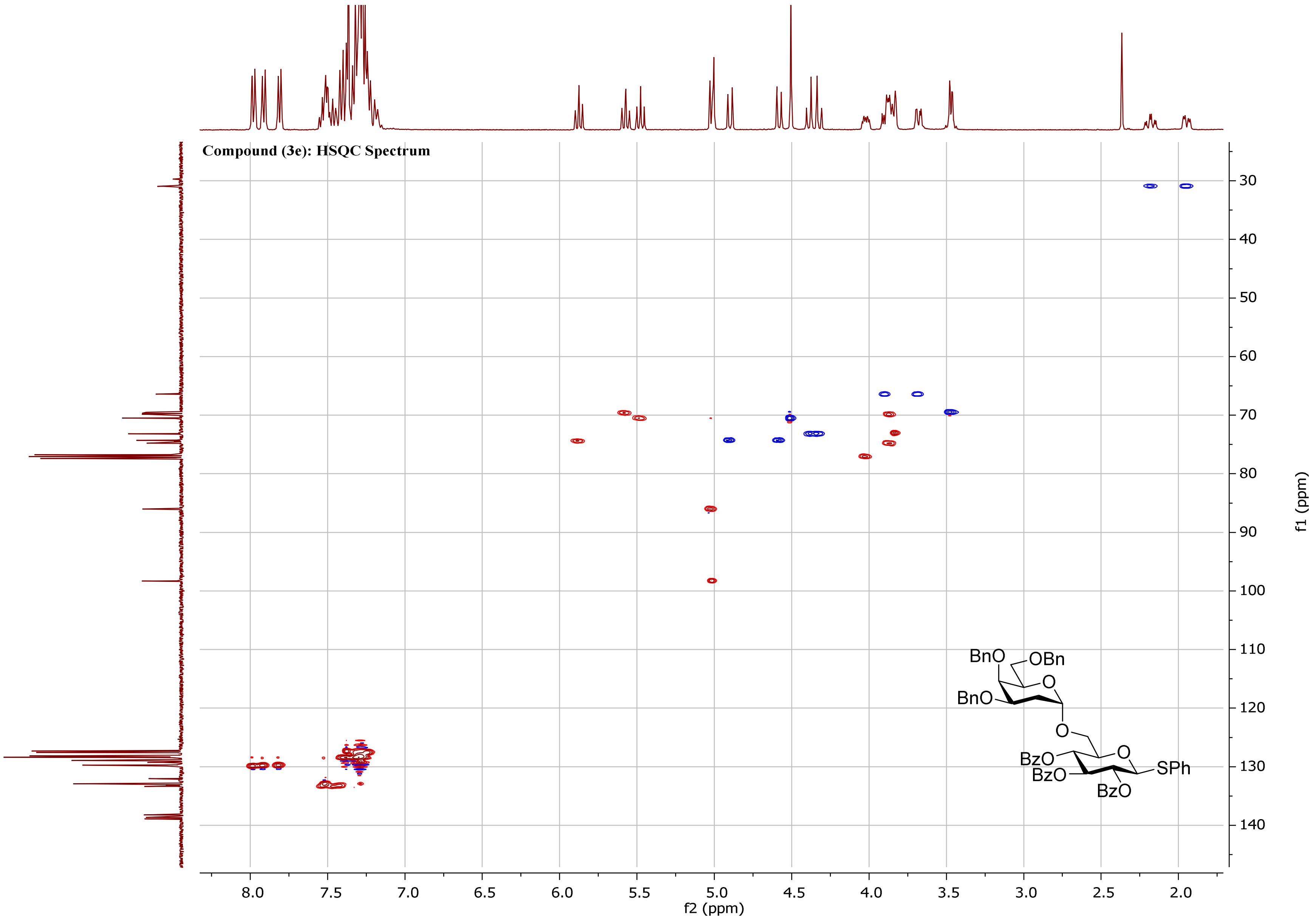
Compound (3e): HSQC Spectrum
f2 (ppm)
f1 (ppm)
BnO
OBn
O
BnO
O
BzO
BzO
O
SPh
BzO
8.0
7.5
7.0
6.5
6.0
5.5
5.0
4.5
4.0
3.5
3.0
2.5
2.0
30
40
50
60
70
80
90
100
110
120
130
140

Compound (3f) Proton NMR (500 Mz Chlorofrom-d)

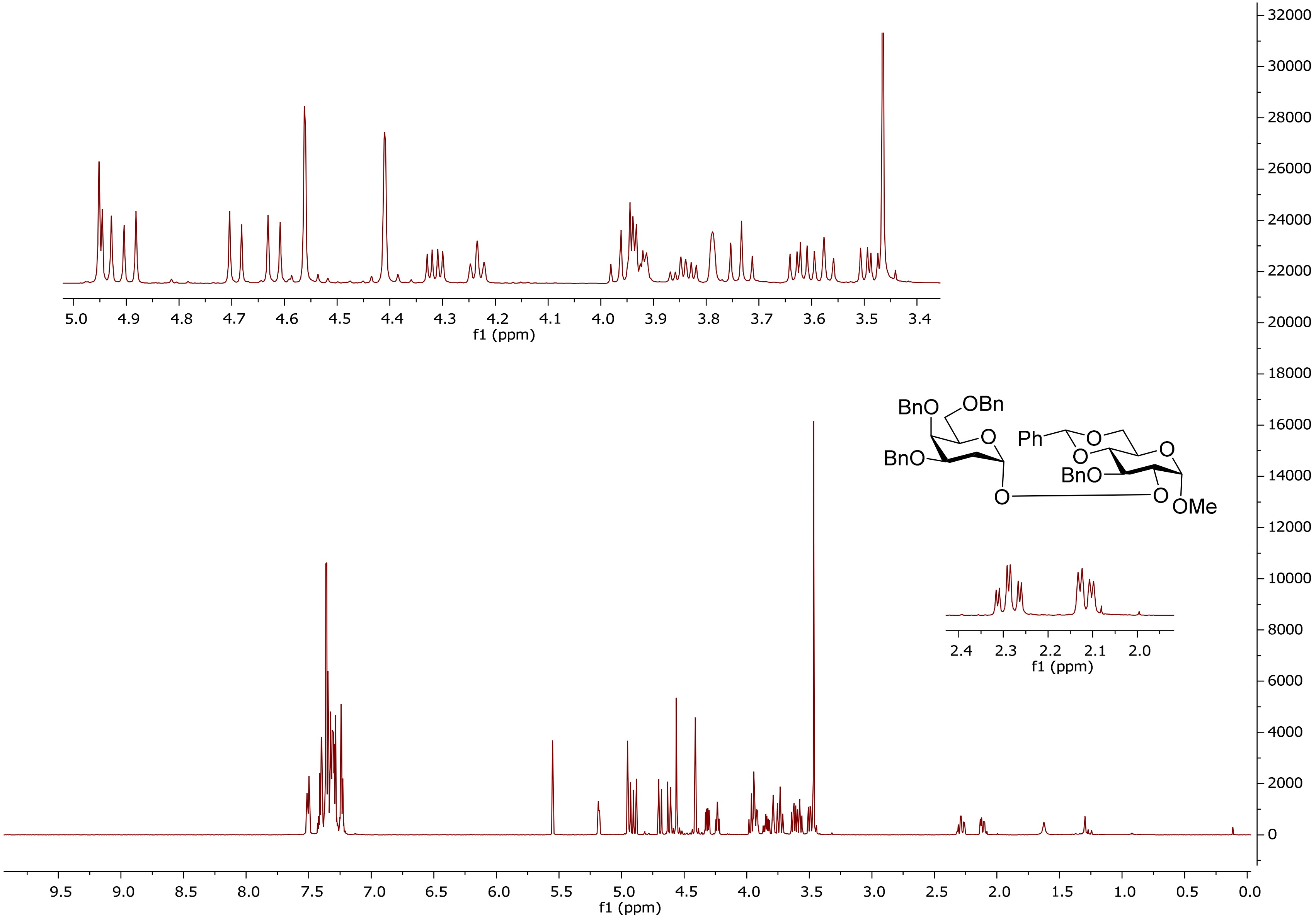

Compound (3f) : Carbon NMR (126 Mz Chlorofrom-d)

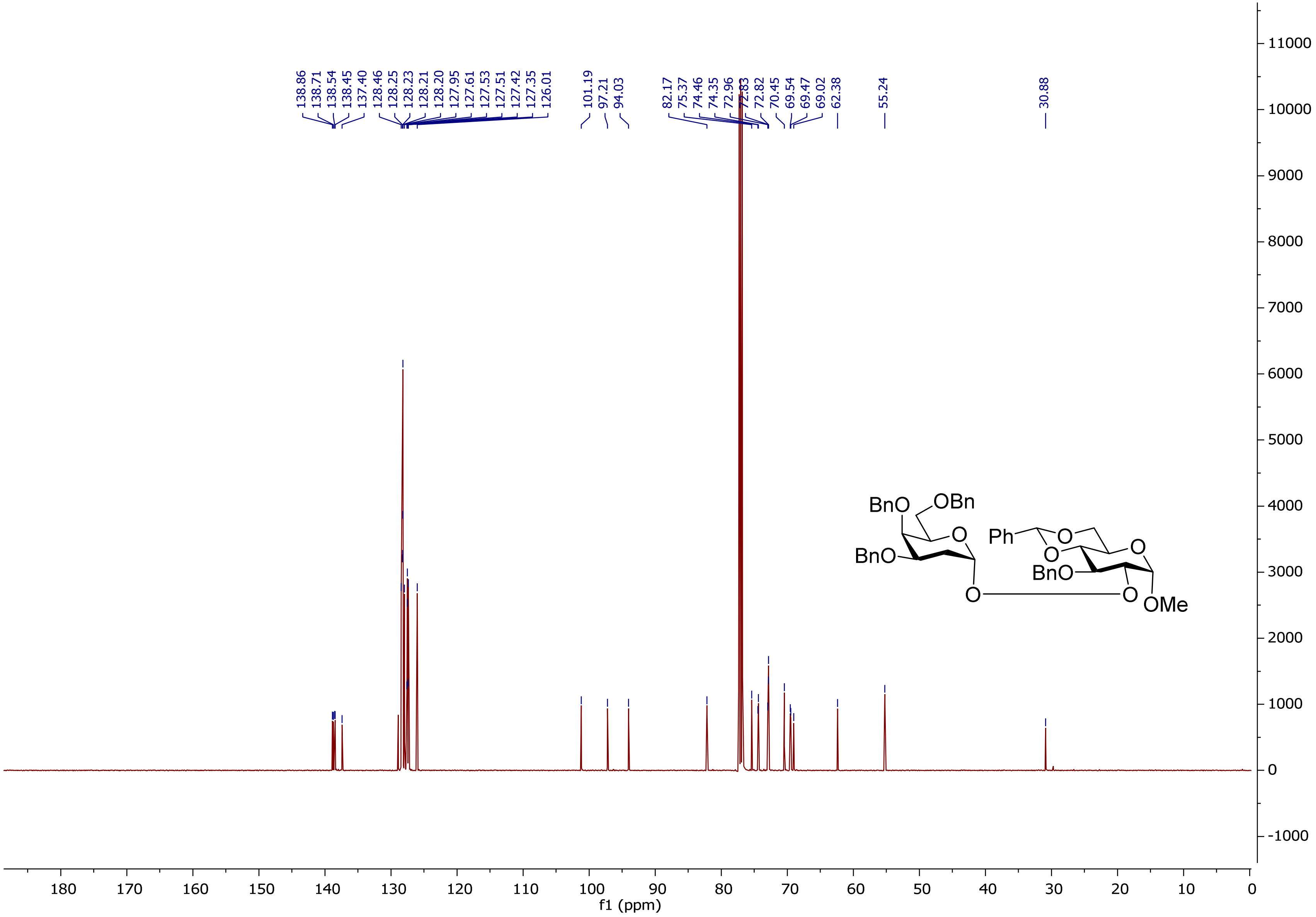

Compound (3f): HSQC spectra

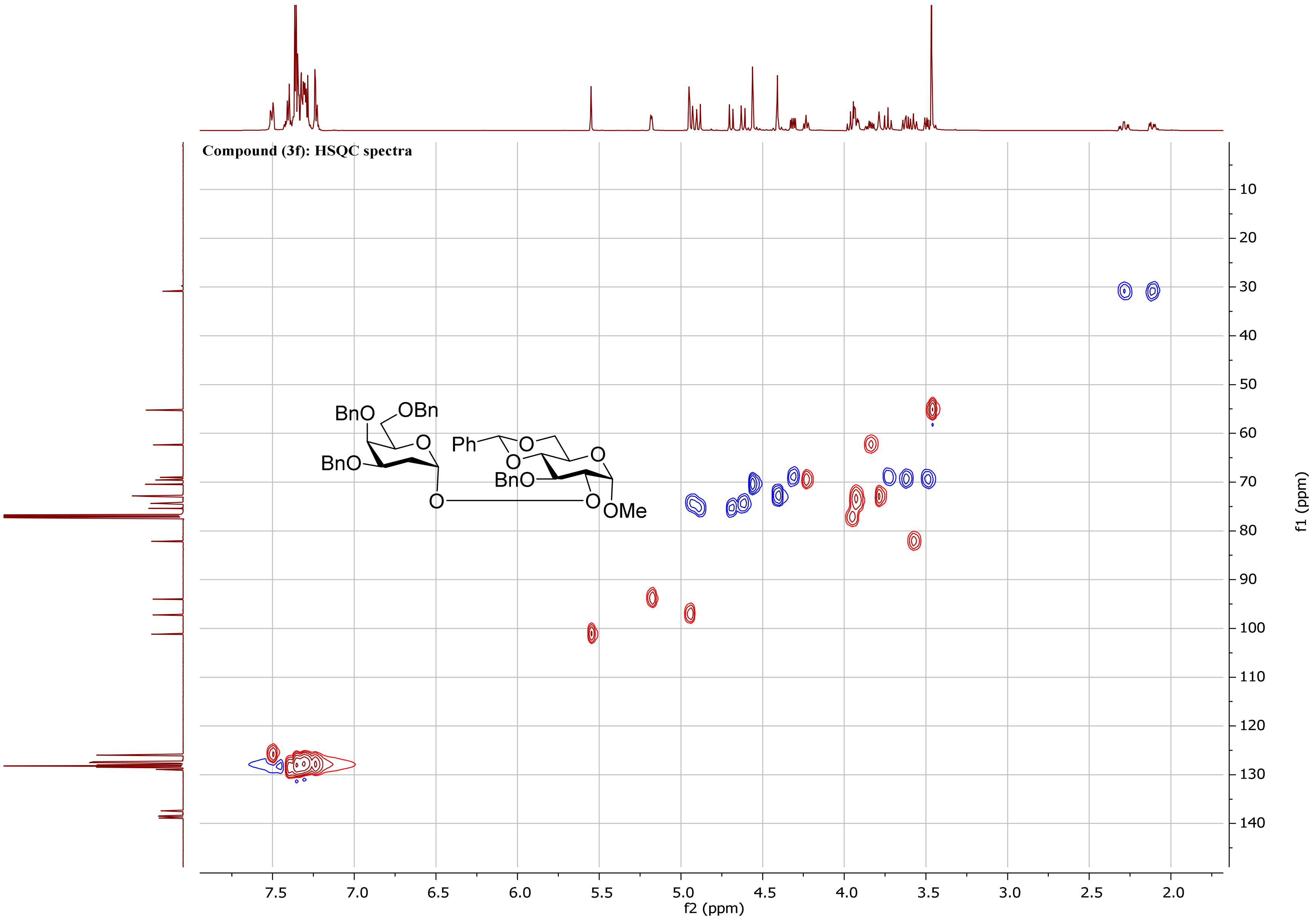

Compound (3g): Proton NMR (400 MHz, Chloroform-d)

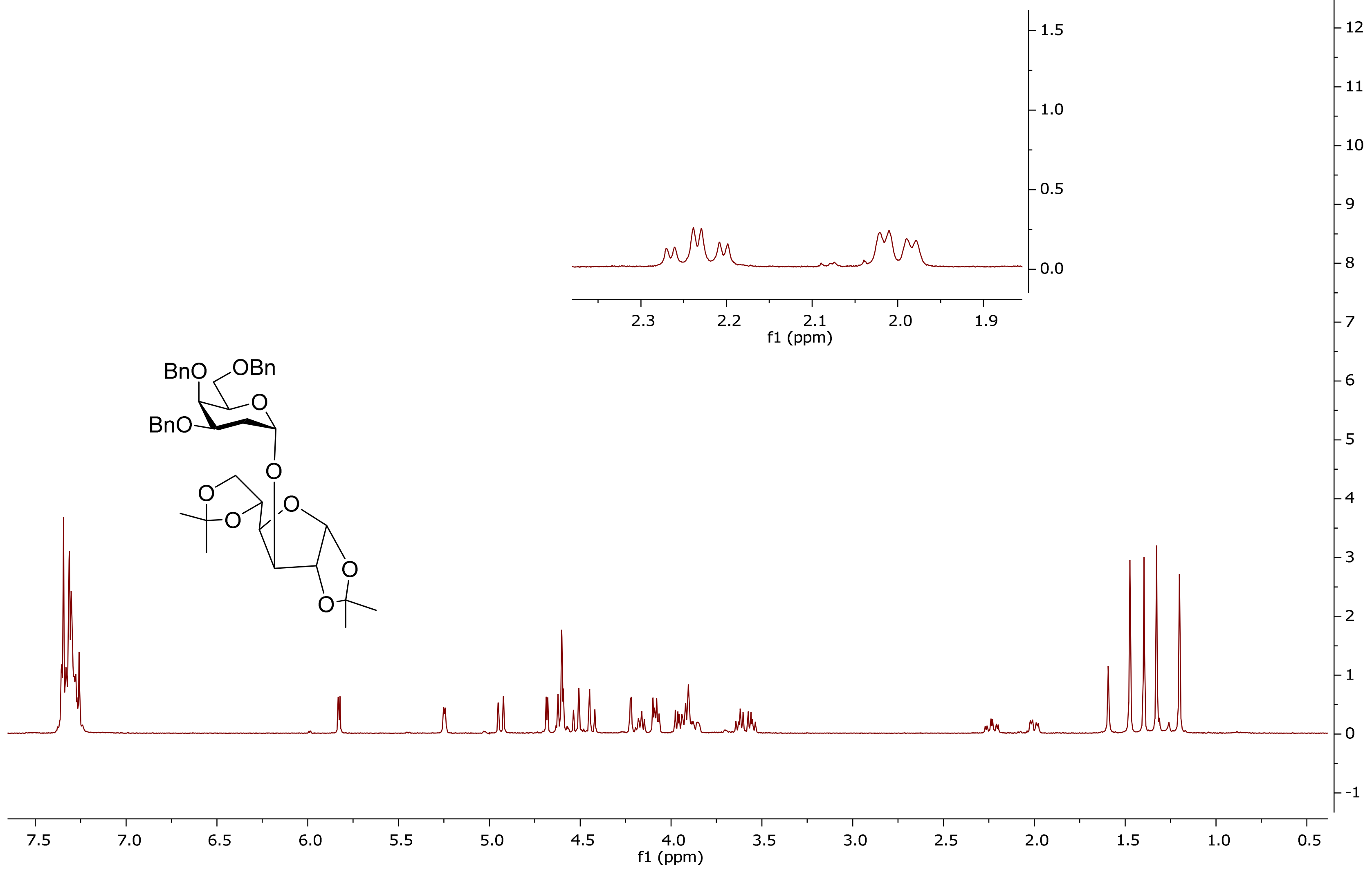

Compound (3g): Carbon NMR (101 MHz, Chloroform-d)

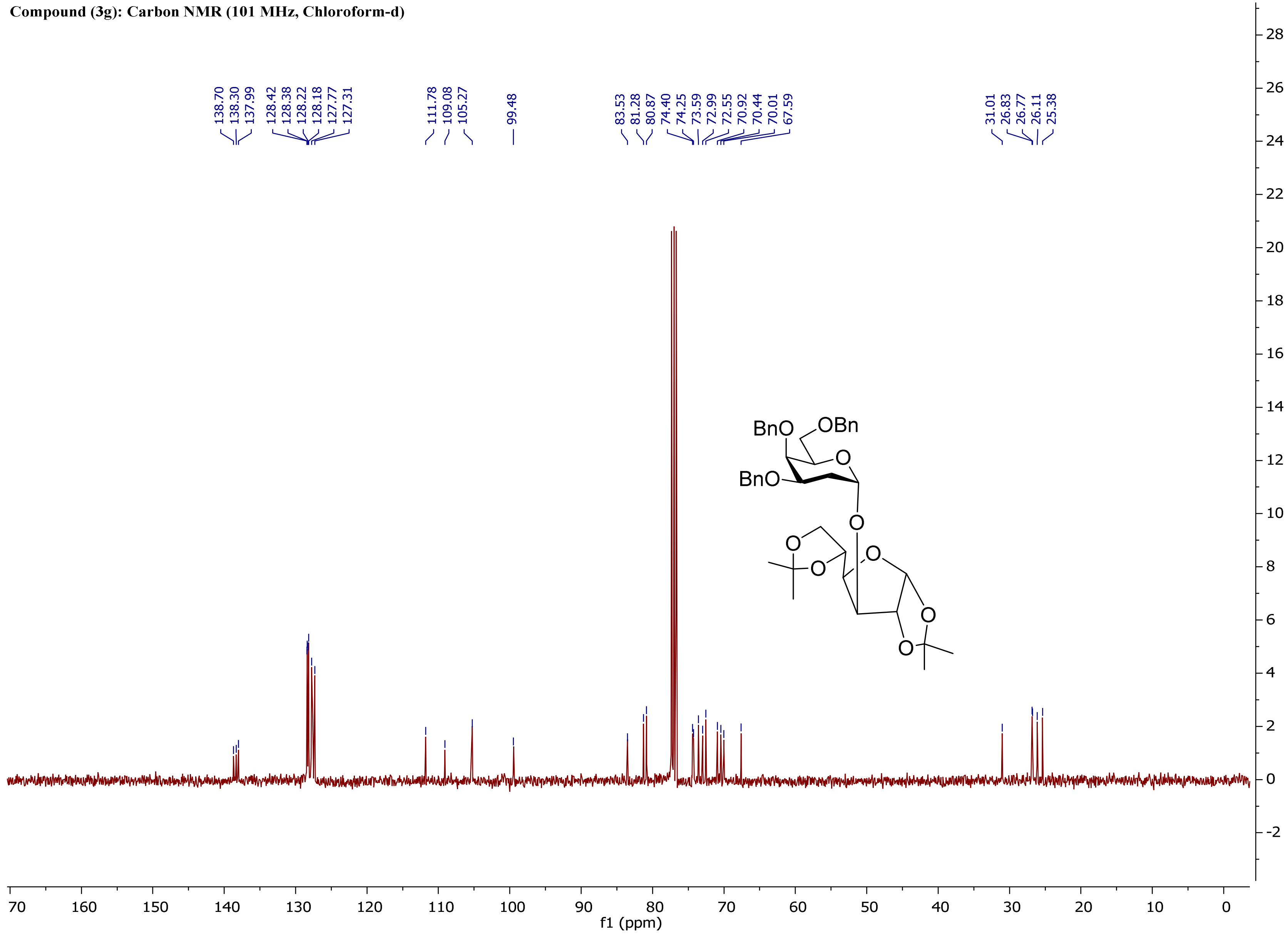

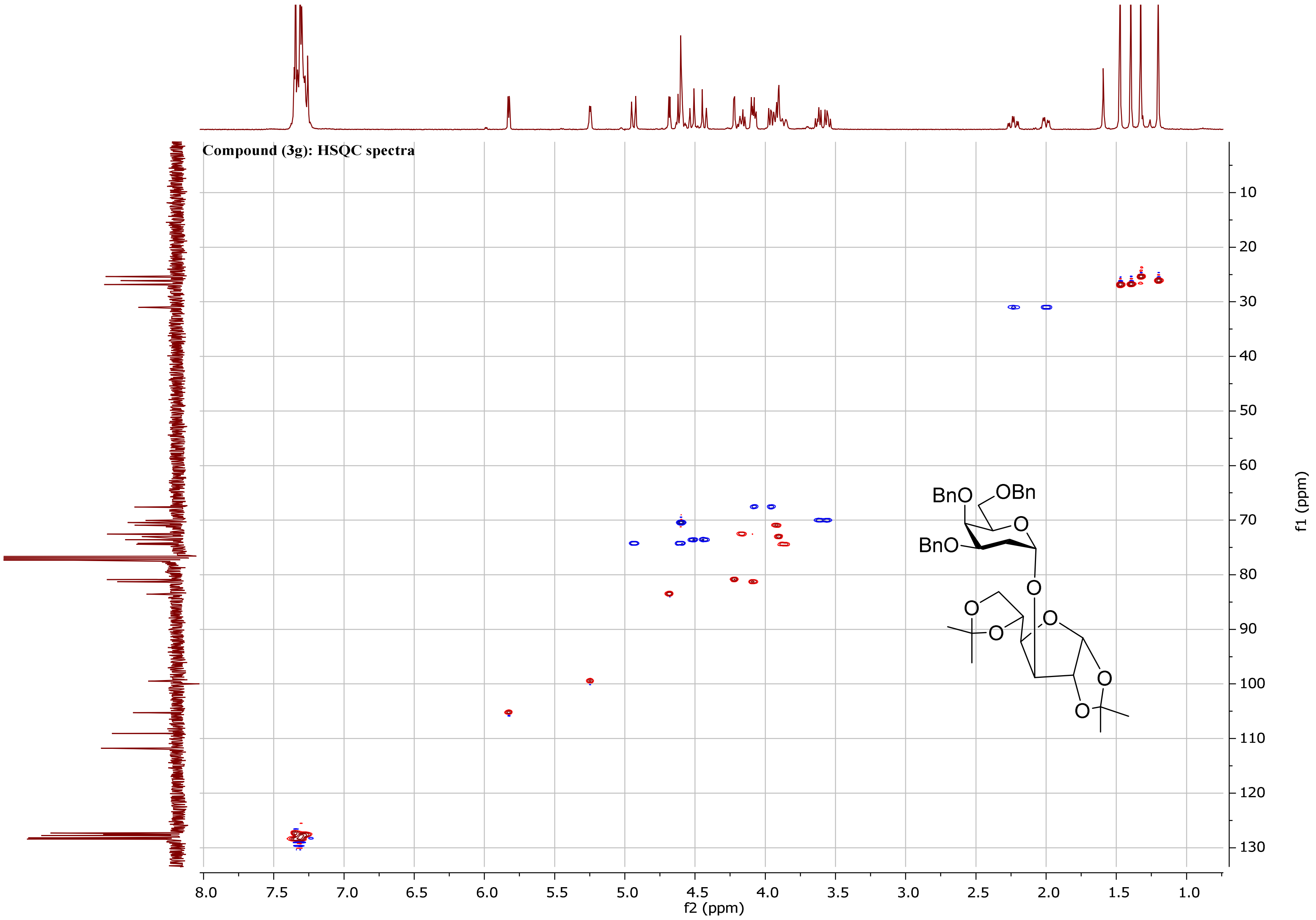
Compound (3g): HSQC spectra
BnO
OBn
O
BnO
O
O
O
O
O
O
f2 (ppm)
f1 (ppm)

Compound (3h): Proton NMR (500 MHz, Chloroform-d)

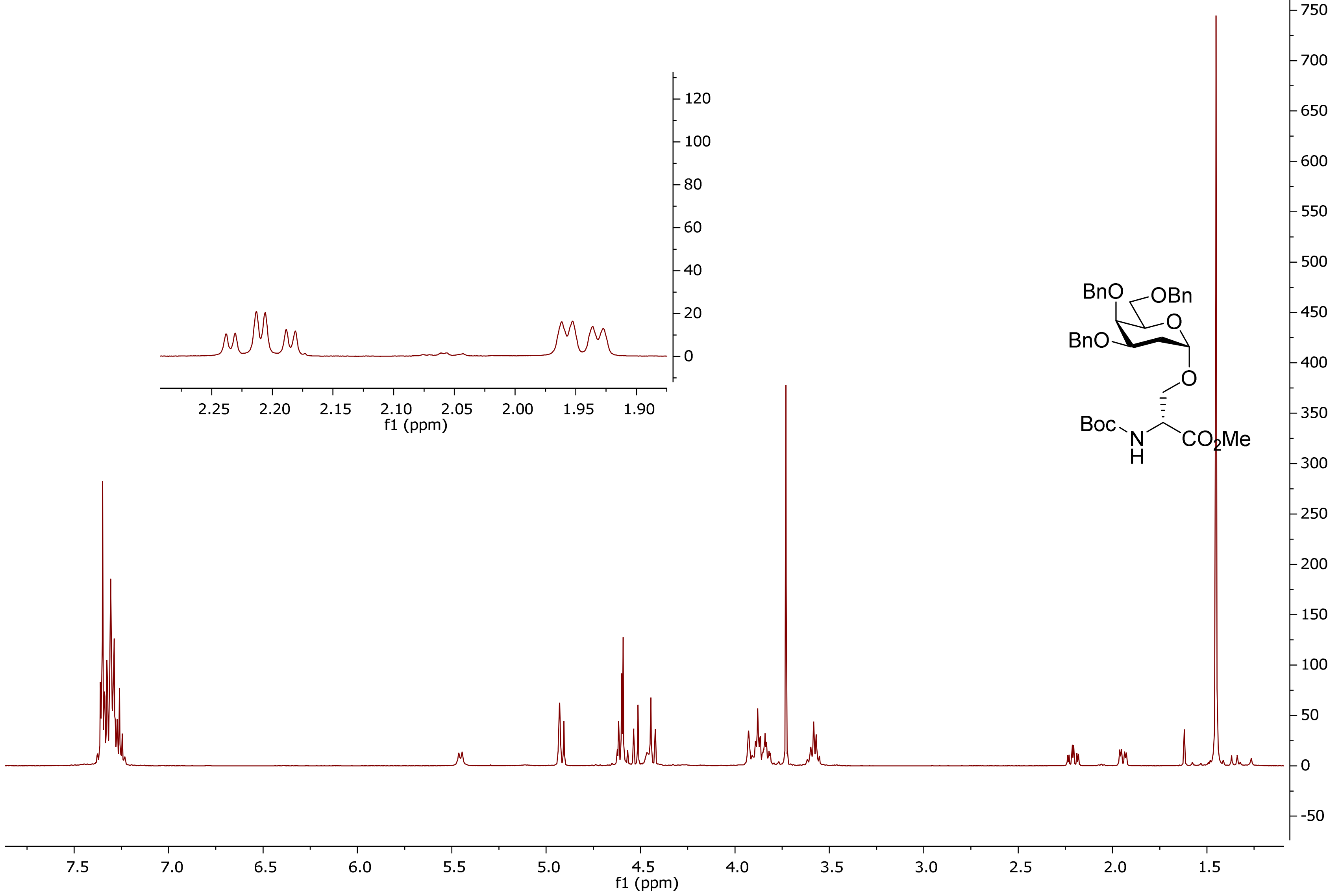

Compound (3h): Carbon-13 NMR (126 MHz, Chloroform-d)

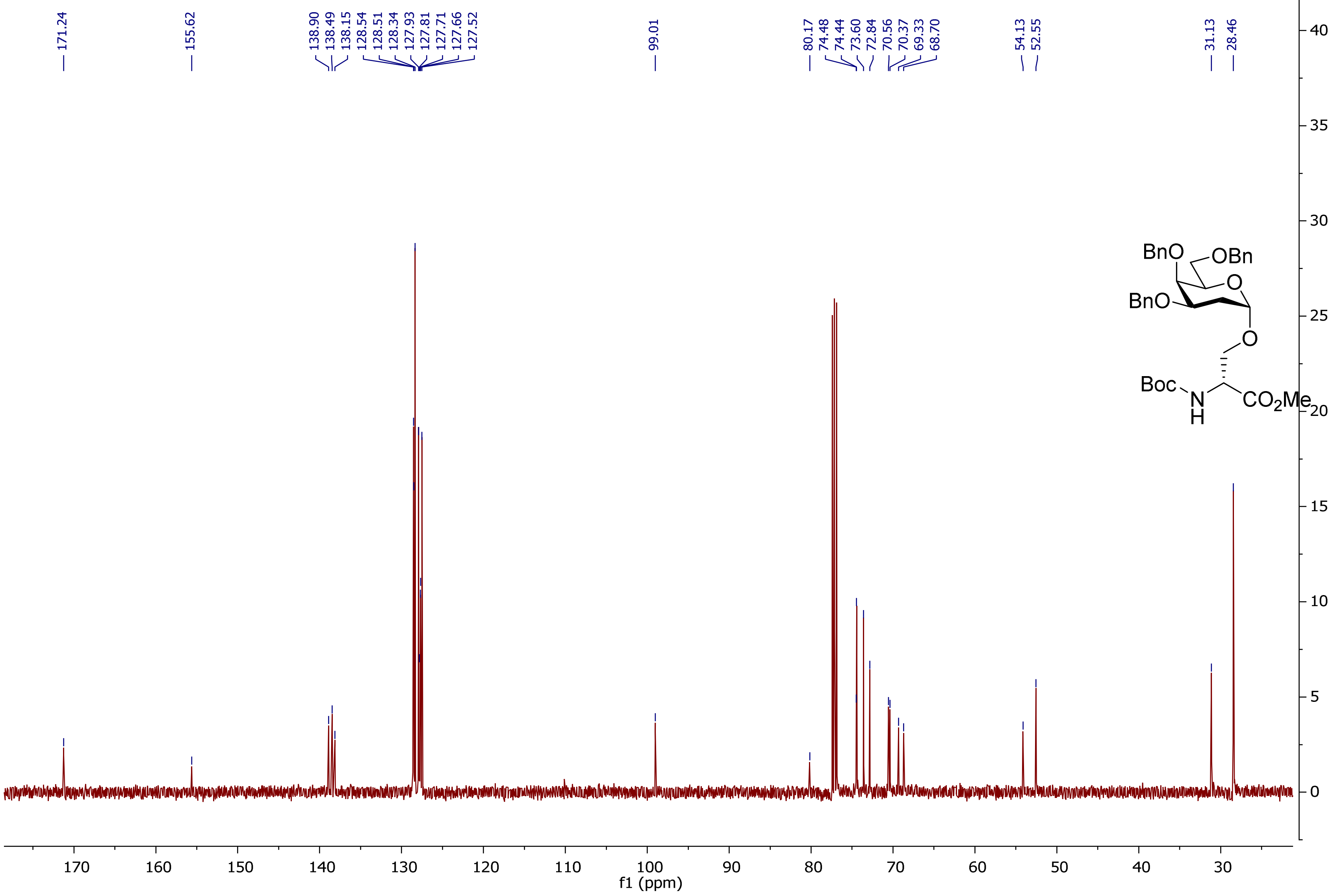

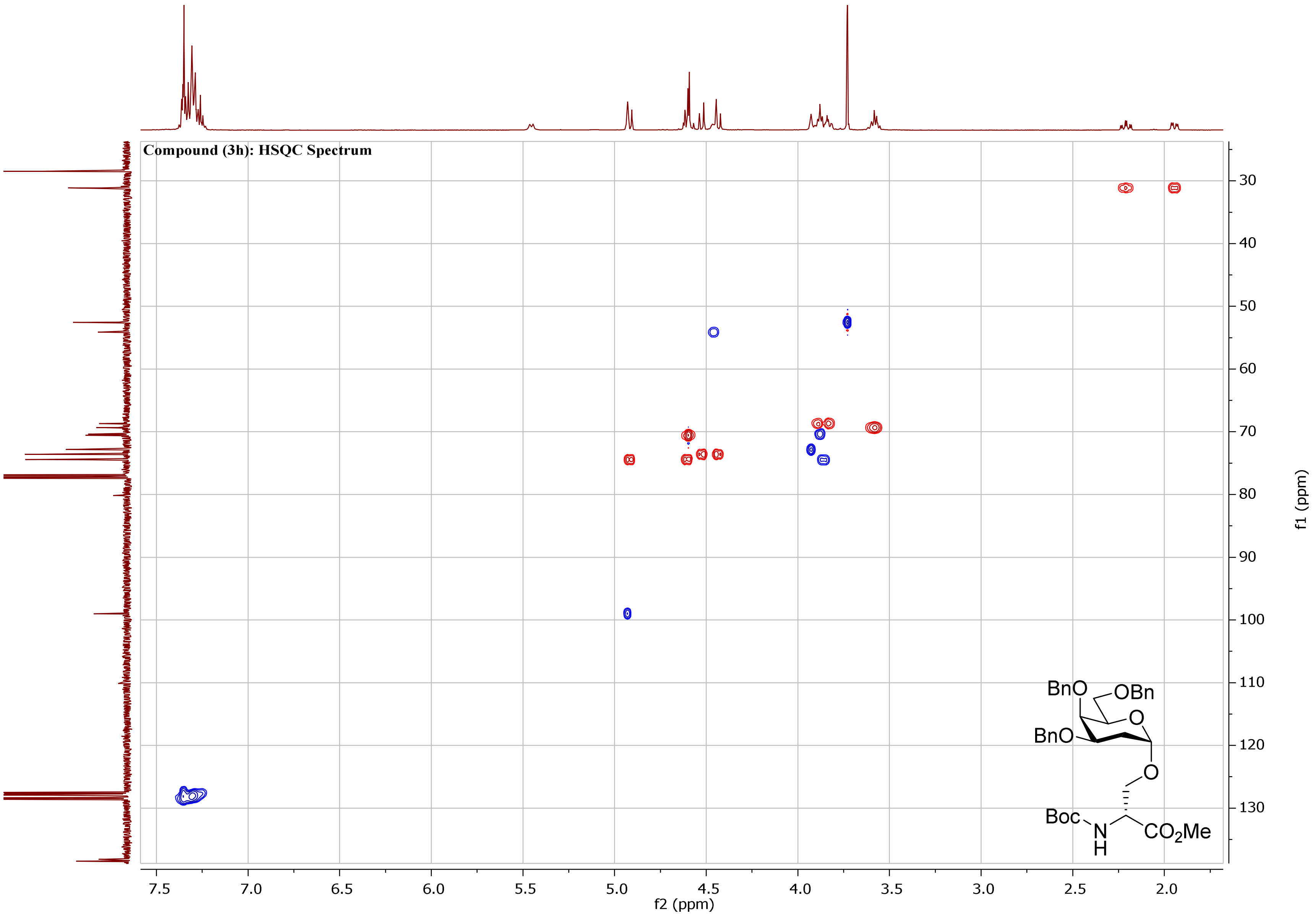
Compound (3h): HSQC Spectrum
BnO
OBn
O
BnO
O
Boc
N
H
CO2Me
f2 (ppm)
f1 (ppm)
7.5
7.0
6.5
6.0
5.5
5.0
4.5
4.0
3.5
3.0
2.5
2.0
30
40
50
60
70
80
90
100
110
120
130

Compound (3i): Proton NMR (400 MHz, Chloroform-d)

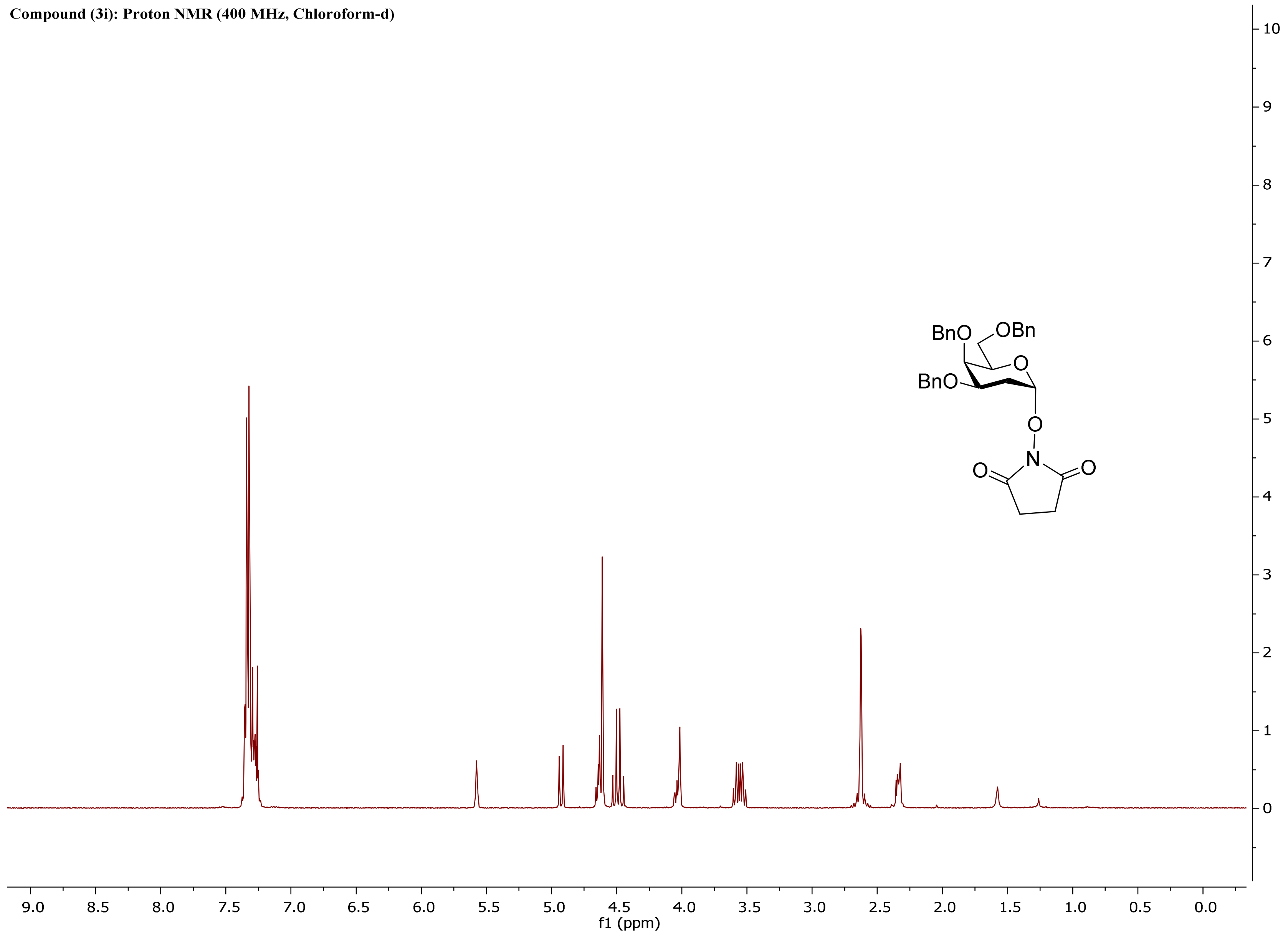

Compound (3i): Carbon NMR (101 MHz, Chloroform-d)

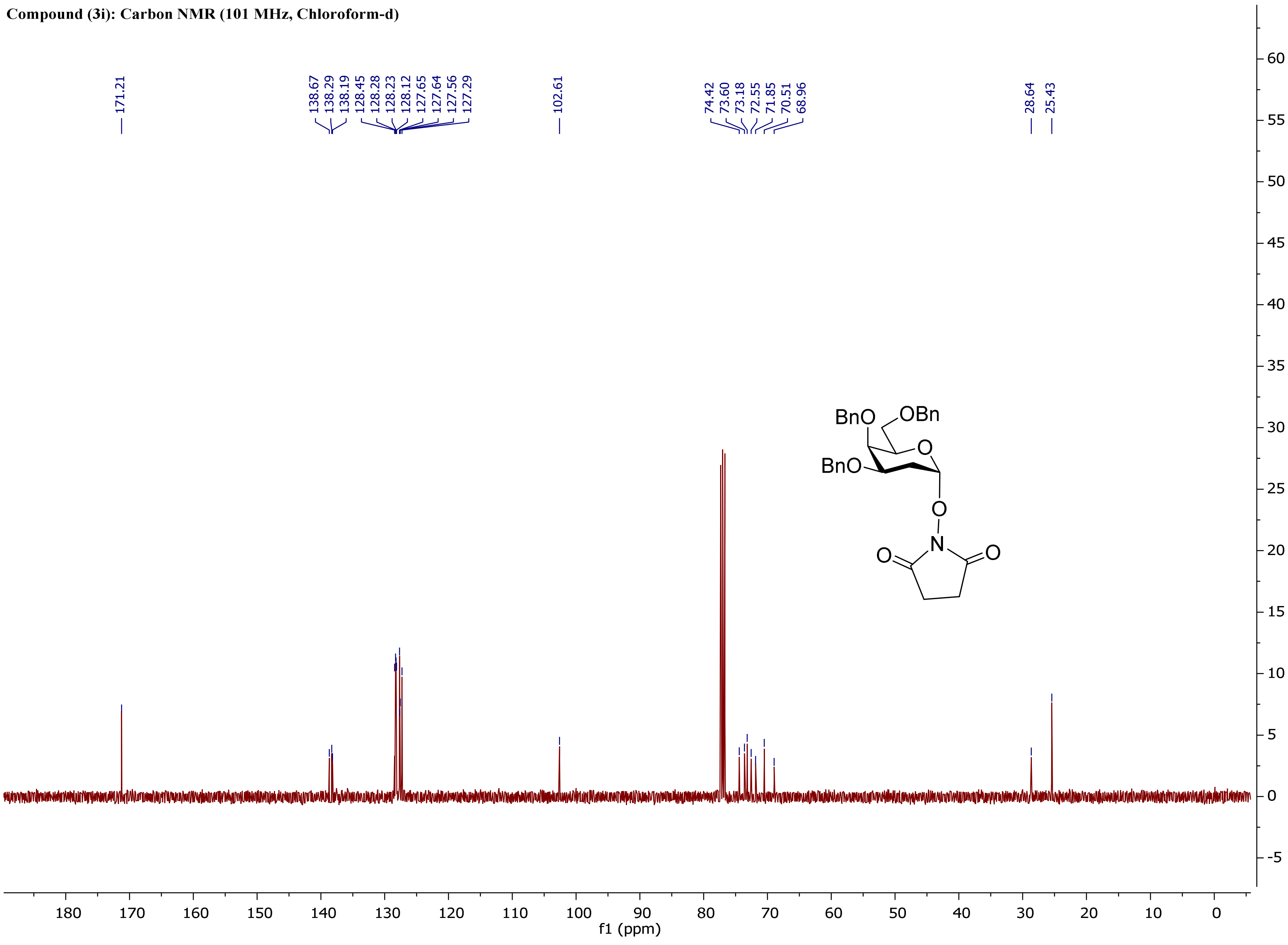

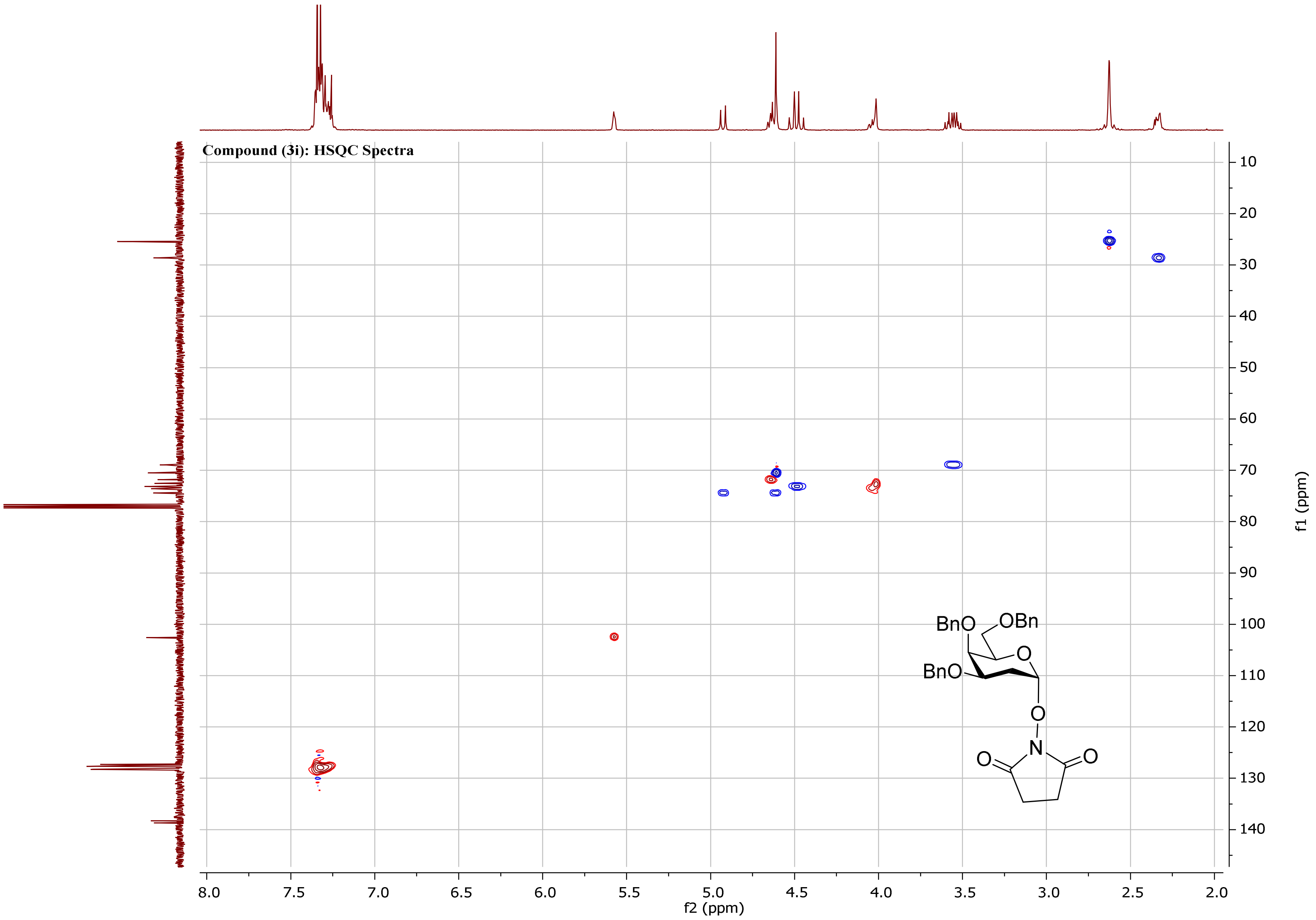
Compound (3i): HSQC Spectra
f2 (ppm)
f1 (ppm)
BnO
OBn
O
BnO
O
N
O
O

Compound (3j): Proton NMR (500 MHz, Chloroform-d)

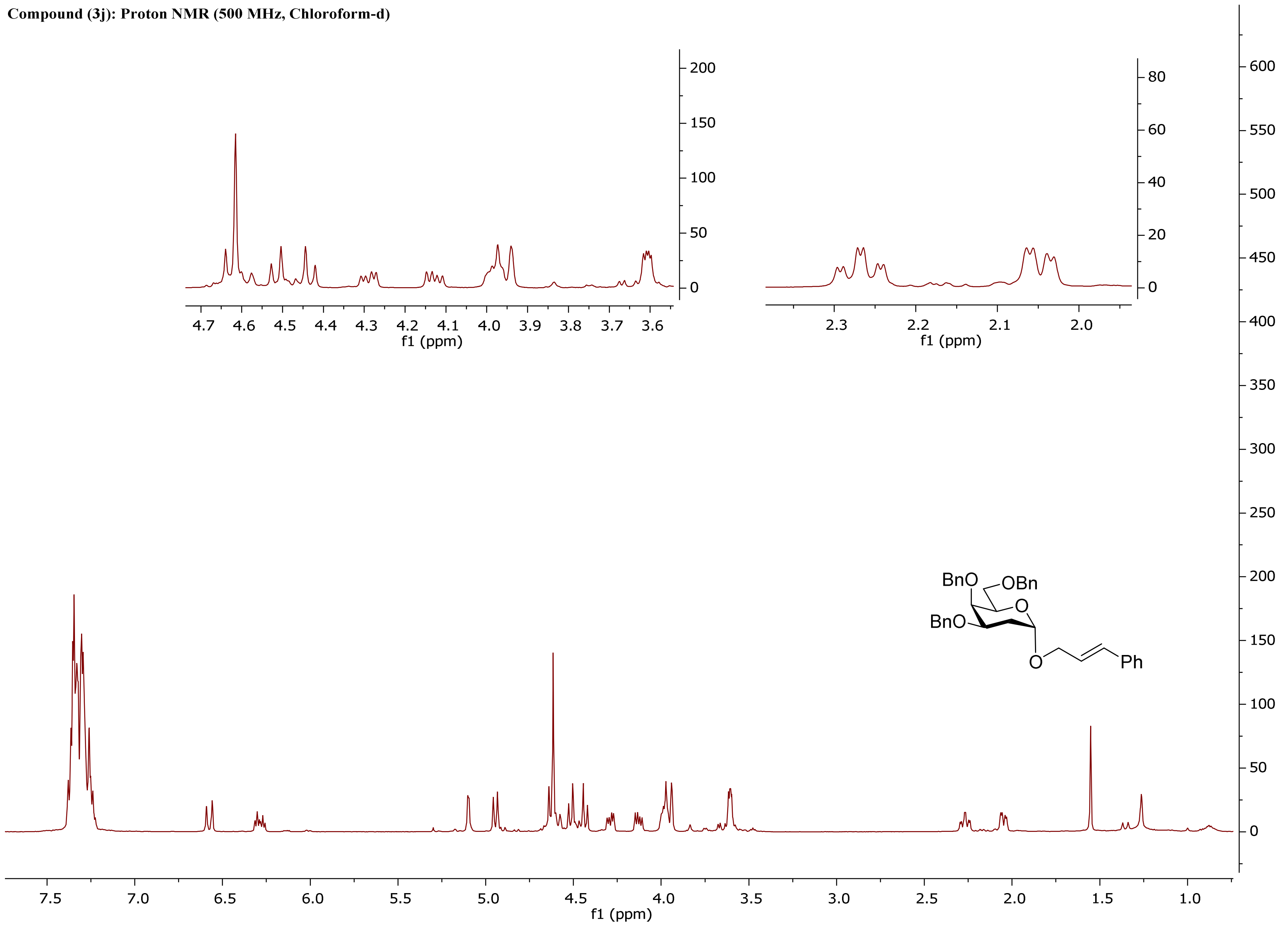

Compound (3j): Carbon-13 NMR (126 MHz, Chloroform-d)

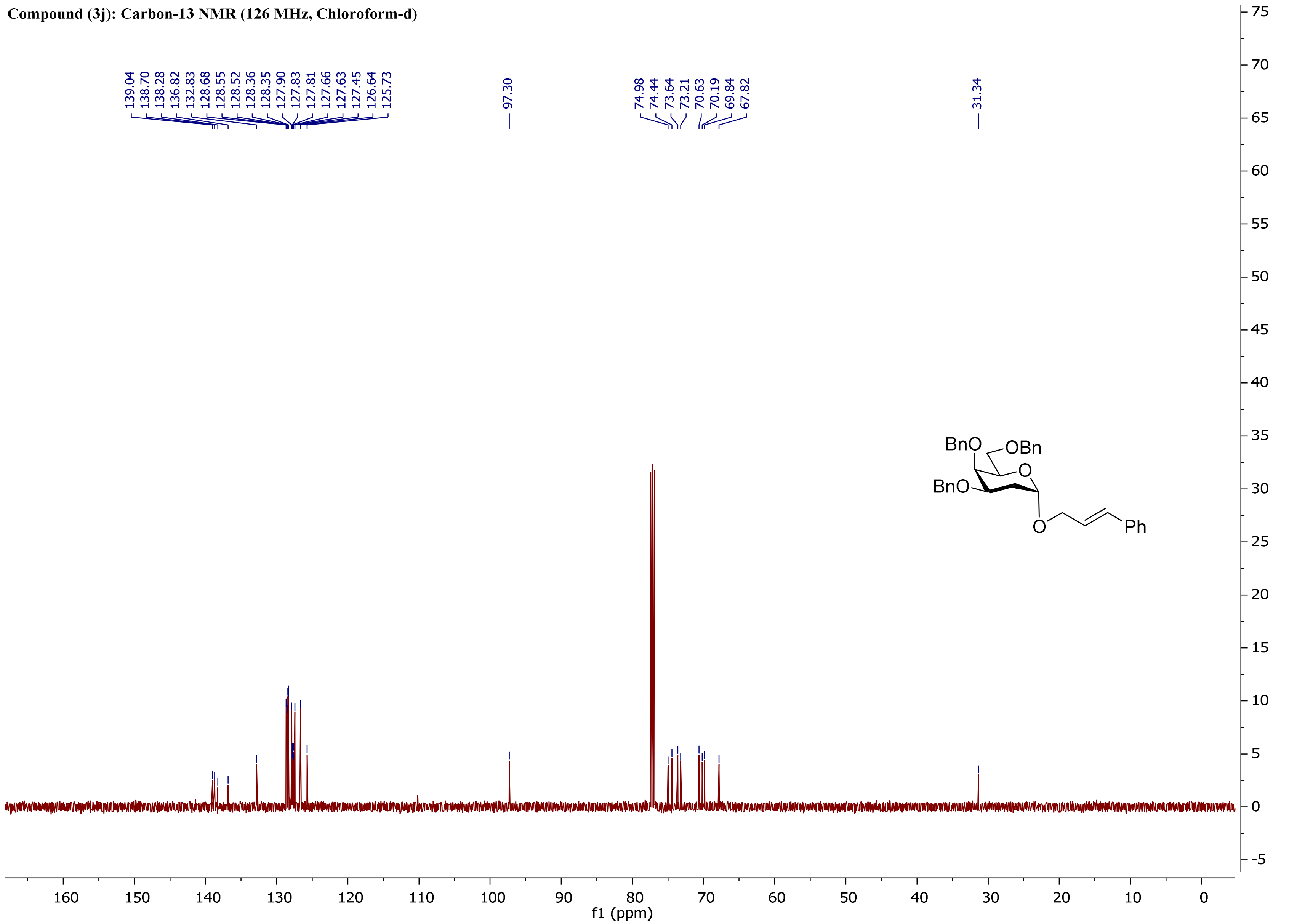

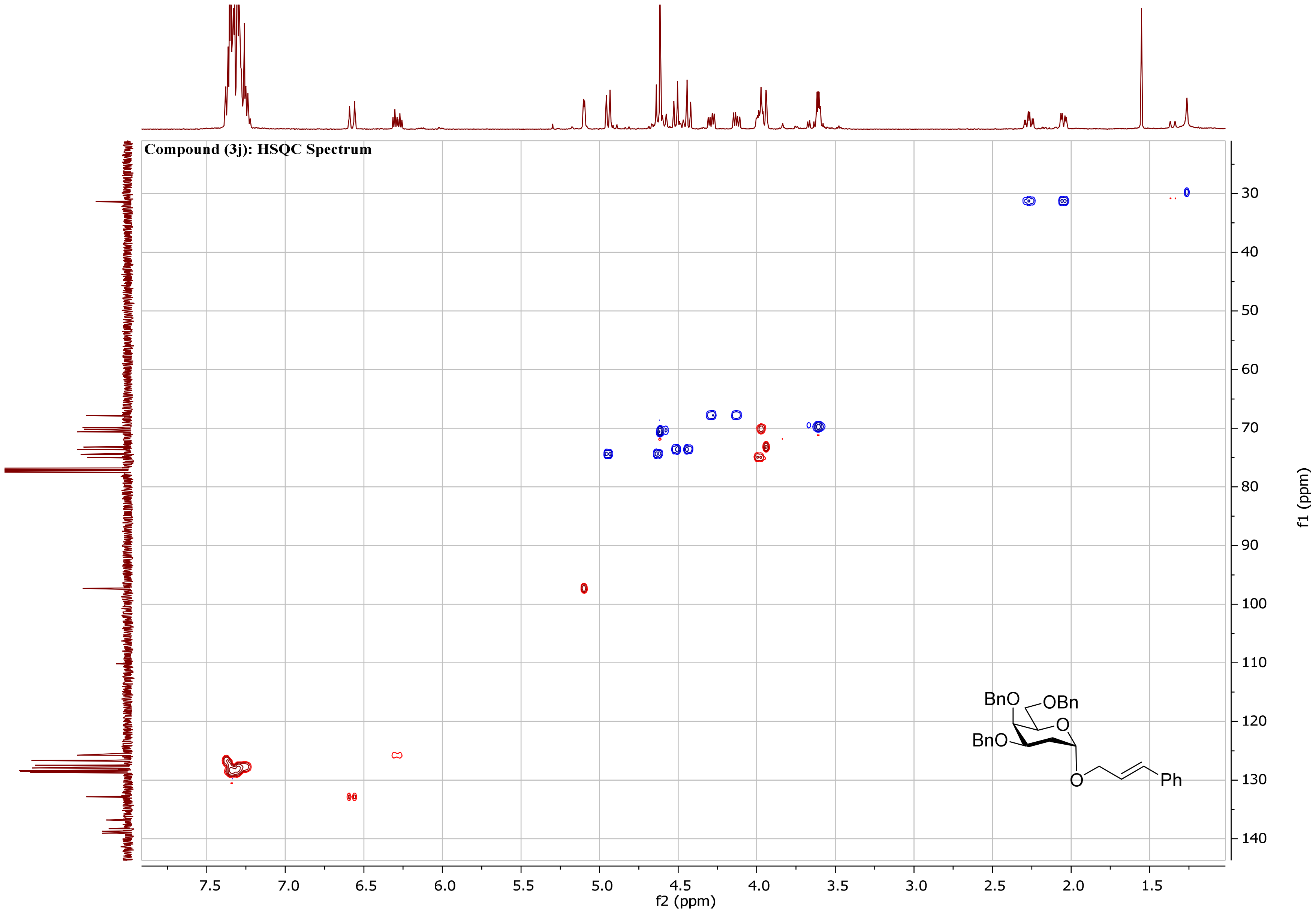
Compound (3j): HSQC Spectrum
BnO
OBn
O
BnO
O
Ph
f2 (ppm)
f1 (ppm)

Compound (6b) Proton NMR (400 MHz, Chloroform-d)

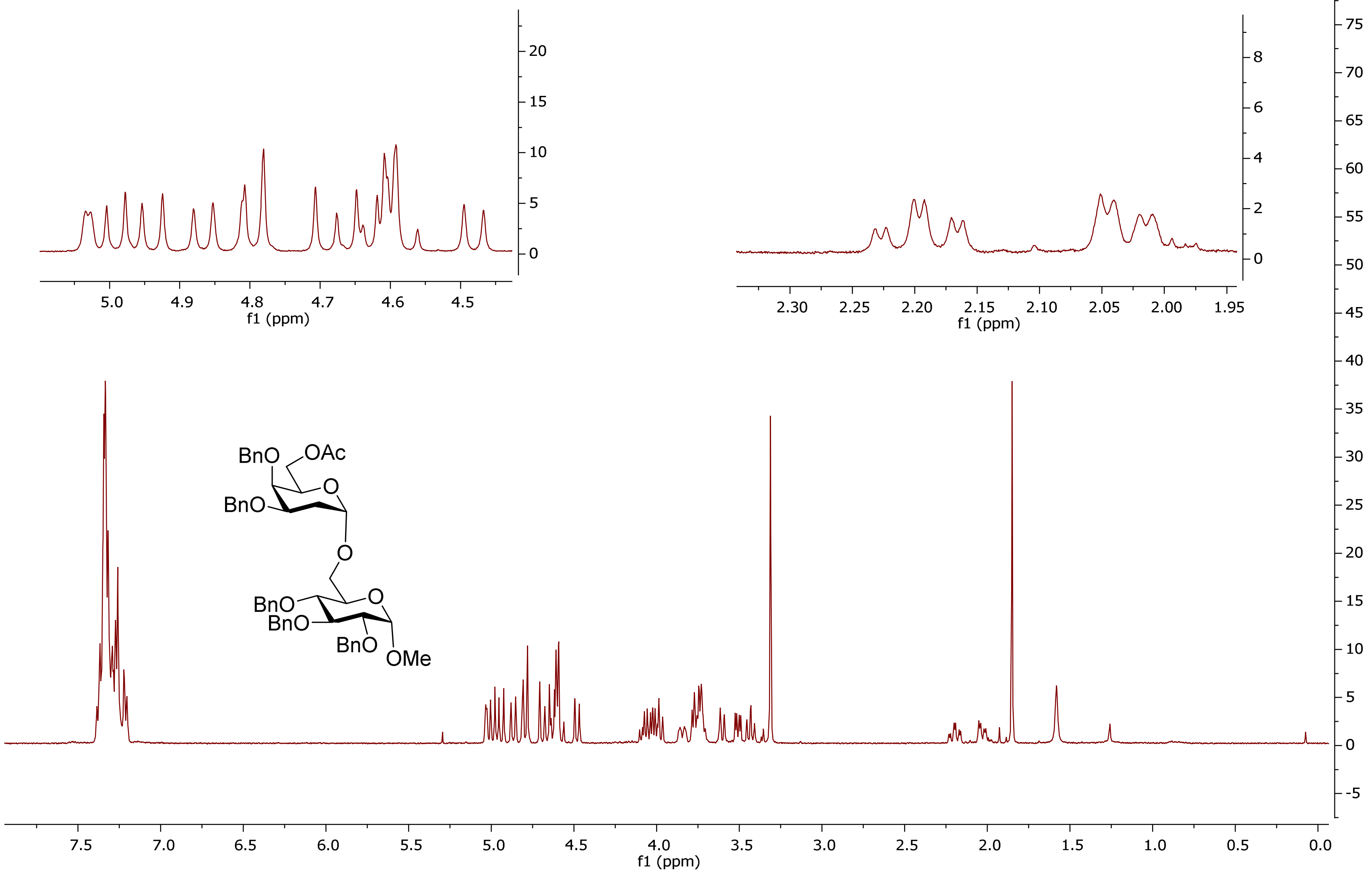

Compound (6b) Carbon NMR (101 MHz, Chloroform-d)

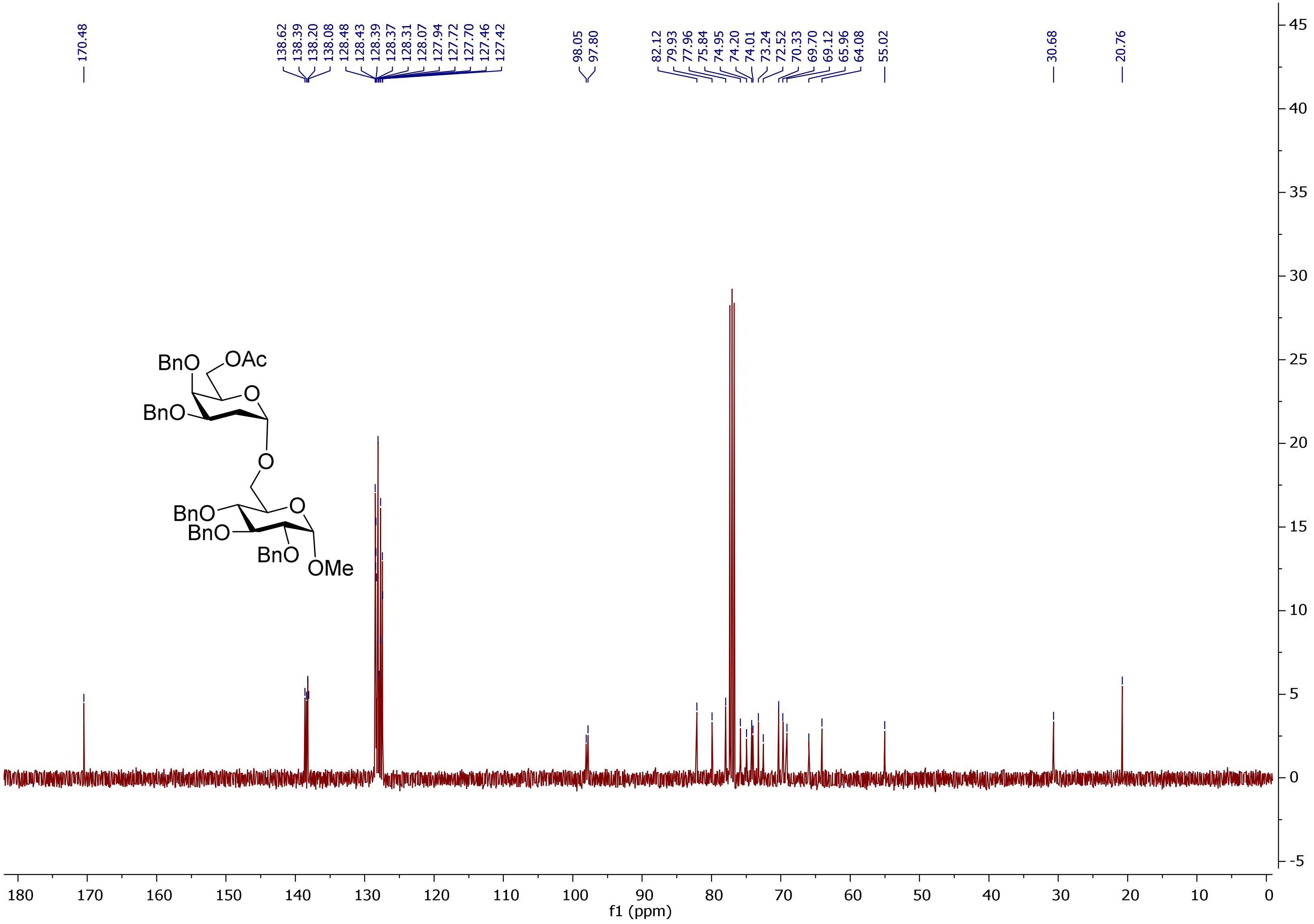

Compound (6b): HSQC Spectra

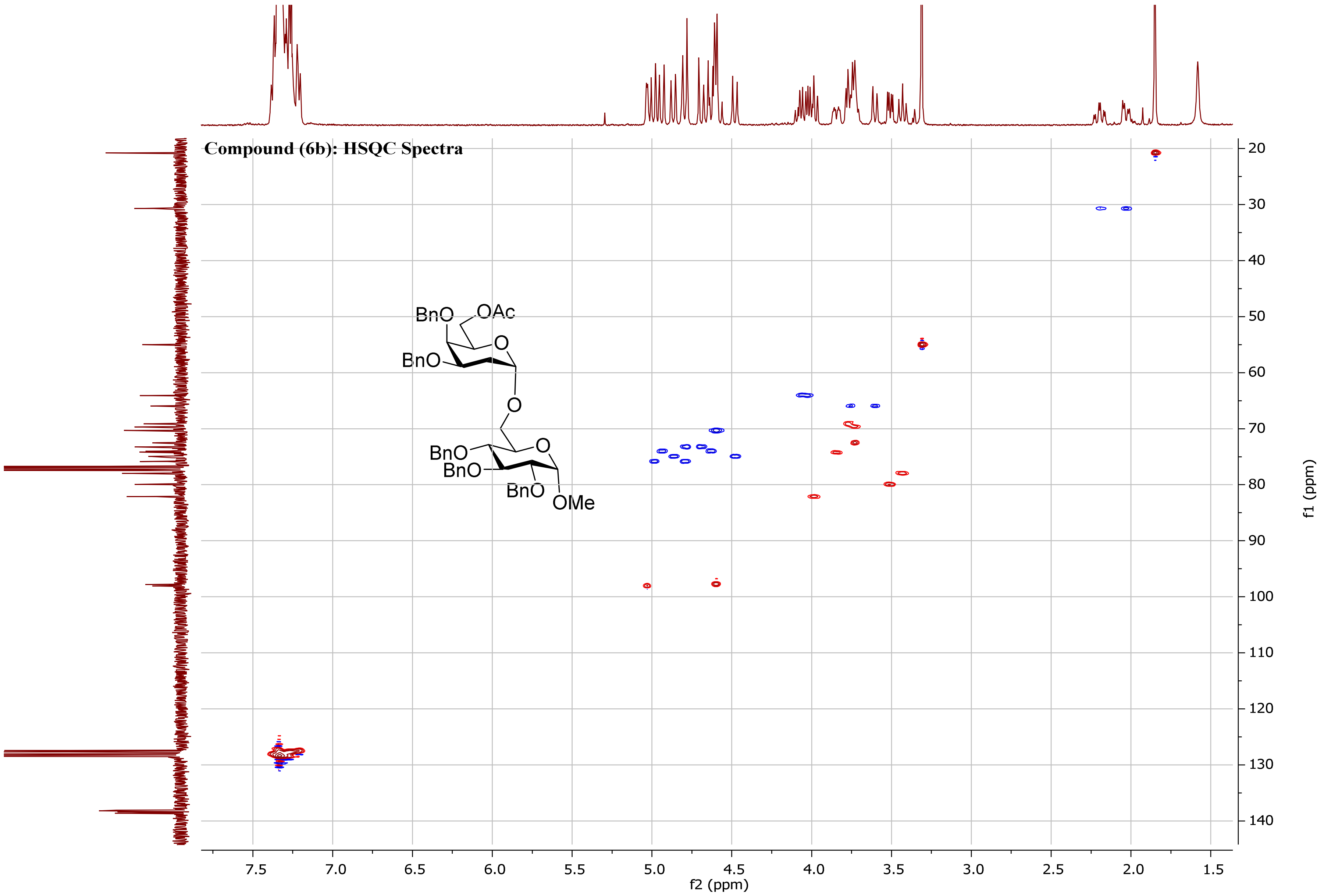

Compound (6c) Proton NMR (400 MHz, Chloroform-d)

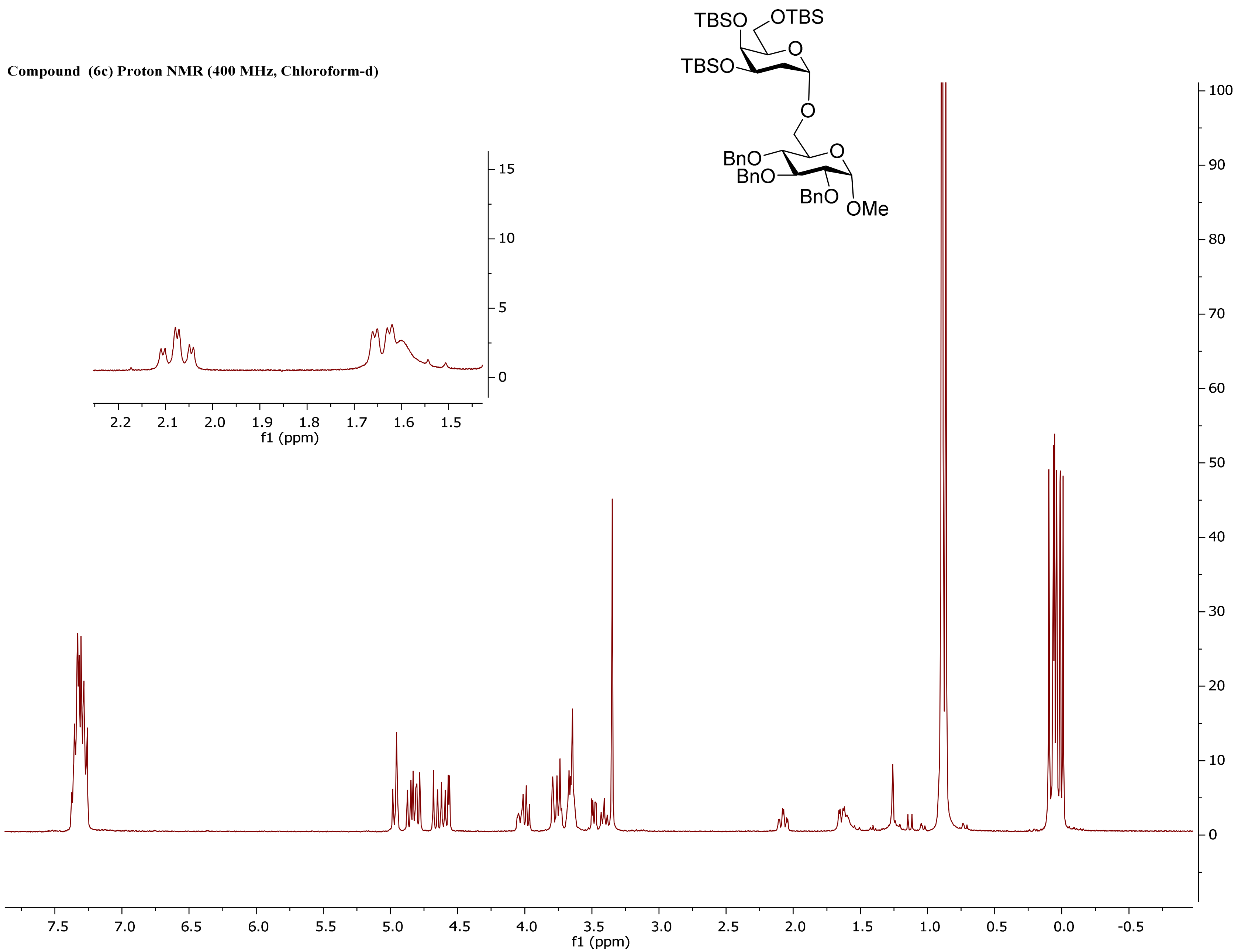

Compound (6c) Carbon NMR (101 MHz, Chloroform-d)

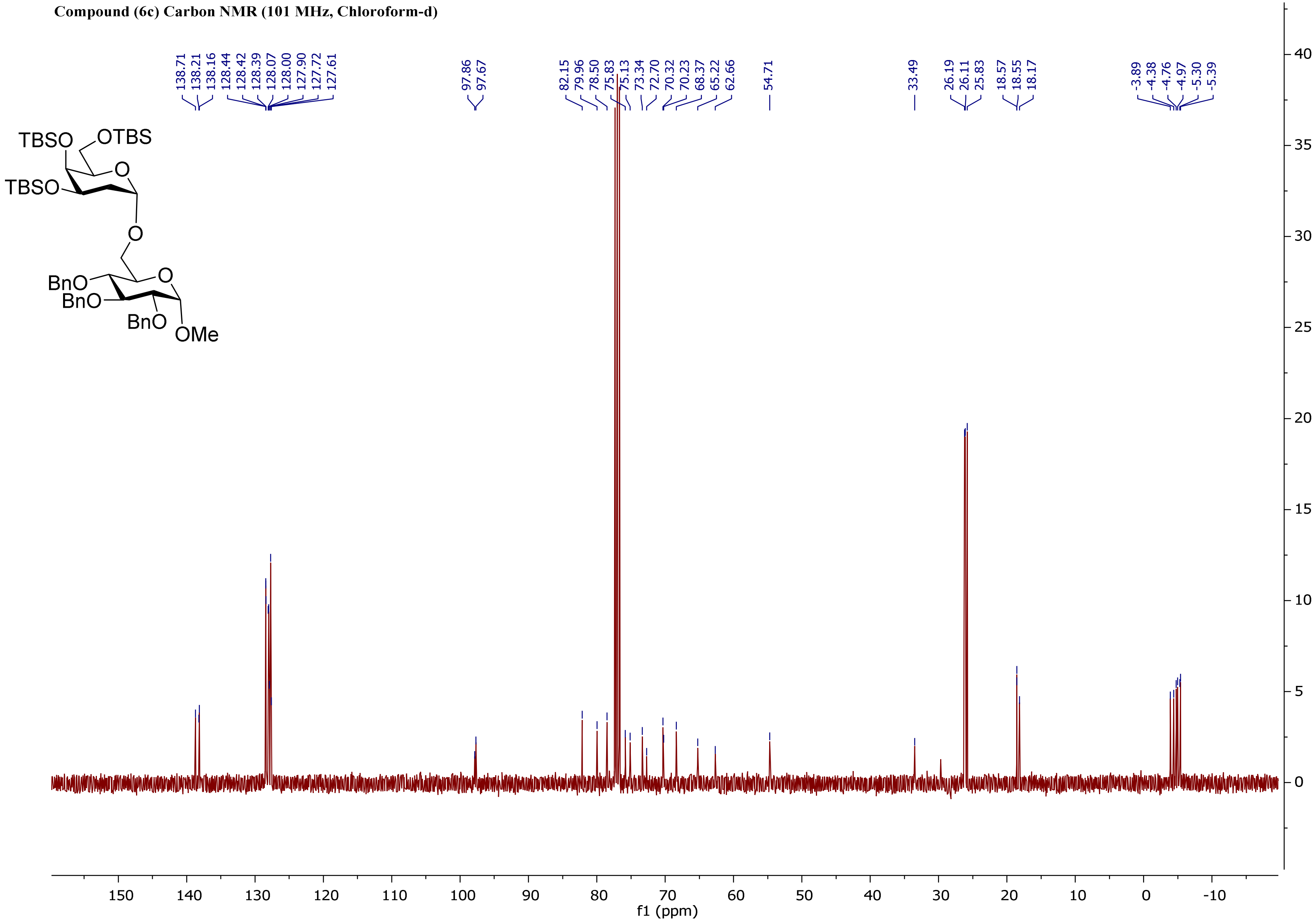

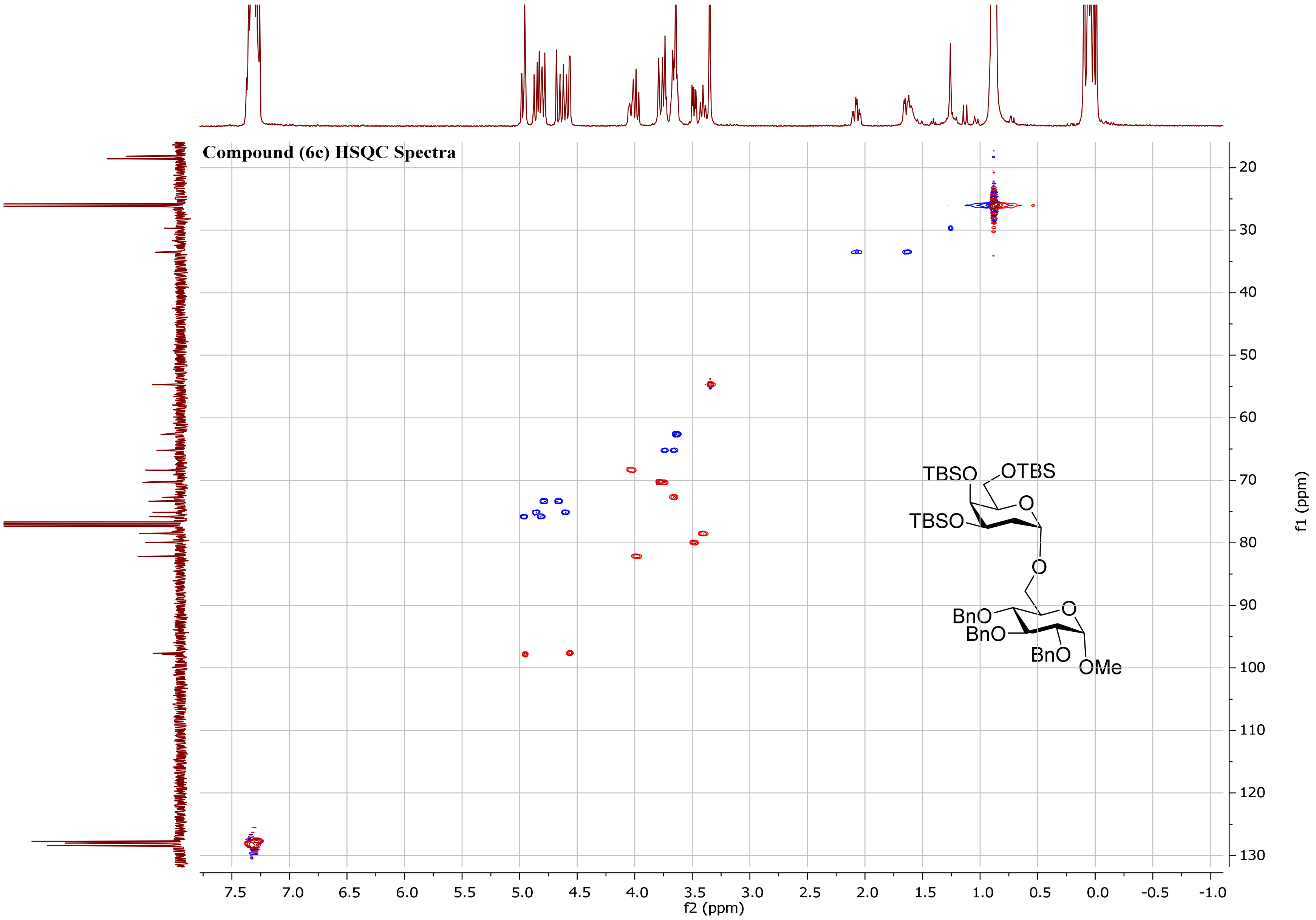
Compound (6c) HSQC Spectra
TBSO
OTBS
TBSO
O
O
BnO
BnO
BnO
O
OMe
f2 (ppm)
f1 (ppm)

Compound (6d) Proton NMR (400 MHz, Chloroform-d)

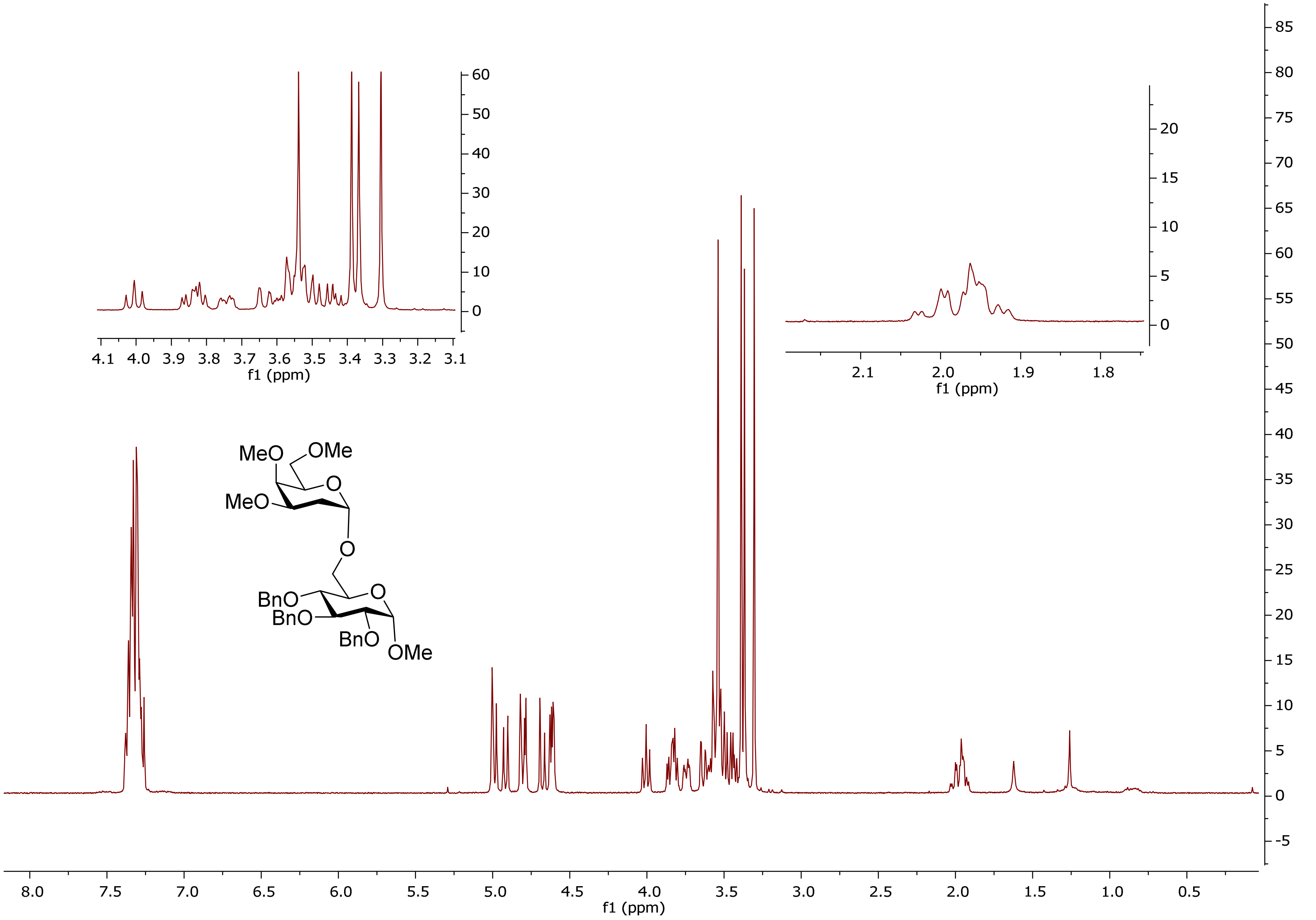

Compound (6d) Carbon NMR (101 MHz, Chloroform-d)

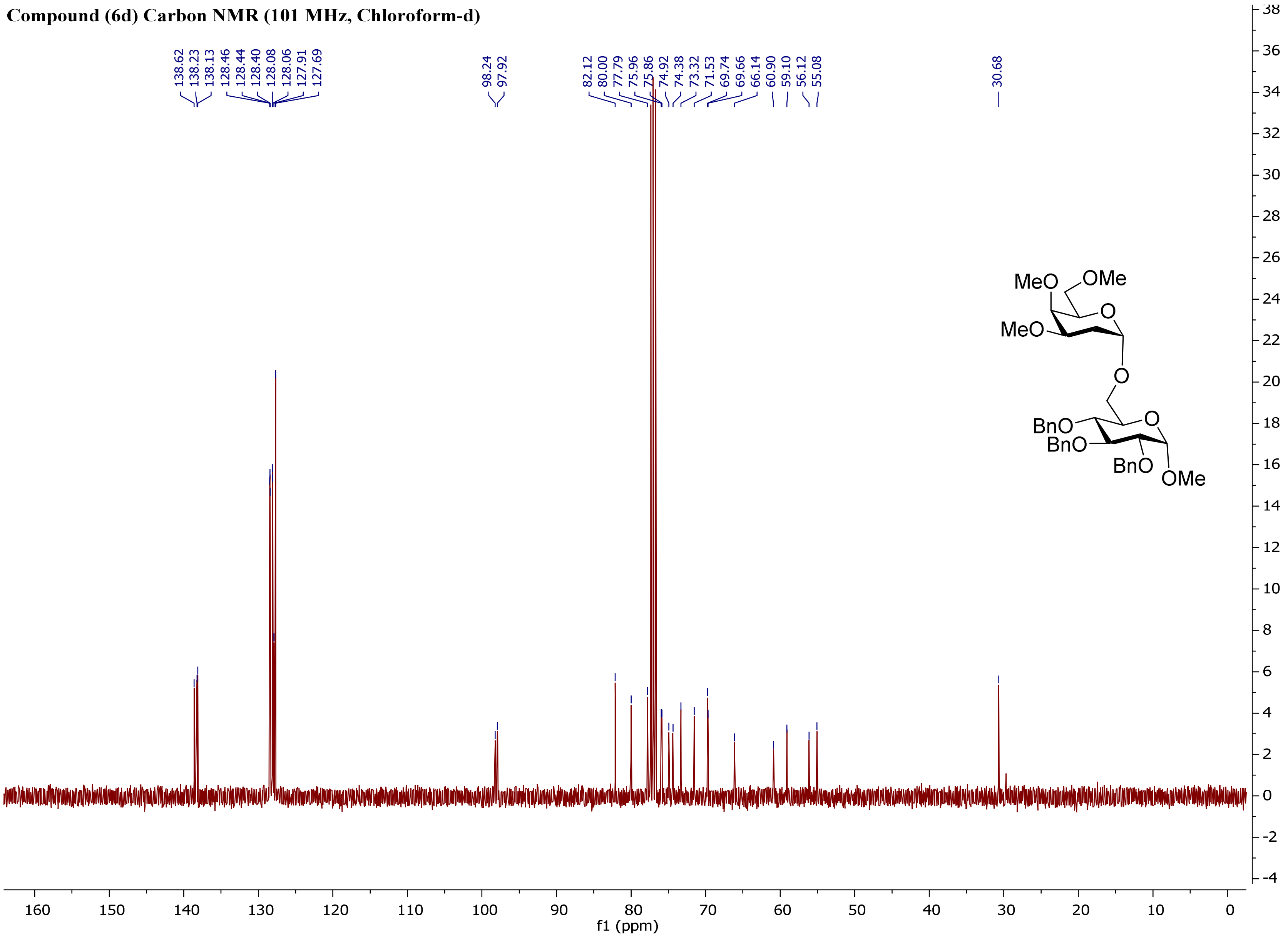

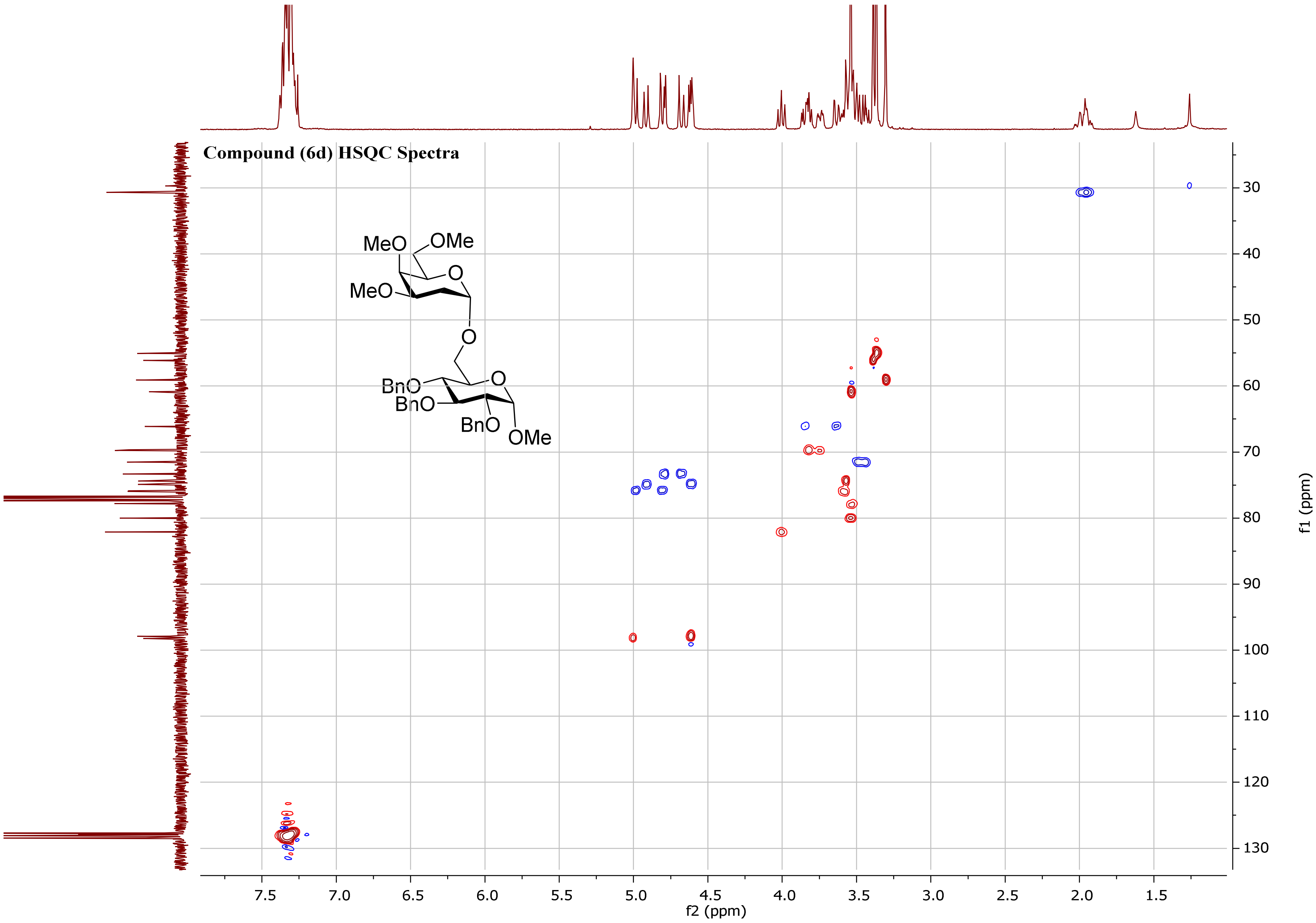
Compound (6d) HSQC Spectra
MeO
OMe
O
MeO
O
BnO
BnO
O
BnO
OMe
f2 (ppm)
f1 (ppm)

Compound (6f) Proton NMR (500 MHz, Chloroform-d)

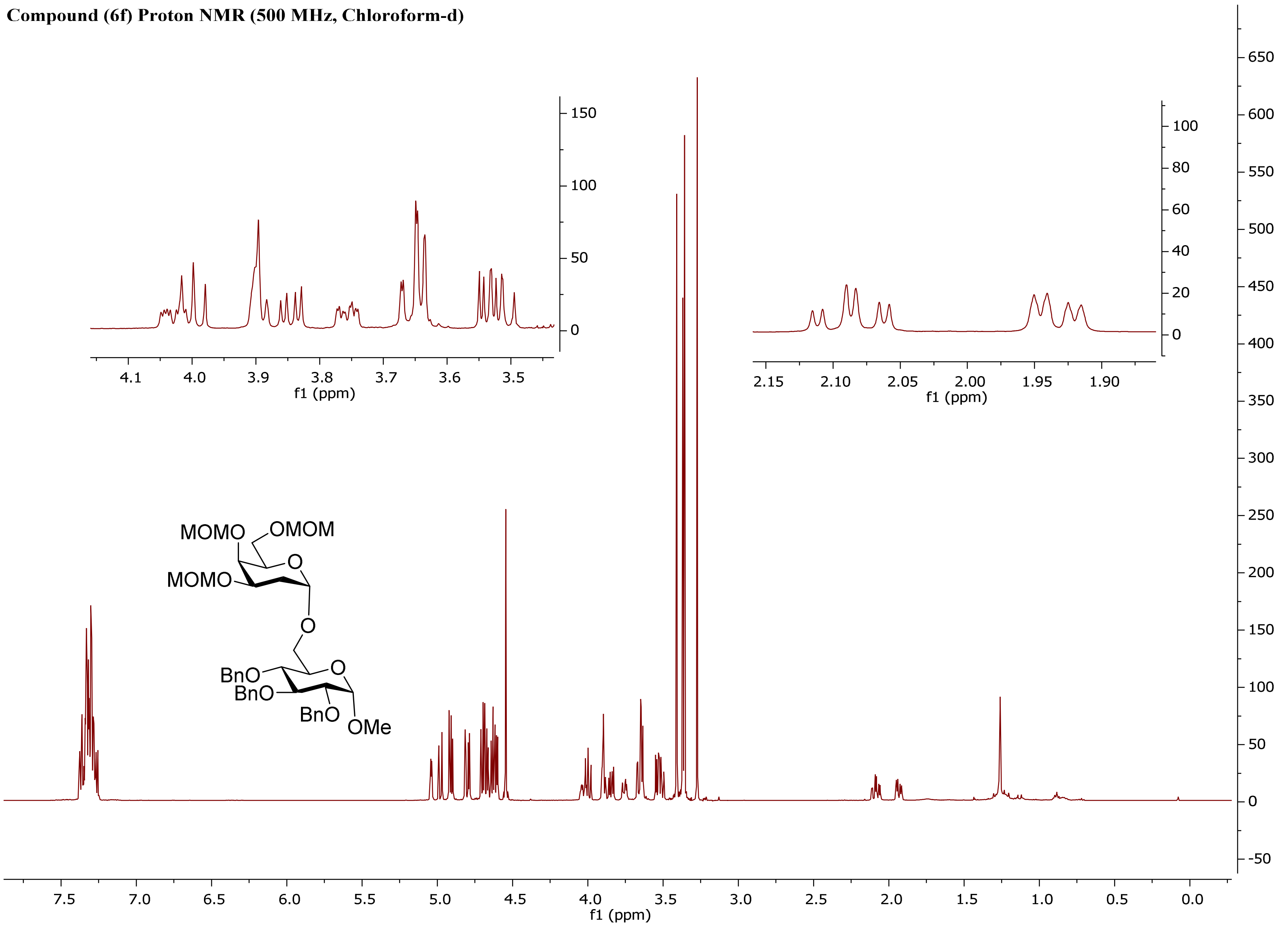

Compound (6f) Carbon NMR (101 MHz, Chloroform-d)

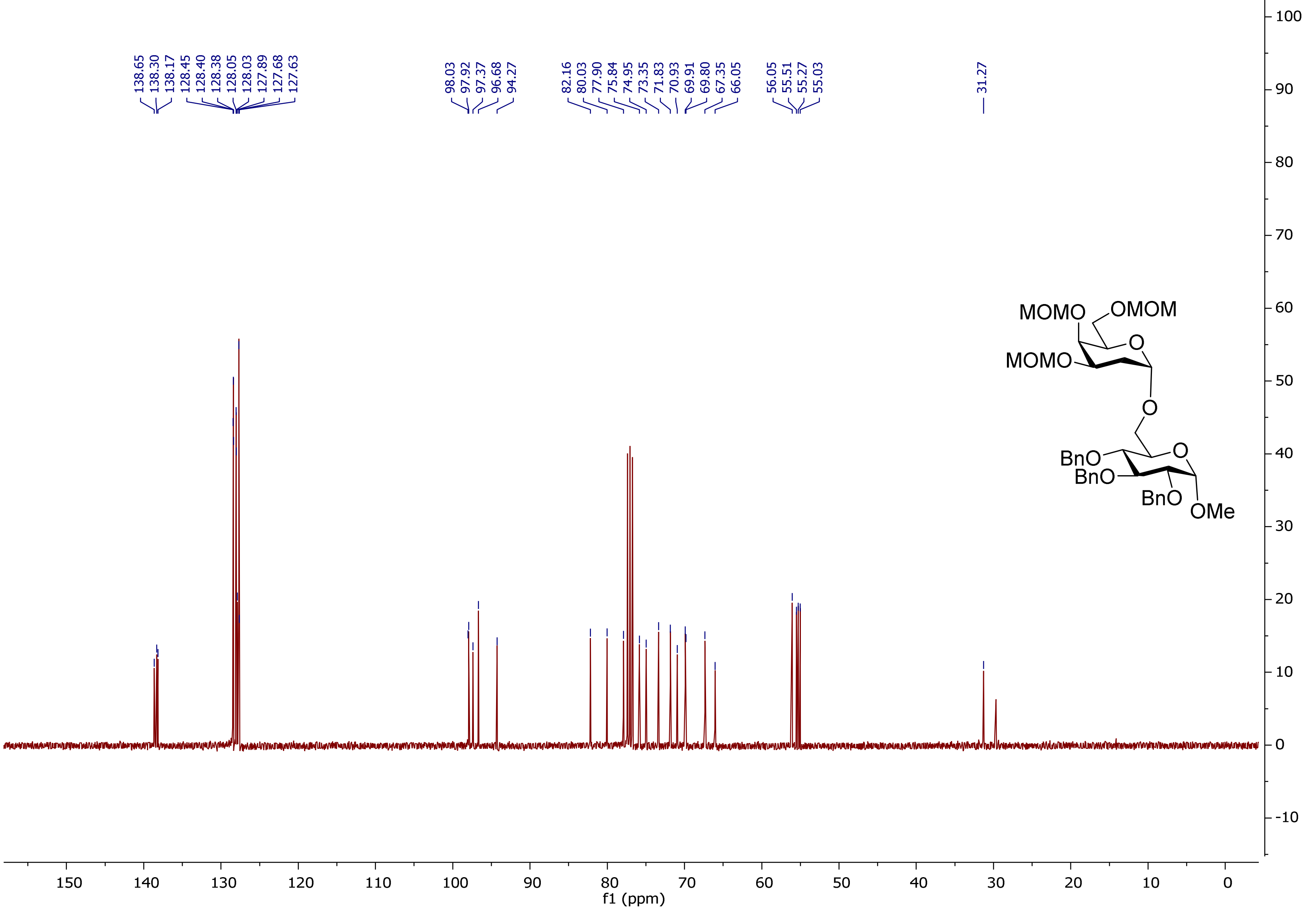

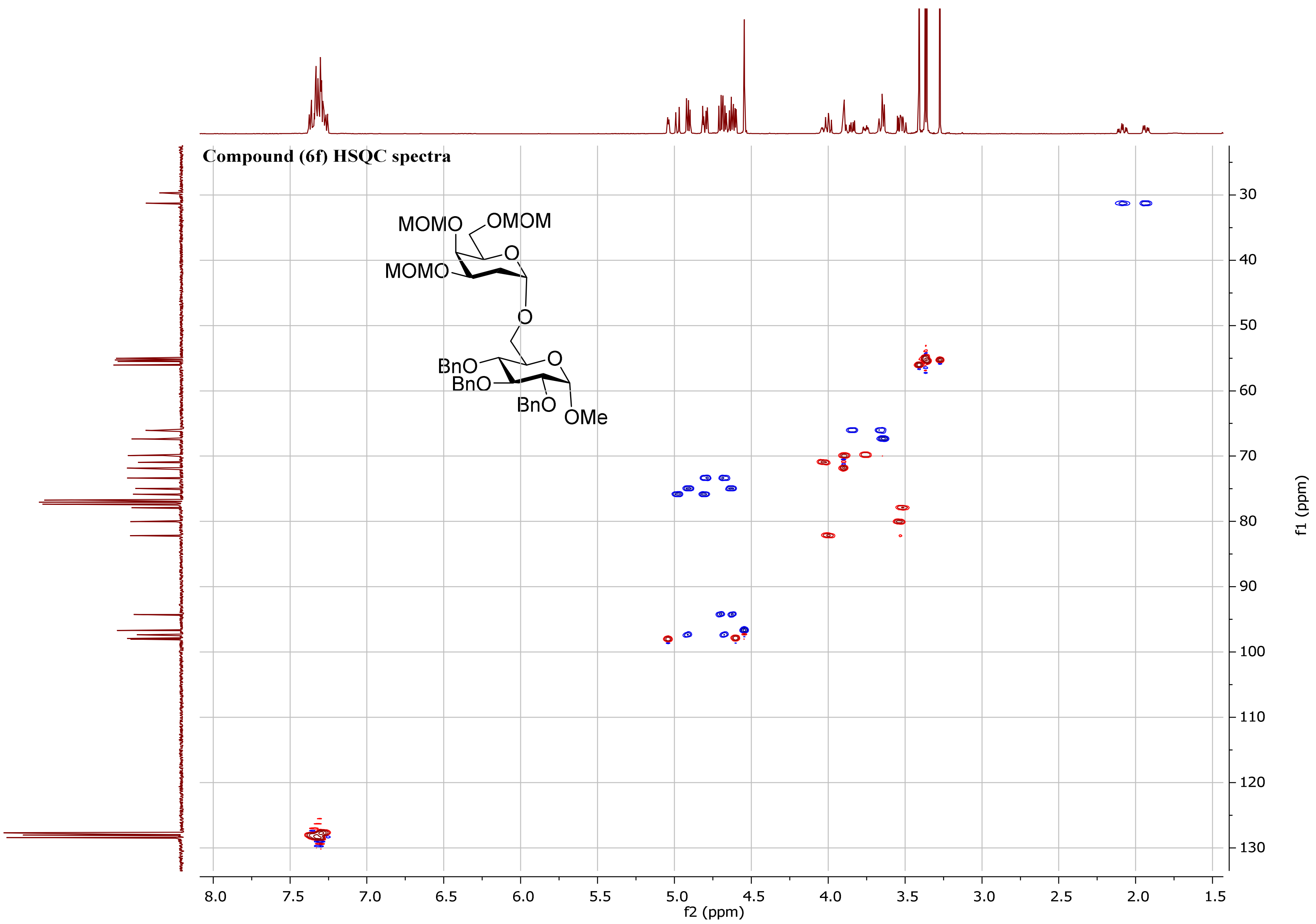
Compound (6f) HSQC spectra
MOMO
OMOM
O
MOMO
O
BnO
BnO
O
BnO
OMe
f2 (ppm)
f1 (ppm)
8.0
7.5
7.0
6.5
6.0
5.5
5.0
4.5
4.0
3.5
3.0
2.5
2.0
1.5
30
40
50
60
70
80
90
100
110
120
130

Compound (6g) Proton NMR (500 MHz,Chloroform-d)

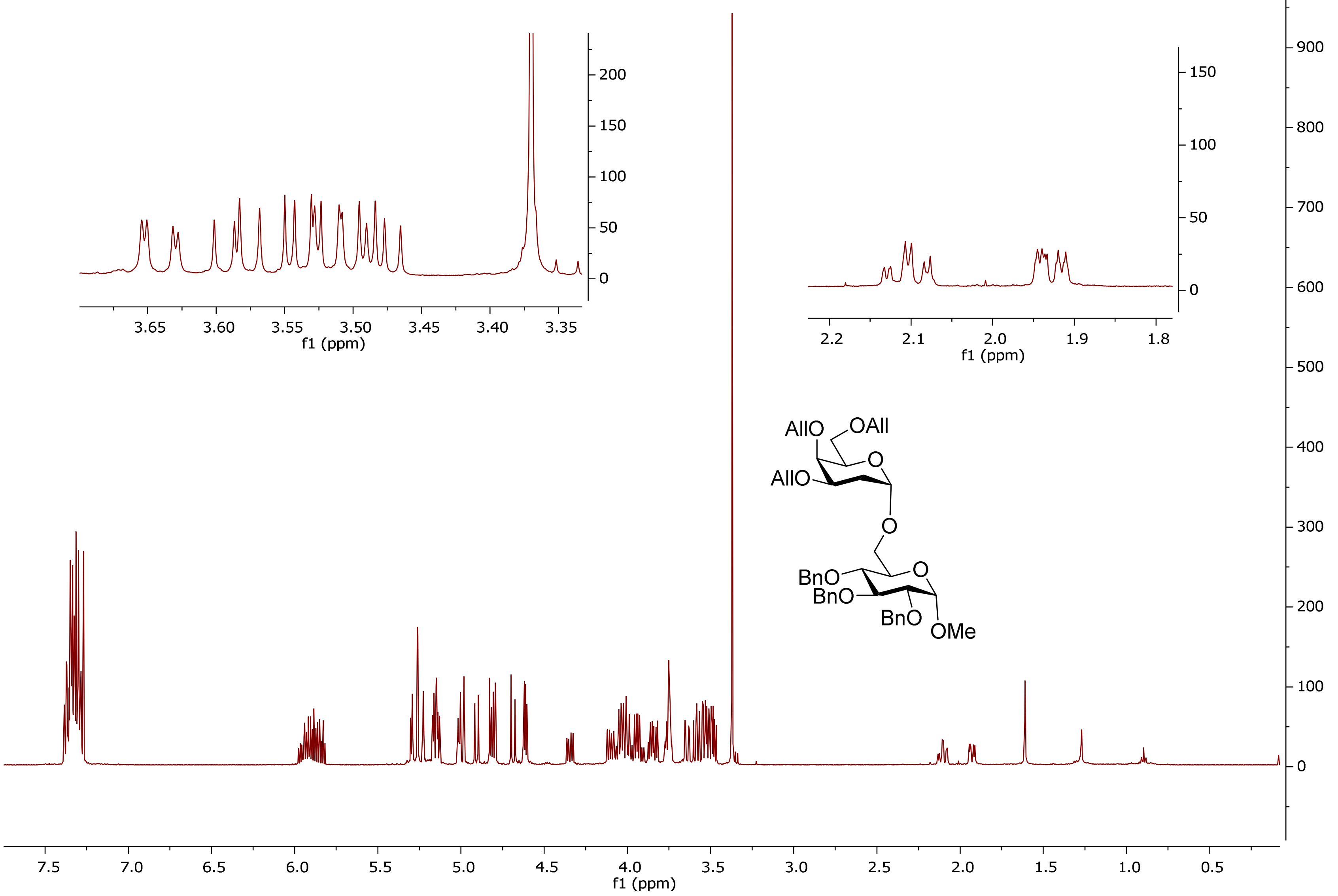

Compound (6g) Carbon NMR (101 MHz, Chloroform-d)

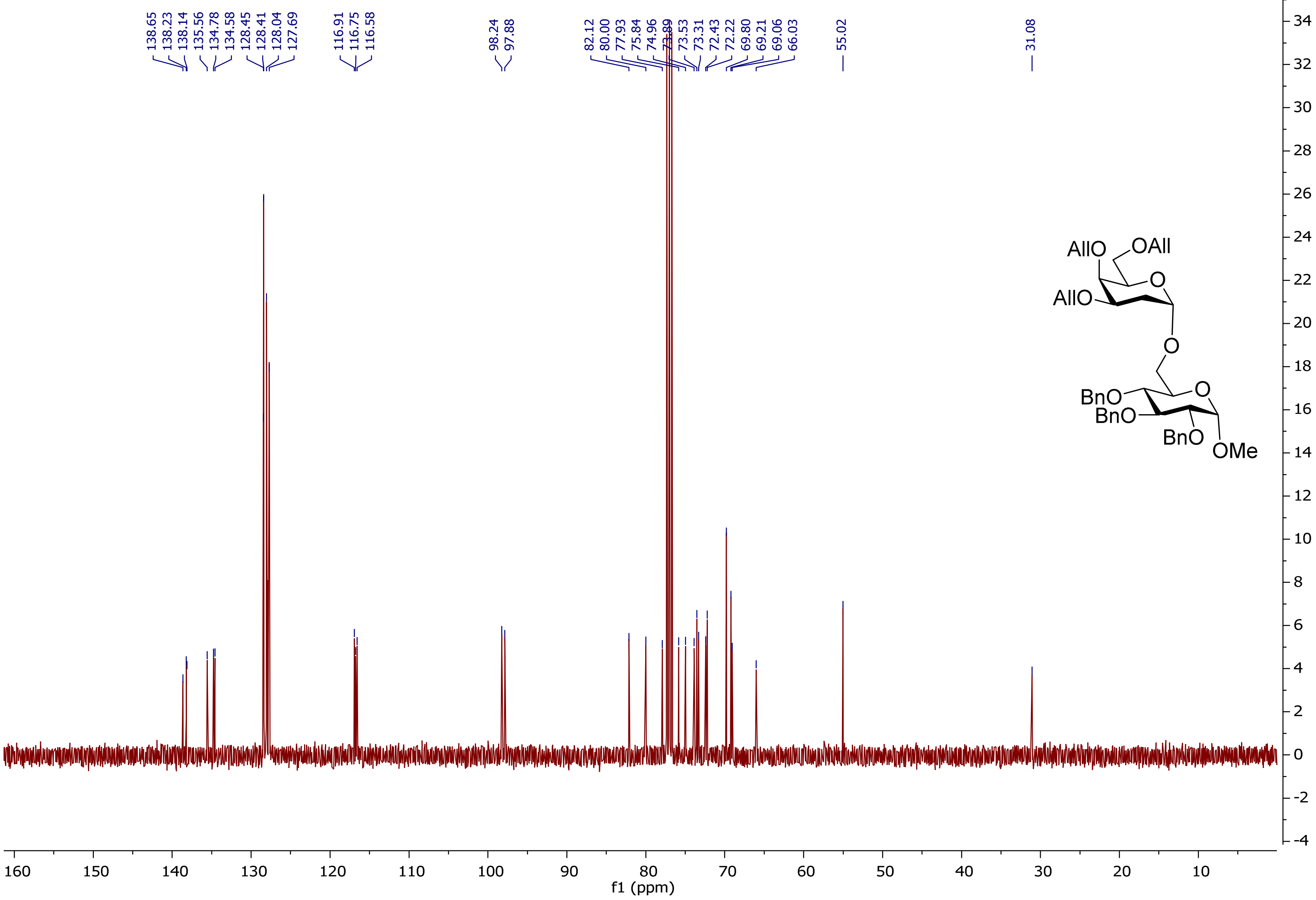

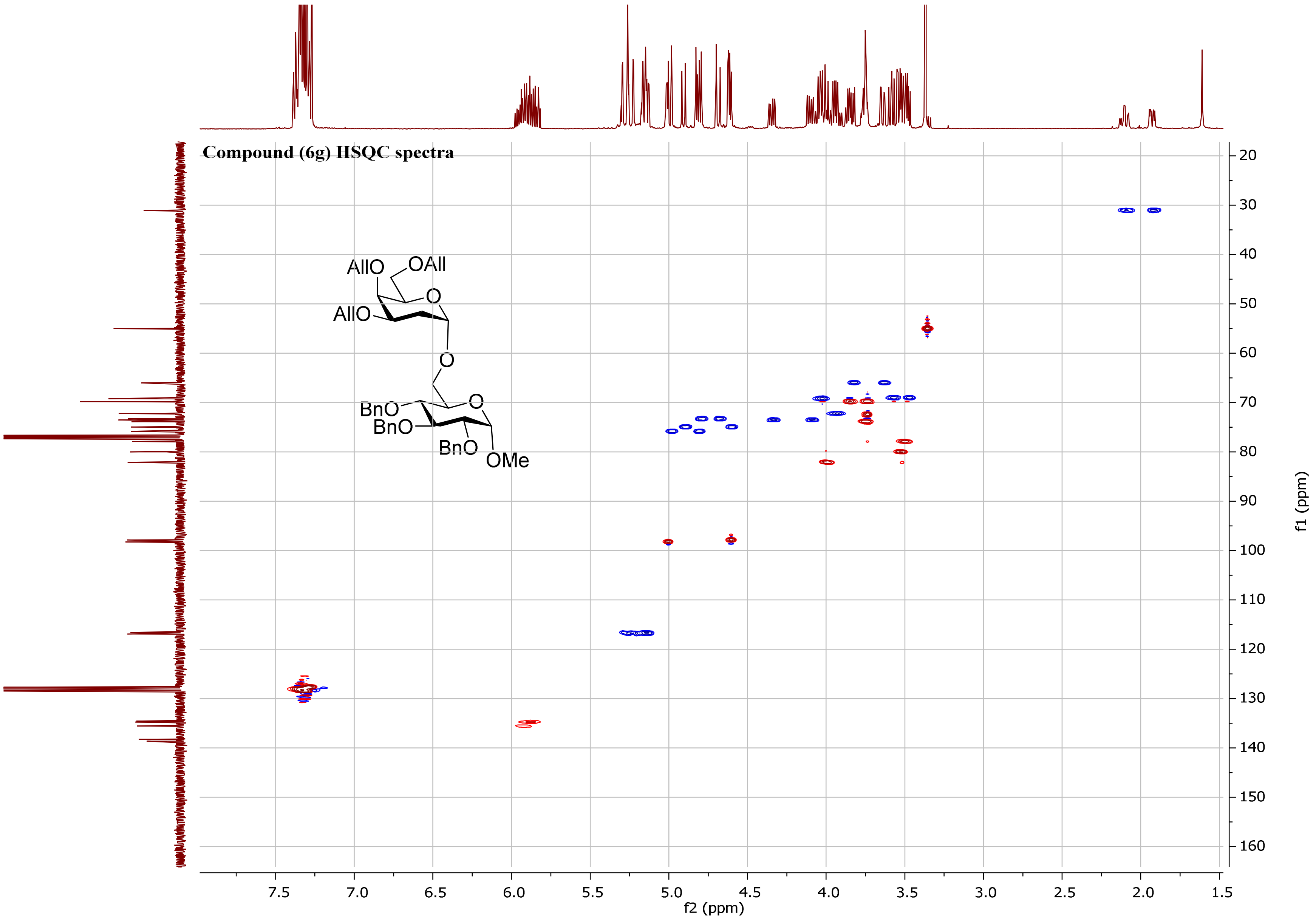
Compound (6g) HSQC spectra
AllO
OAll
O
AllO
O
BnO
BnO
O
BnO
OMe
f1 (ppm)
f2 (ppm)

Compound (7a) Proton NMR (400 MHz, Chloroform-d)

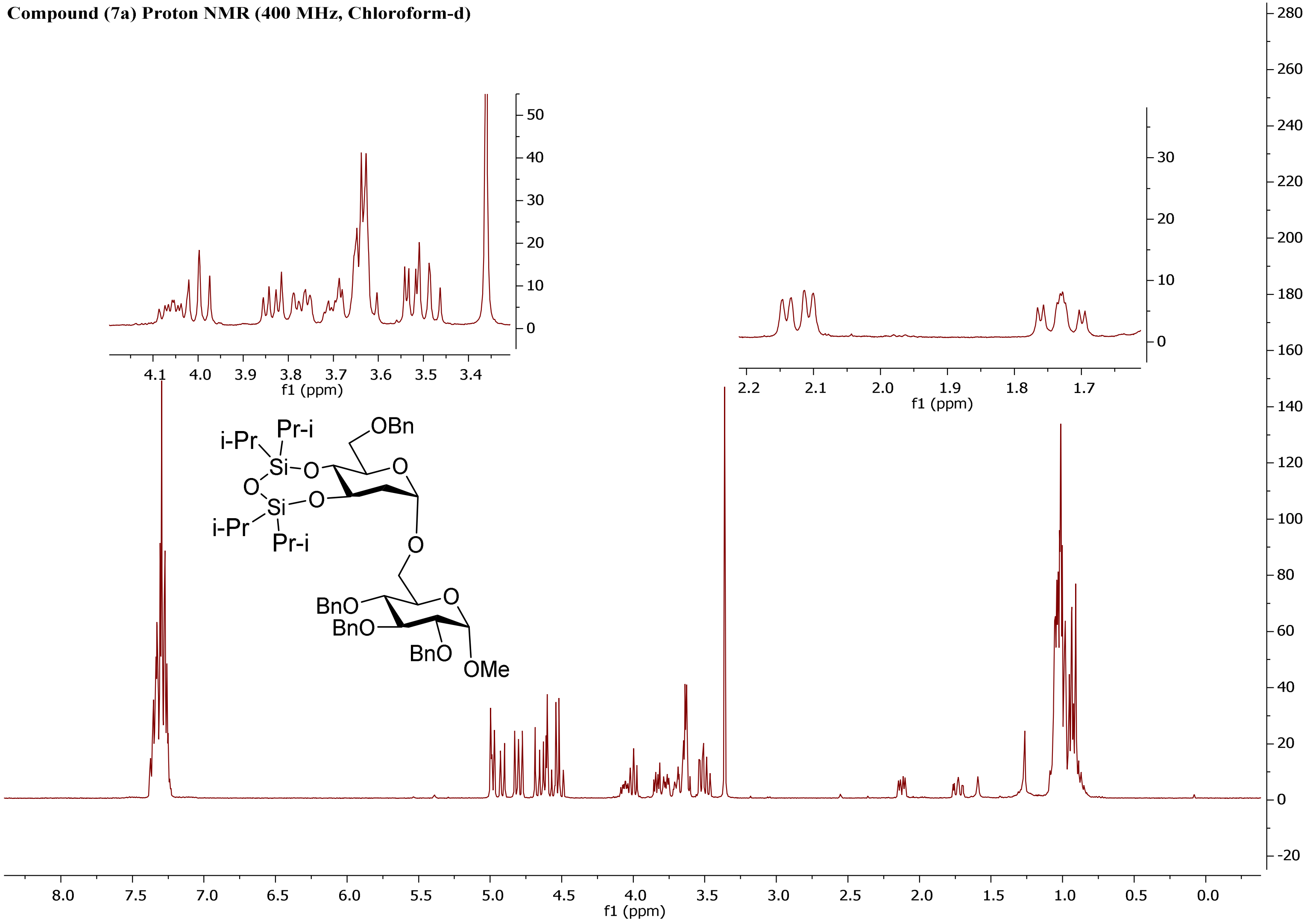

Compound
(7a) Carbon NMR (101 MHz, Chloroform-d)

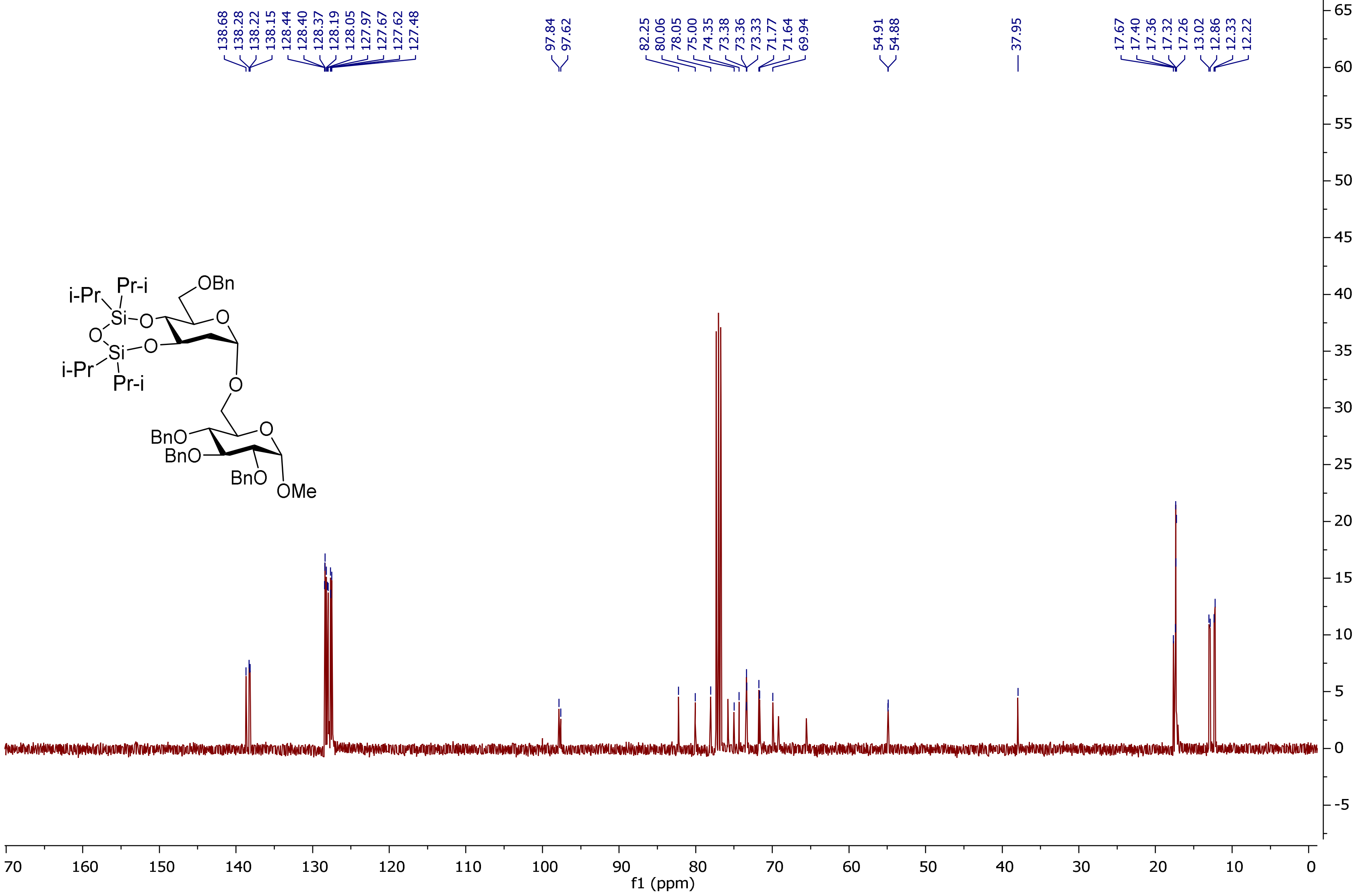

Compound (7a) HSQC spectra

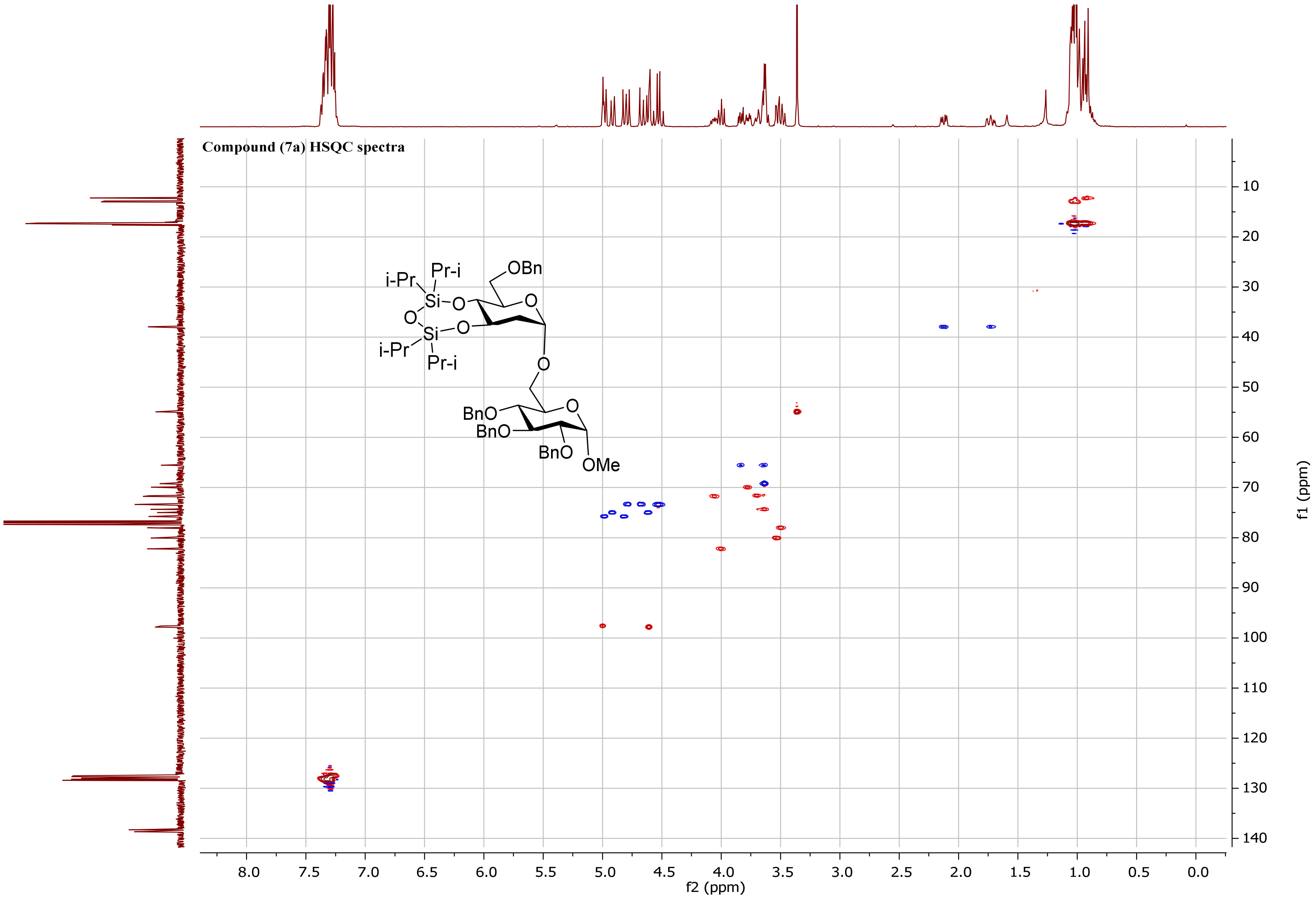

Compound (7b) Proton NMR (400 MHz, Chloroform-d)

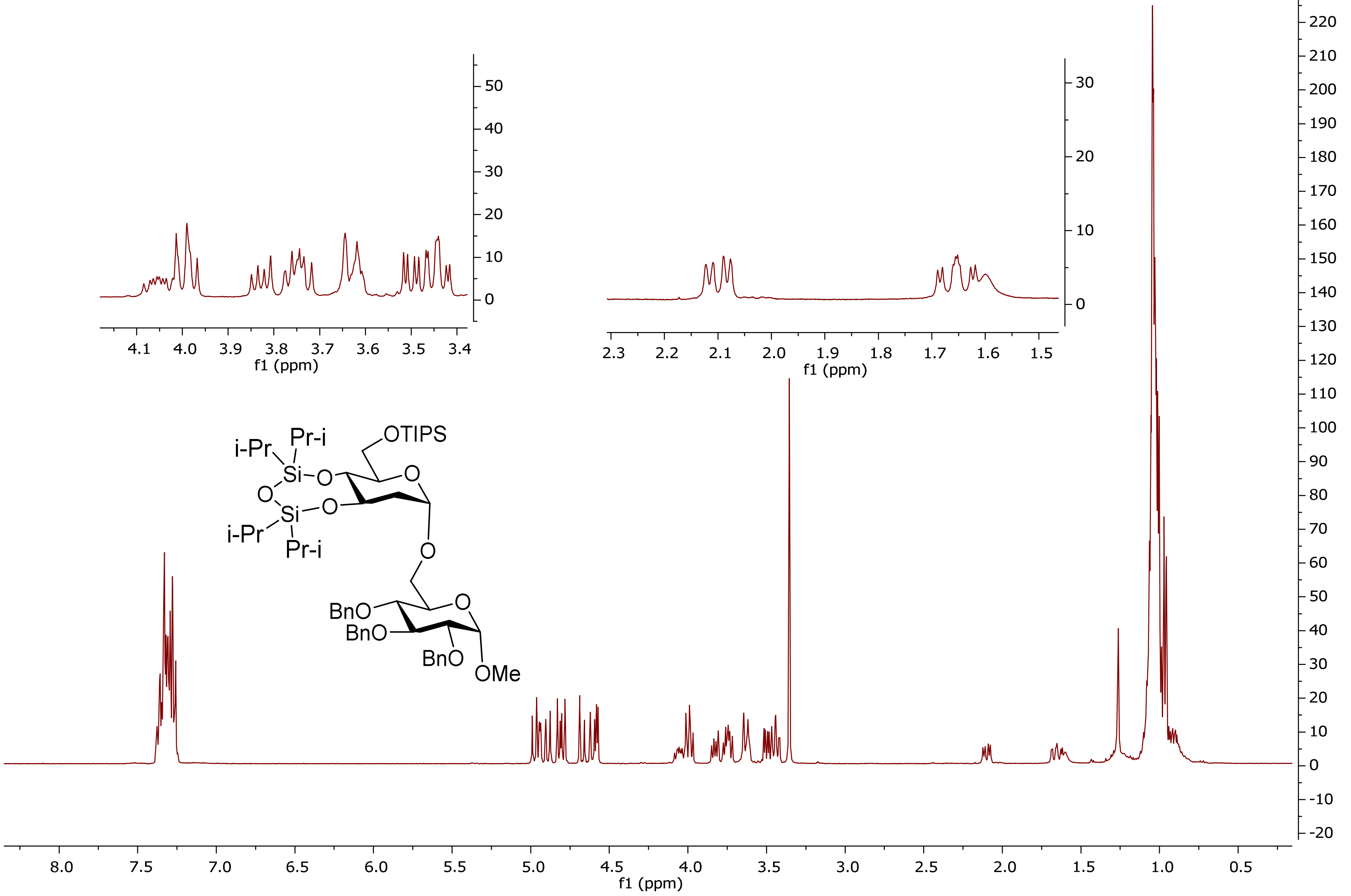

Compound (7b) Carbon NMR (101 MHz, Chloroform-d)

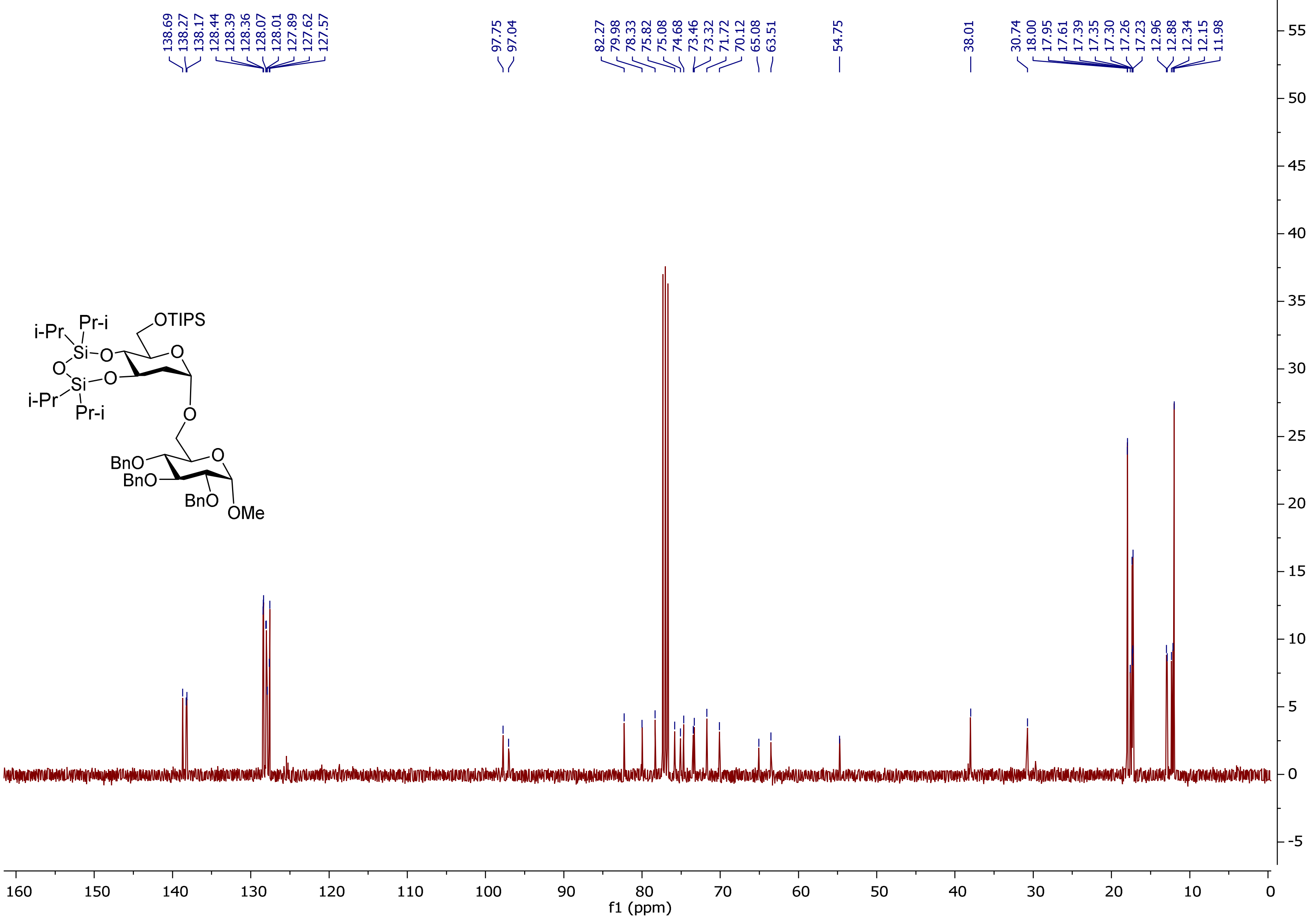

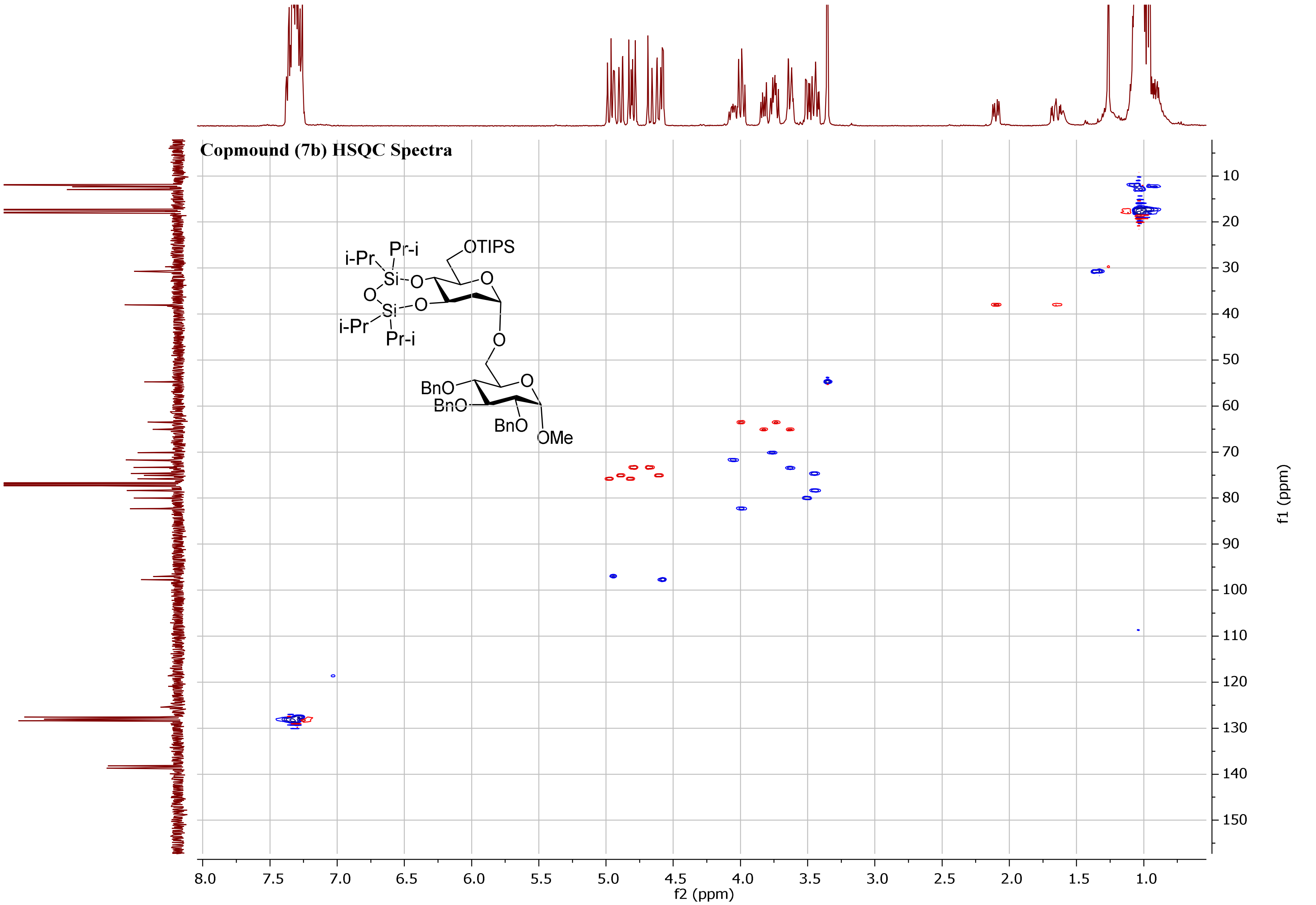
Copmound (7b) HSQC Spectra
i-Pr
Pr-i
OTIPS
Si
O
O
O
O
Si
i-Pr
Pr-i
O
BnO
BnO
O
BnO
OMe
f2 (ppm)
f1 (ppm)

Compound (7f) (proton NMR 500 Mz Chloroform-d)

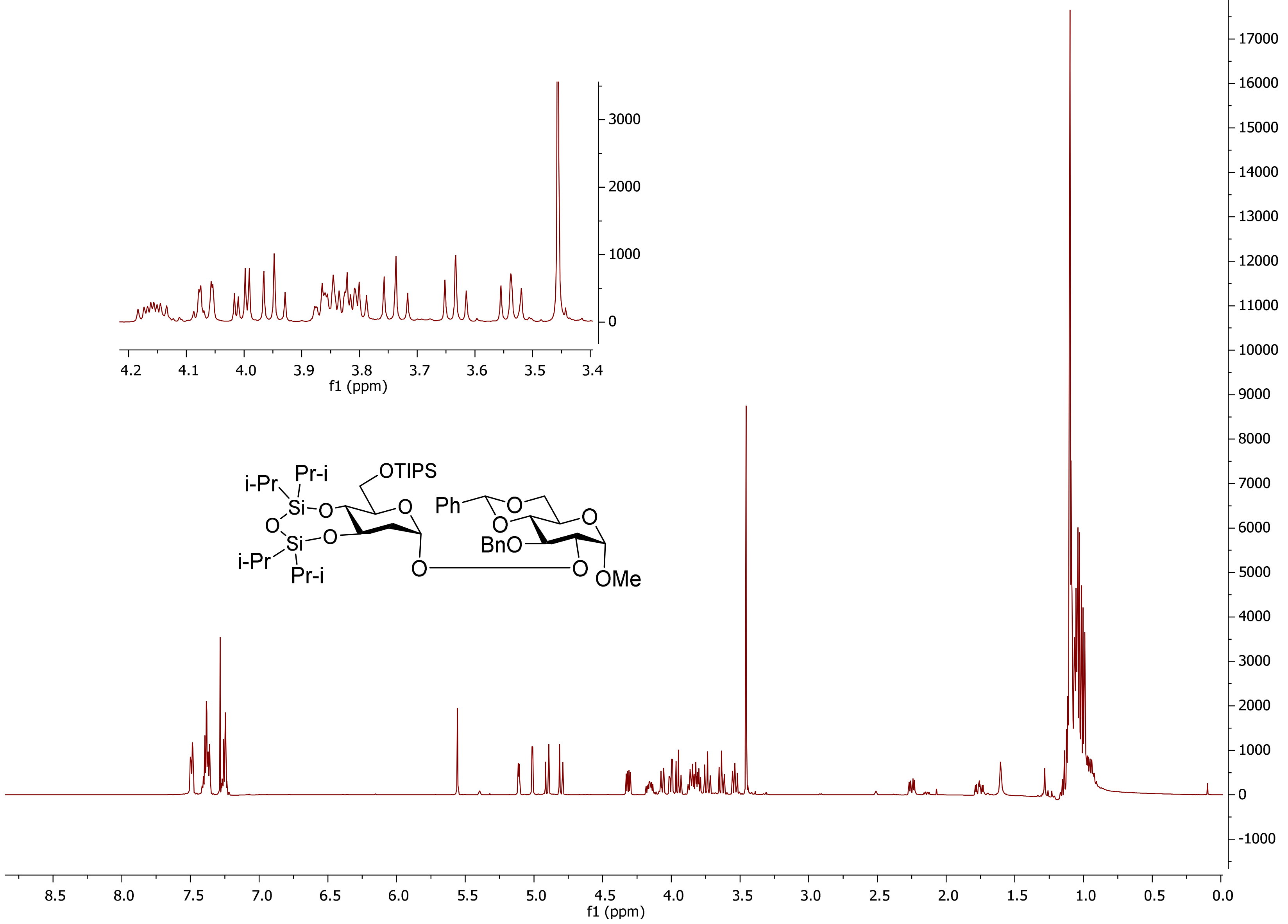

Compound (7f) Carbon NMR (101 Mz Chloroform-d)

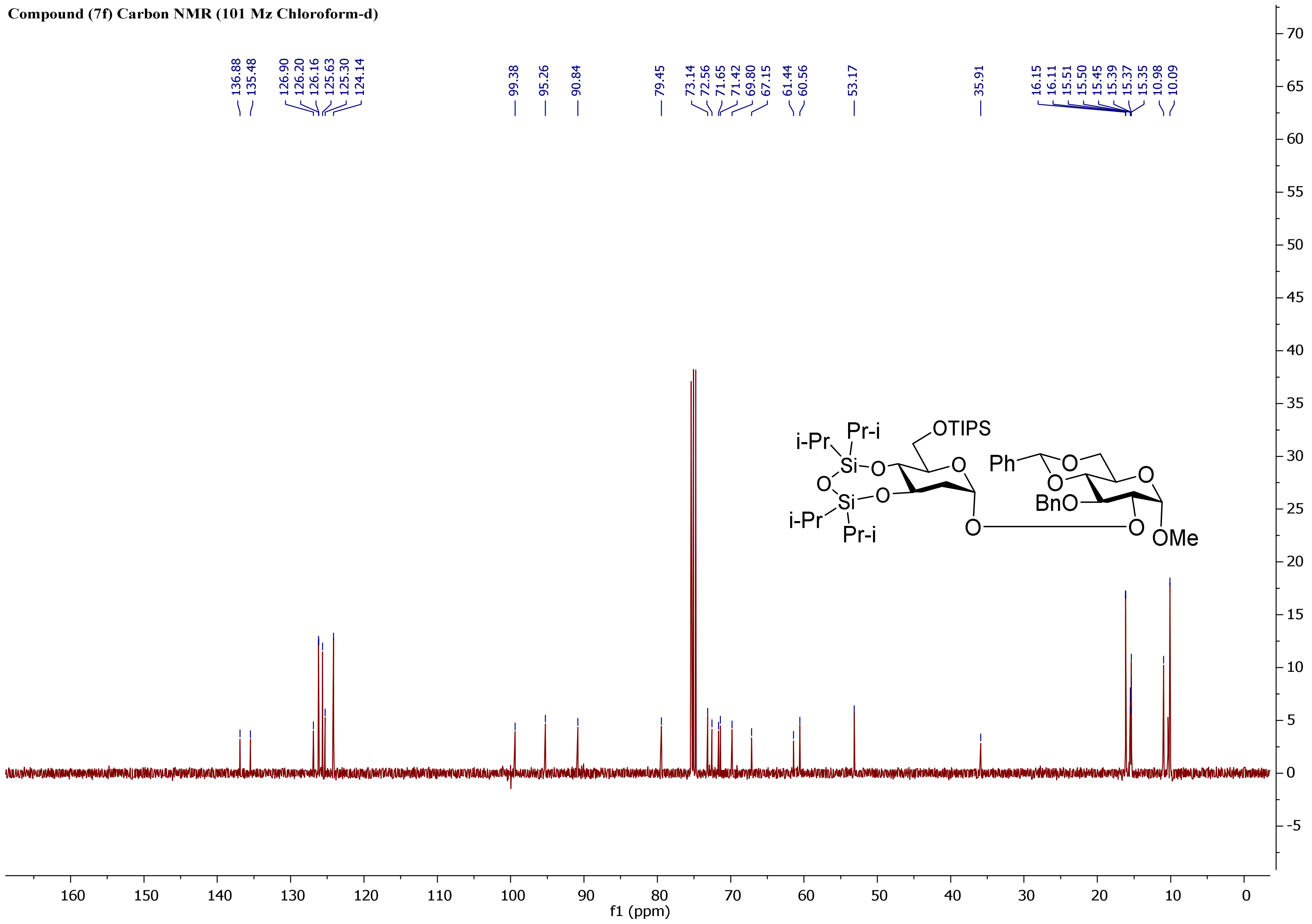

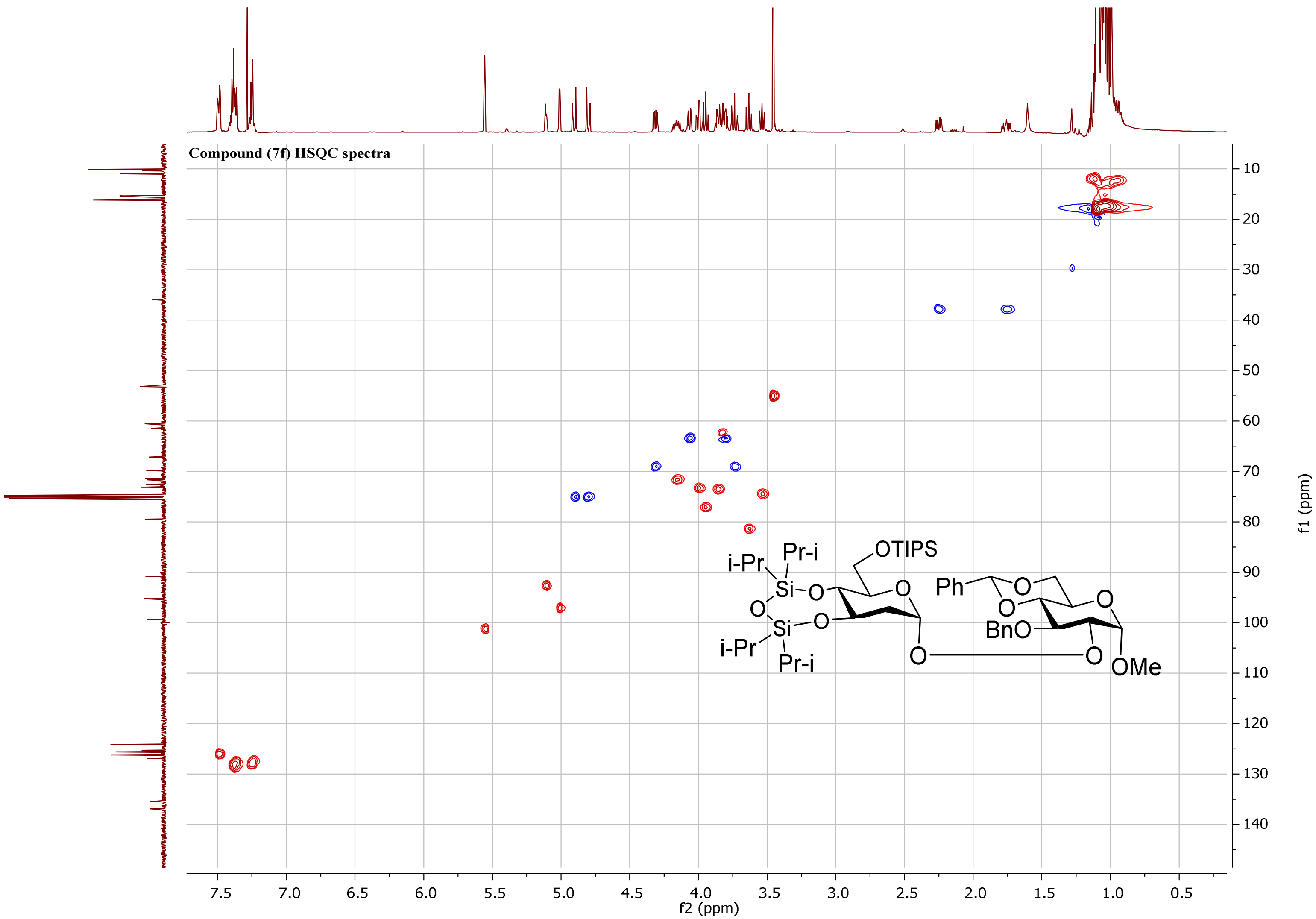
Compound (7f) HSQC spectra
f2 (ppm)
f1 (ppm)
OTIPS
i-Pr
Pr-i
Si
O
BnO
Ph
OMe

Compound (8) Proton NMR (400 MHz, Chloroform-d)

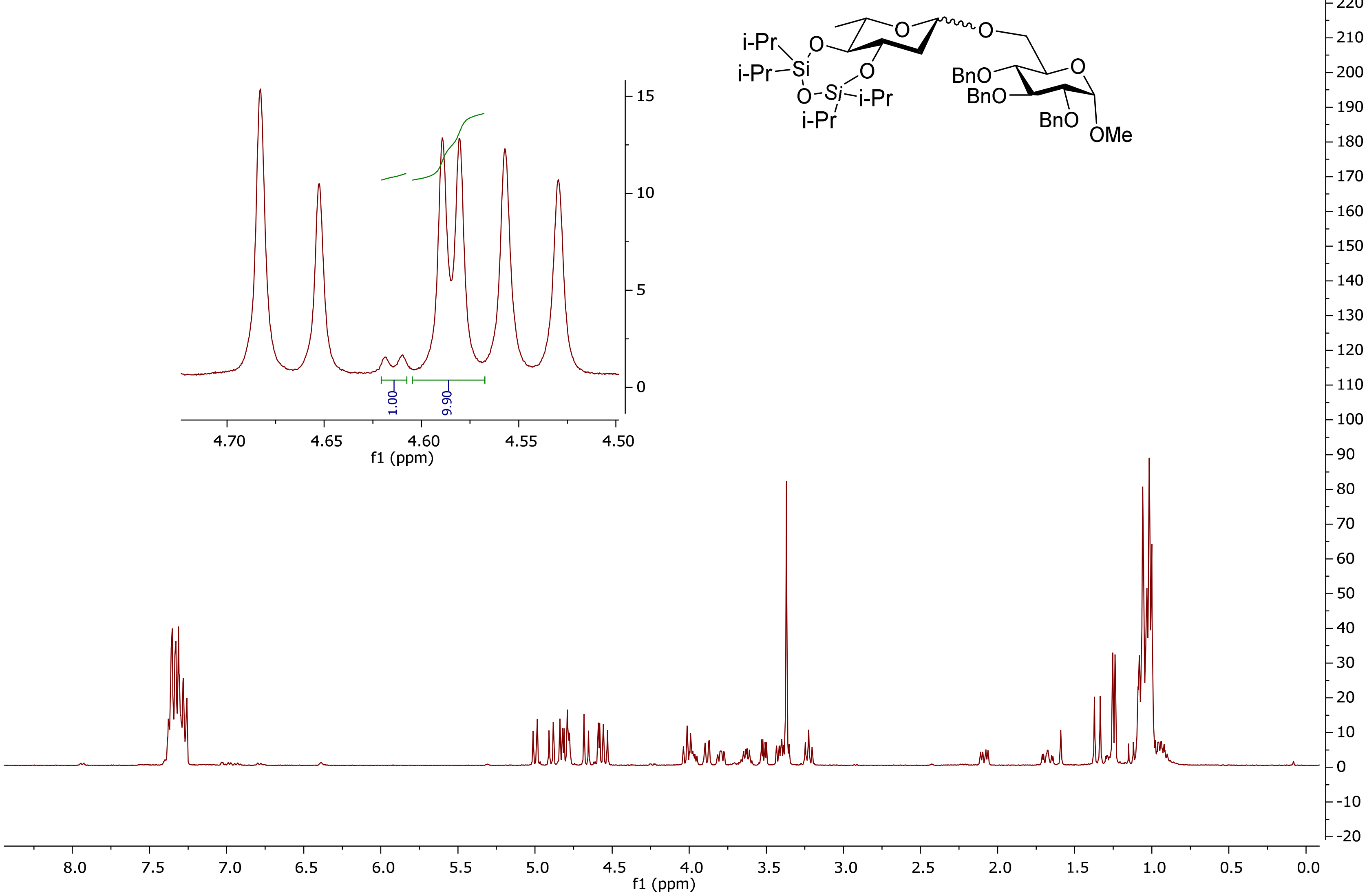

Copmound (8) Carbon NMR (101 MHz, Chloroform-d)

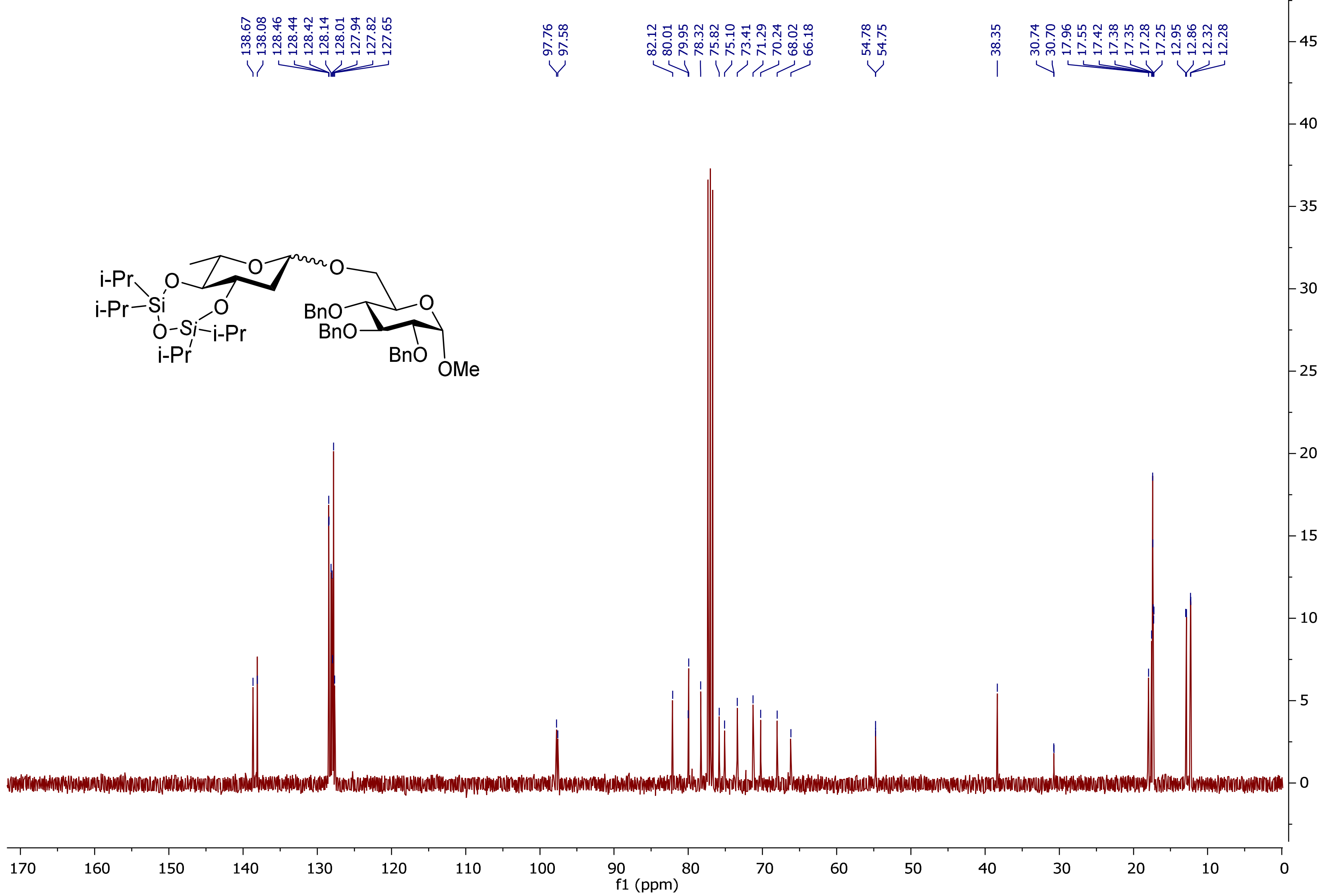

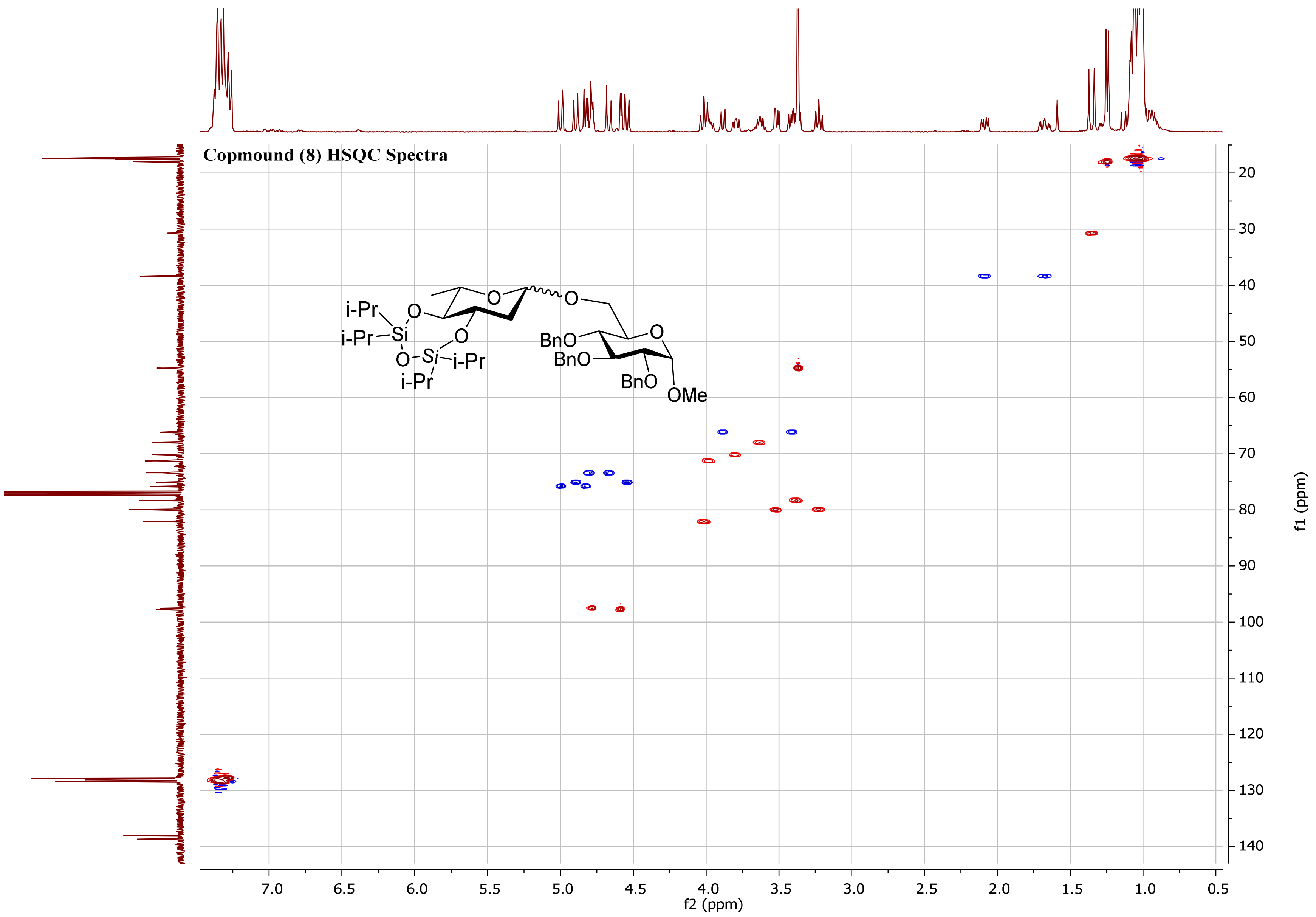
Copmound (8) HSQC Spectra
i-Pr
i-Pr
Si
O
Si
i-Pr
i-Pr
BnO
BnO
BnO
OMe
f2 (ppm)
f1 (ppm)
7.0
6.5
6.0
5.5
5.0
4.5
4.0
3.5
3.0
2.5
2.0
1.5
1.0
0.5
20
30
40
50
60
70
80
90
100
110
120
130
140

Copmound (12): Proton NMR (400 MHz,Chloroform-d)

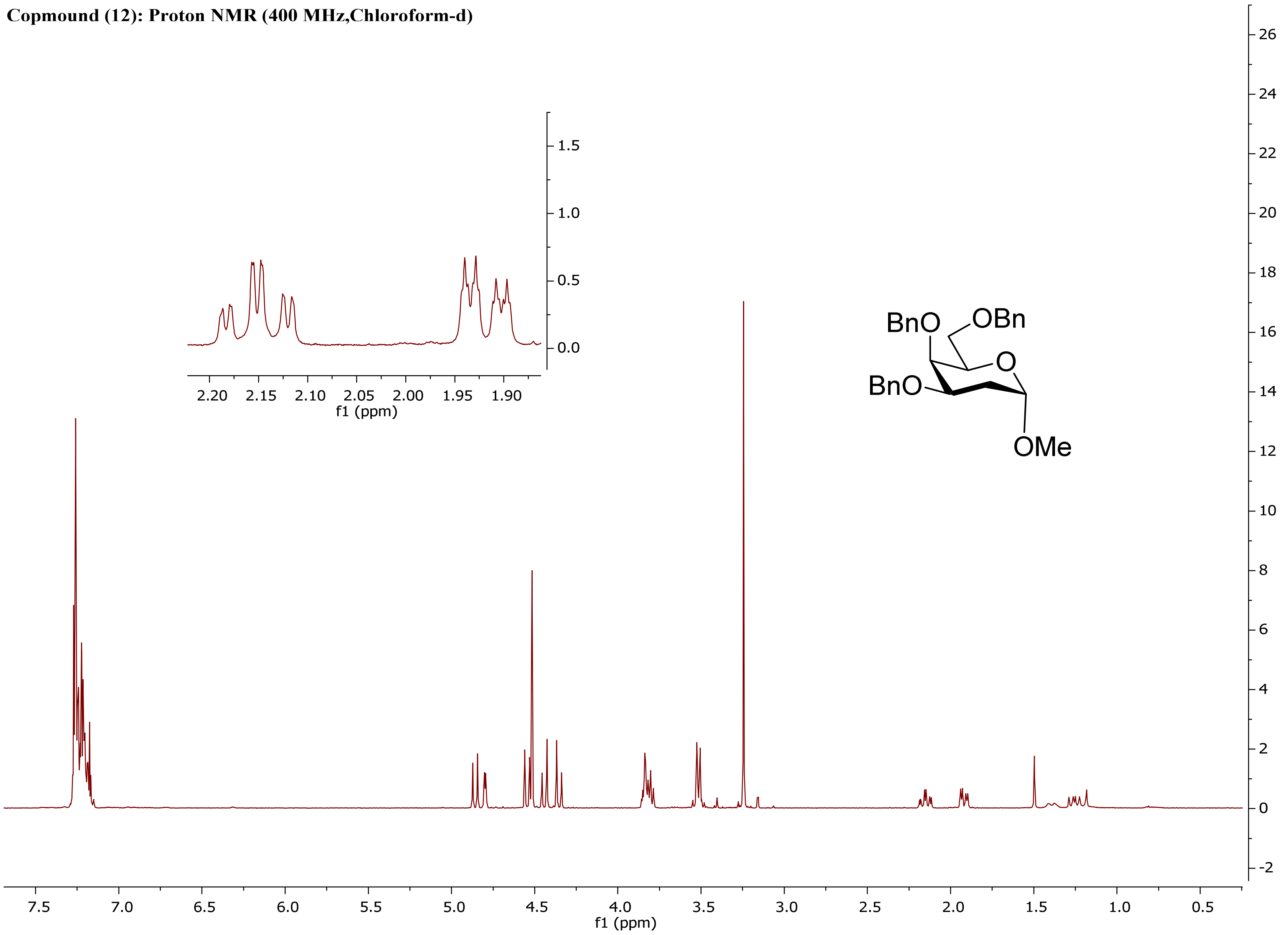

Copmound (12): Carbon NMR (400 MHz,Chloroform-d)

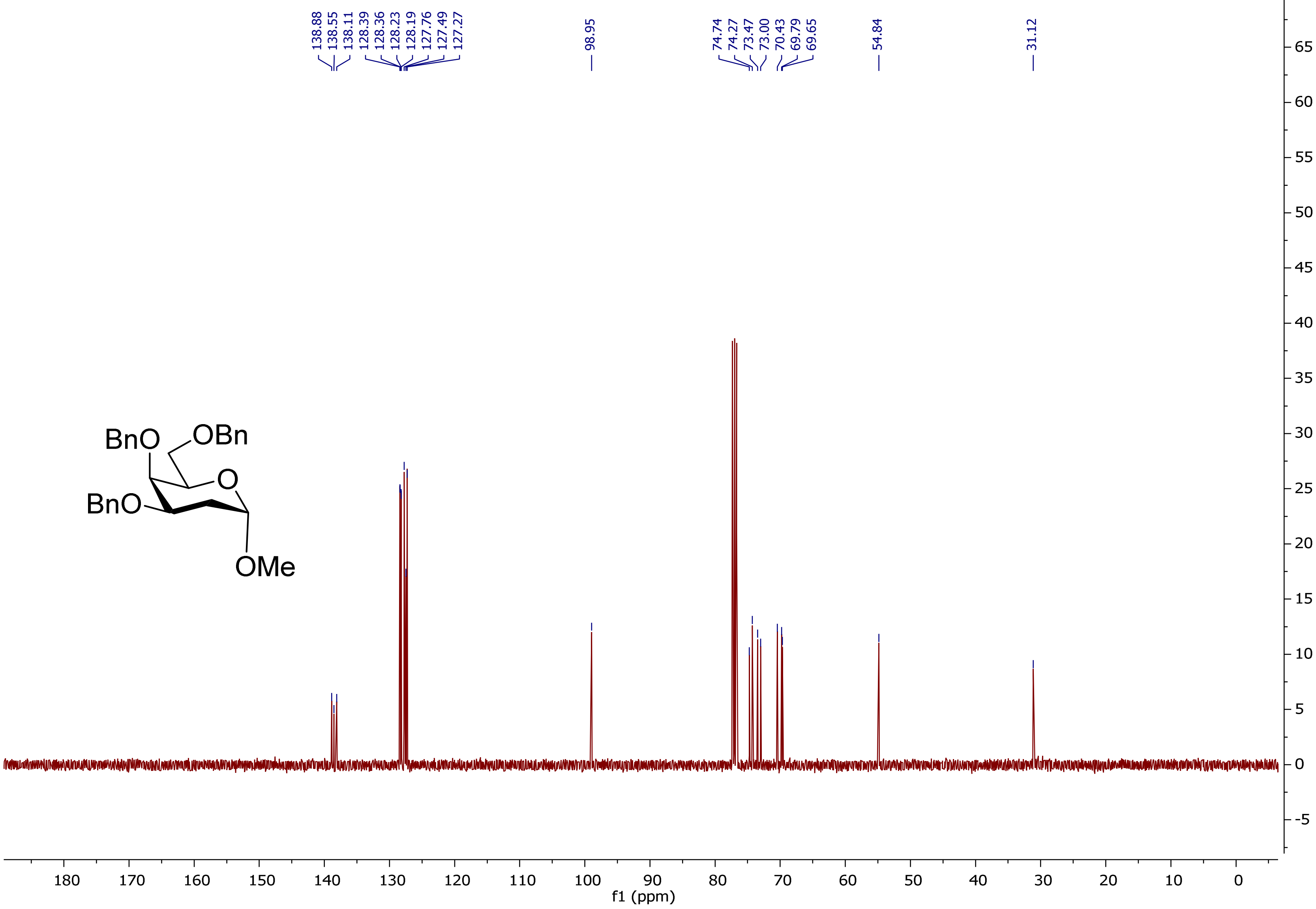

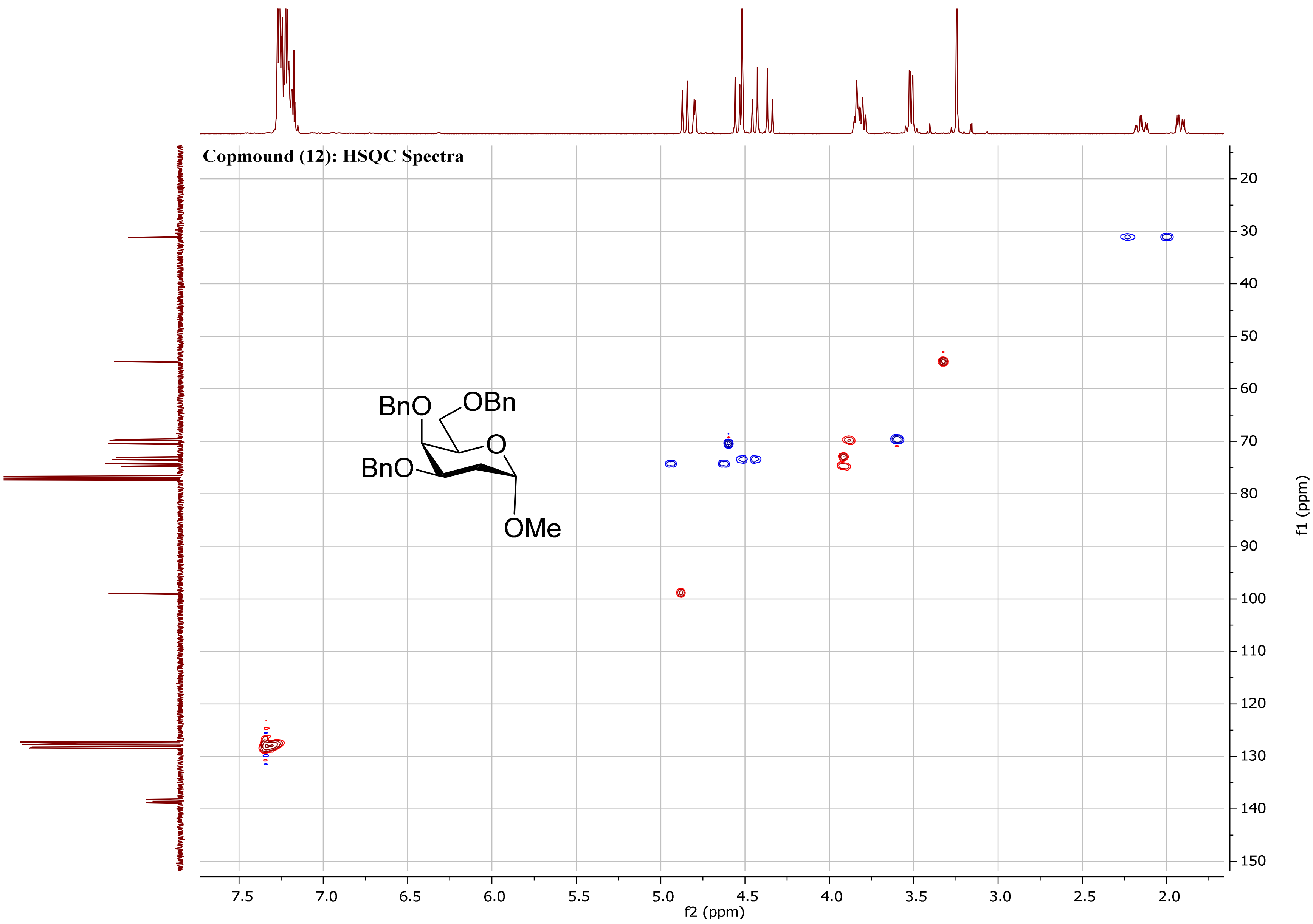
Copmound (12): HSQC Spectra
BnO
OBn
O
BnO
OMe
f2 (ppm)
f1 (ppm)

Copmound (10) Proton NMR (500 Mz, Chloroform-d)

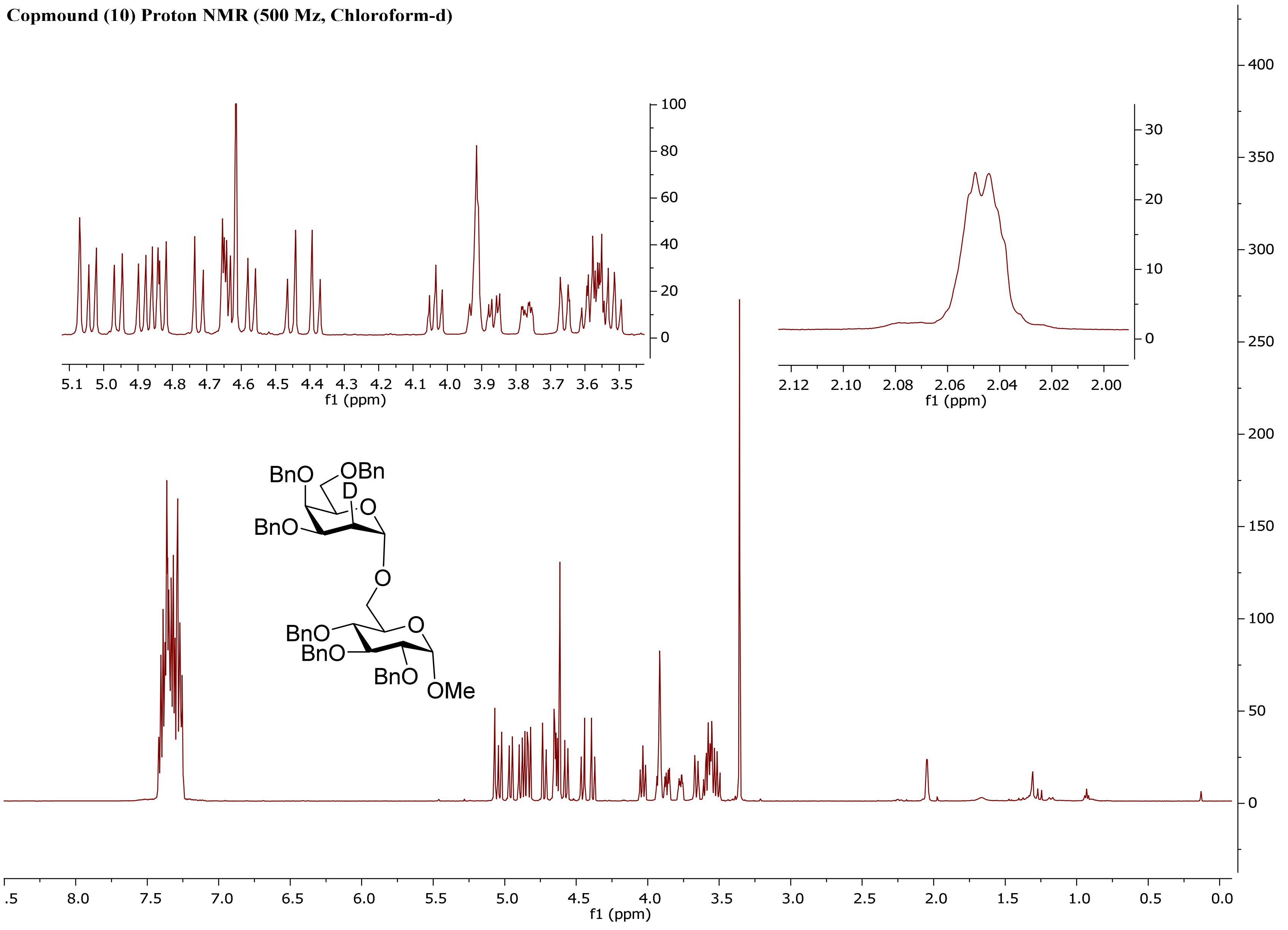

Copmound (10) Carbon NMR (101 Mz, Chloroform-d)

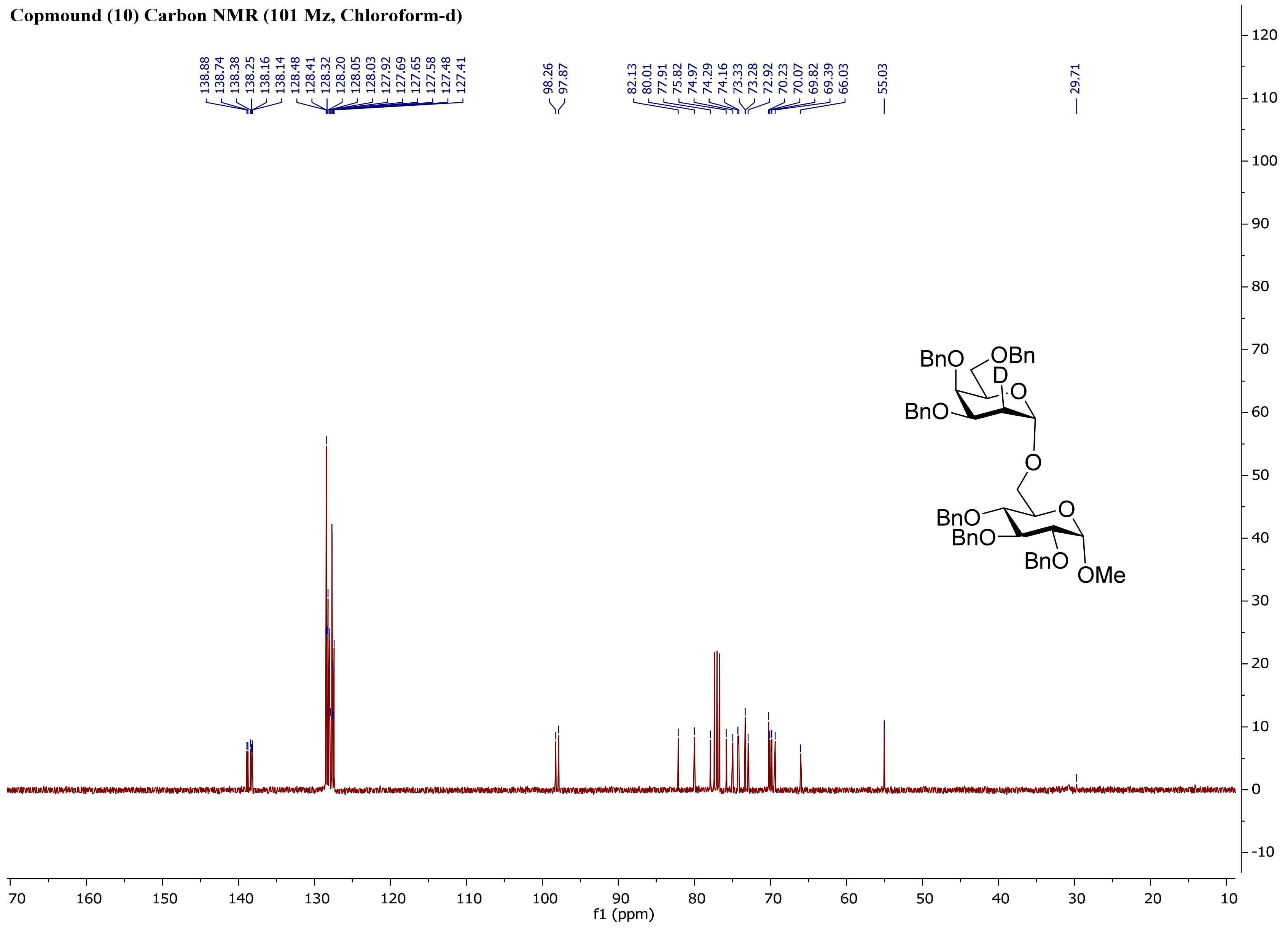

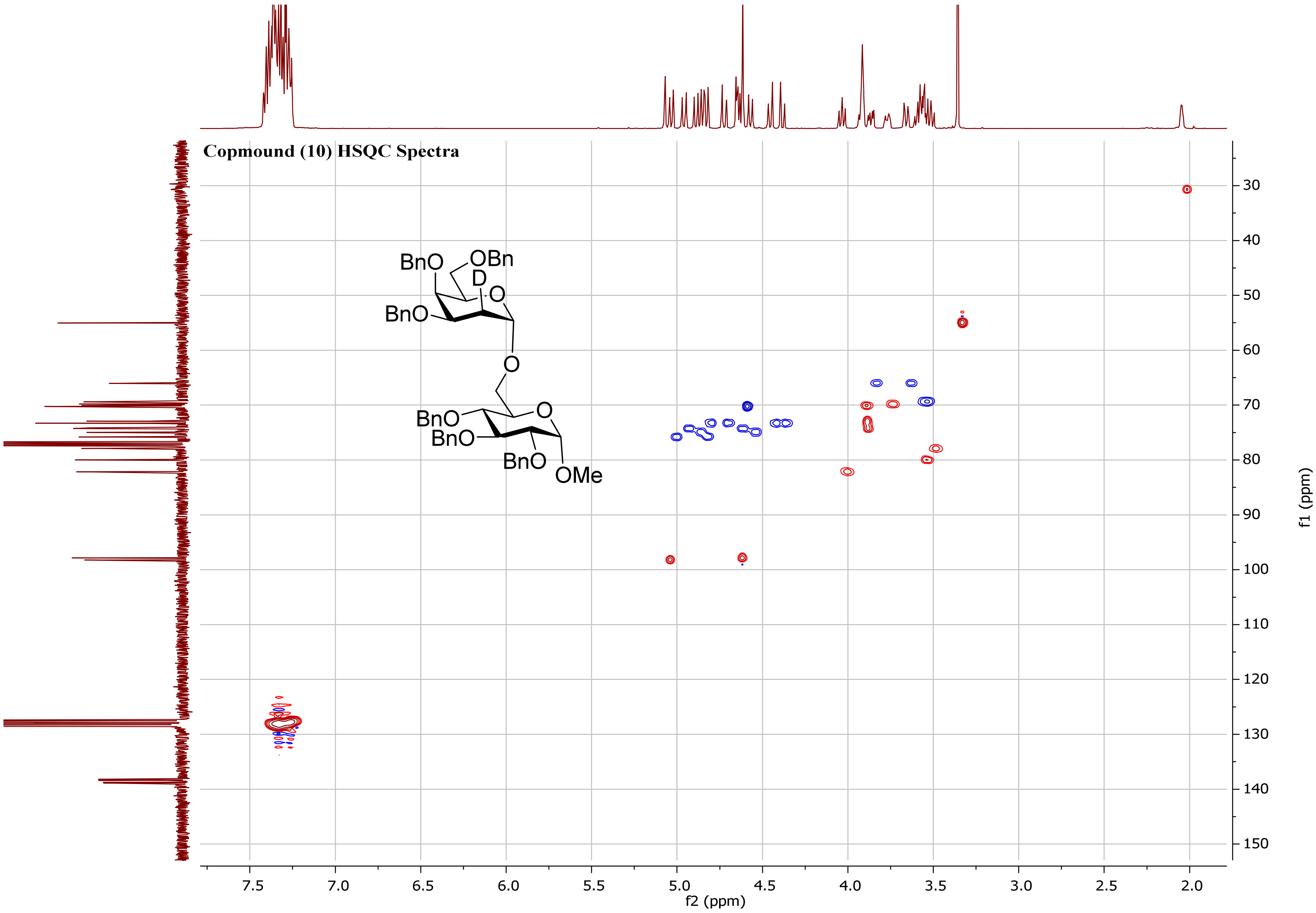

Copmound (10) HSQC Spectra
BnO
OBn
D
O
BnO
O
BnO
BnO
O
BnO
OMe
7.5
7.0
6.5
6.0
5.5
5.0
4.5
4.0
3.5
3.0
2.5
2.0
f2 (ppm)
30
40
50
60
70
80
90
100
110
120
130
140
150
f1 (ppm)

Copmound (11): Proton NMR(400 MHz, Chloroform)

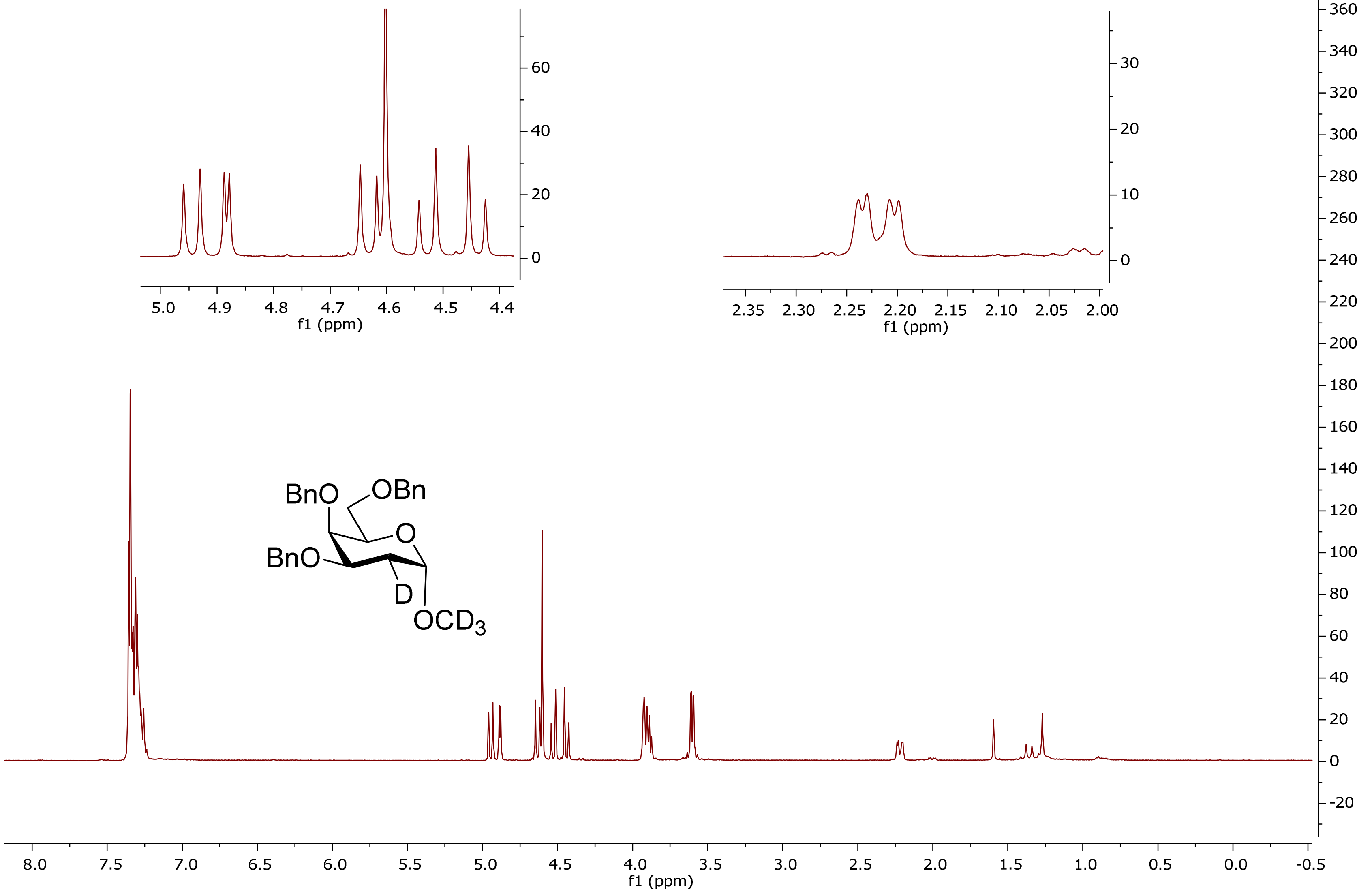

Copmound (11): Carbon NMR(101 MHz, Chloroform)

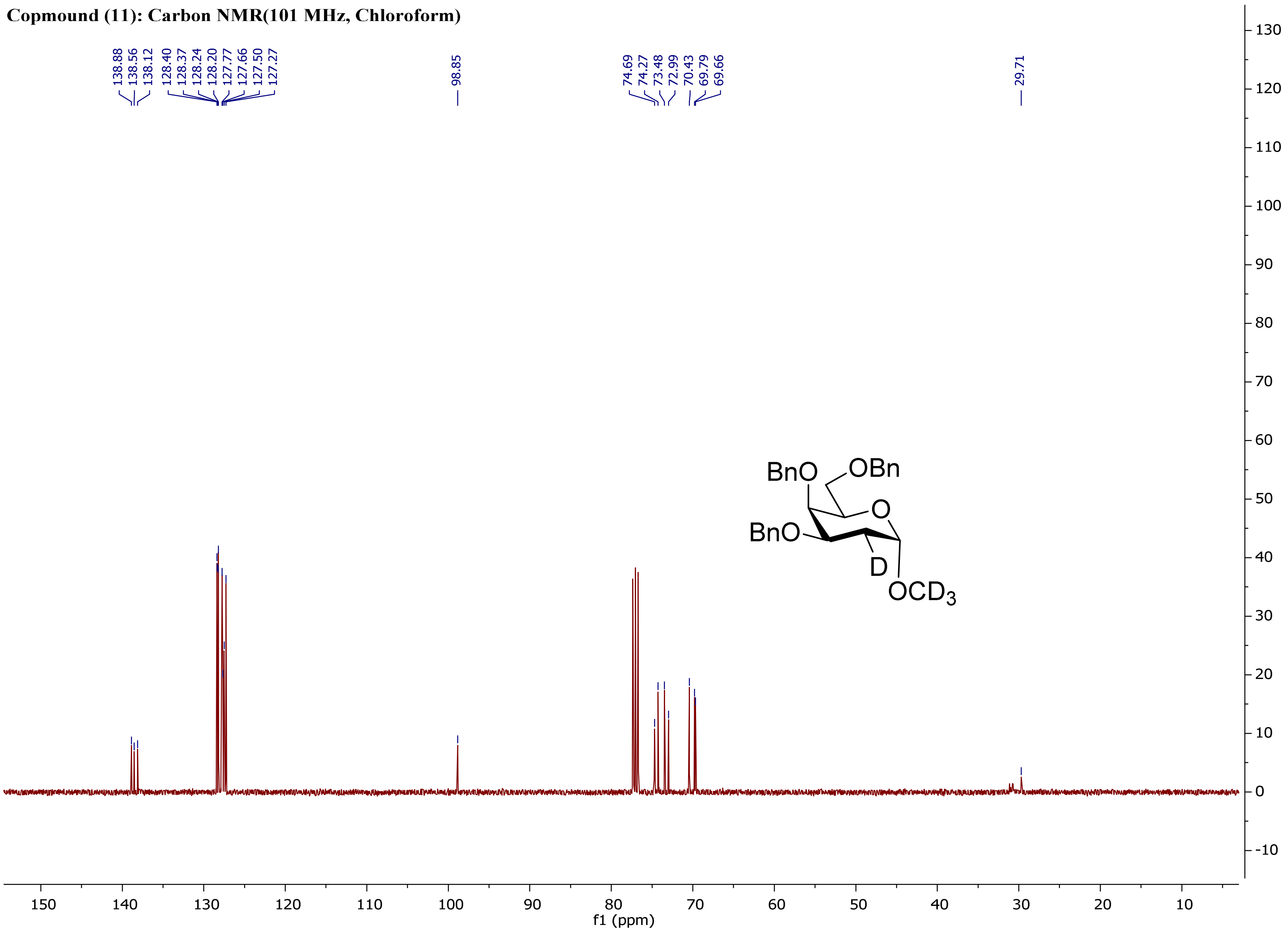

Copmound (11): HSQC Spectra

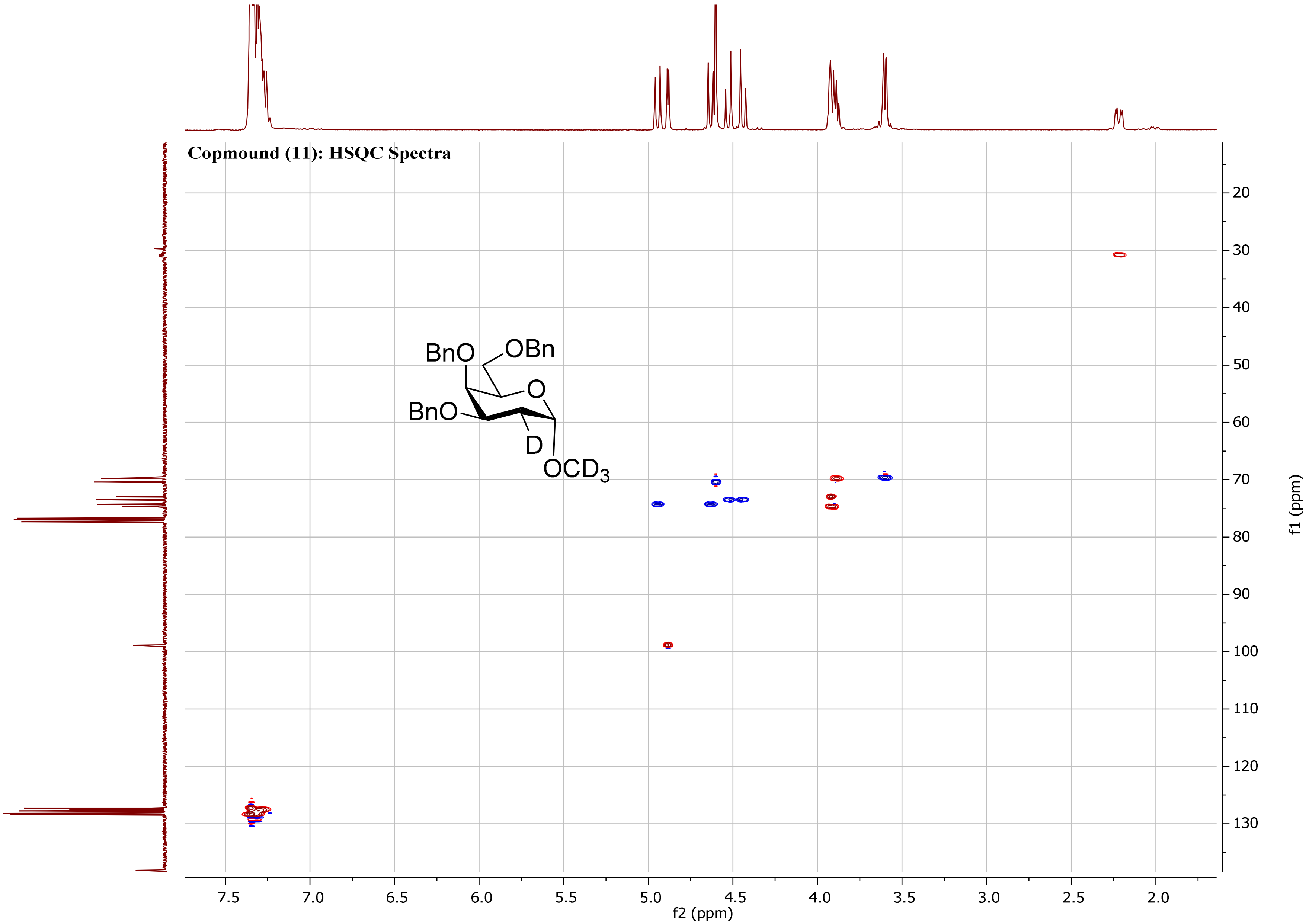

Supplement: Supplementary file 1 — Supplementary [file ANIE-56-3640-s001.pdf]
